# Mesenchymal Osr1^+^ cells regulate embryonic lymphatic vessel formation

**DOI:** 10.1242/dev.202747

**Published:** 2024-09-02

**Authors:** Pedro Vallecillo-García, Mira Nicola Kühnlein, Mickael Orgeur, Nils Rouven Hansmeier, Georgios Kotsaris, Zarah Gertrud Meisen, Bernd Timmermann, Claudia Giesecke-Thiel, René Hägerling, Sigmar Stricker

**Affiliations:** ^1^ Institute for Chemistry and Biochemistry, Freie Universität Berlin, 14195 Berlin, Germany; ^2^ Department of Hematology, Oncology and Tumorimmunology, Charité-Universitätsmedizin Berlin, Corporate Member of Freie Universität Berlin and Humboldt-Universität zu Berlin, 13353 Berlin, Germany; ^3^ Unit for Integrated Mycobacterial Pathogenomics, Institut Pasteur, Université Paris Cité, CNRS UMR 6047, 75015 Paris, France; ^4^ Research Group ‘Lymphovascular Medicine and Translational 3D-Histopathology’, Institute of Medical and Human Genetics, Charité-Universitätsmedizin Berlin, Augustenburger Platz 1, 13353 Berlin, Germany; ^5^ BIH Center for Regenerative Therapies, Berlin Institute of Health at Charité-Universitätsmedizin Berlin, Augustenburger Platz 1, 13353 Berlin, Germany; ^6^ Research Group ‘Development and Disease’, Max Planck Institute for Molecular Genetics, Ihnestraße 63-73, 14195 Berlin, Germany; ^7^ Max Planck Institute for Molecular Genetics, 14195 Berlin, Germany; ^8^ BIH Academy, Clinician Scientist Program, Berlin Institute of Health at Charité-Universitätsmedizin Berlin, Charitéplatz 1, 10117 Berlin, Germany

**Keywords:** Cxcl12, Osr1, Extracellular matrix, Lymphatic vasculature, Mesenchymal cell

## Abstract

The lymphatic system is formed during embryonic development by the commitment of specialized lymphatic endothelial cells (LECs) and their subsequent assembly in primary lymphatic vessels. Although lymphatic cells are in continuous contact with mesenchymal cells during development and in adult tissues, the role of mesenchymal cells in lymphatic vasculature development remains poorly characterized. Here, we show that a subpopulation of mesenchymal cells expressing the transcription factor Osr1 are in close association with migrating LECs and established lymphatic vessels in mice. Lineage tracing experiments revealed that Osr1^+^ cells precede LEC arrival during lymphatic vasculature assembly in the back of the embryo. Using *Osr1*-deficient embryos and functional *in vitro* assays, we show that *Osr1* acts in a non-cell-autonomous manner controlling proliferation and early migration of LECs to peripheral tissues. Thereby, mesenchymal Osr1^+^ cells control, in a bimodal manner, the production of extracellular matrix scaffold components and signal ligands crucial for lymphatic vessel formation.

## Teaser

**Summary:** Mesenchymal Osr1^+^ cells regulate embryonic lymphatic vessel formation by controlling extracellular matrix and guidance signaling molecules.

## INTRODUCTION

The lymphatic vasculature establishes a blind-ended hierarchical network of vessels with crucial roles in interstitial fluid homeostasis, immune cell response and lipid metabolism. In peripheral tissues, lymphatic vessels form thin-walled capillaries that drain interstitial fluid and facilitate the transport of macromolecules and cells into larger pre-collecting lymphatic vessels. Here, the lymph is moved unidirectionally by the cooperative action of lymphatic valves, the synchronic contraction of lymphatic vessel-associated mural cells and passively by the force generated in surrounding tissues such as skeletal muscles and arteries. Finally, the lymph is transported into the blood stream at the subclavian vein (Tammela and Alitalo, 2010; Schulte-Merker et al., 2011; Vaahtomeri et al., 2017).

Lymphatic vessels are formed by specialized endothelial cells, the lymphatic endothelial cells (LECs), which originate from venous and non-venous tissues depending on the vascular bed (Jafree et al., 2021; Lioux et al., 2020; Stanczuk et al., 2015). By far the best understood process is the formation of LECs via dedifferentiation of embryonic venous endothelial cells. In mice, reprogramming of venous cells towards an LEC fate occurs at embryonic day (E)9-E10, relying on the activation of key LEC transcription factors such as prospero homeobox1 (PROX1) and SRY-related HMG-box 18 (SOX18), and is associated with the expression of markers such as lymphatic vessel endothelial hyaluronan receptor 1 (LYVE1) (Tammela and Alitalo, 2010; Schulte-Merker et al., 2011; Francois et al., 2008). After this initial step in LEC commitment, which occurs at the dorsal part of the cardinal vein, LECs delaminate and migrate into the surrounding mesenchyme, forming transient primordial lymphatic vascular structures called primordial thoracic ducts, formerly known as lymph sacs, which can be observed in the mouse embryo during the stages E11.5-E14.5 (Tammela and Alitalo, 2010; Schulte-Merker et al., 2011; Alitalo et al., 2005; Oliver, 2004). LEC initial migration from the cardinal vein is dependent on the production of vascular endothelial growth factor C (VEGFC) and the activation of its primary receptor fms-related tyrosine kinase 4 (FLT4), also known as VEGFR3 (Karkkainen et al., 2004; Hagerling et al., 2013). Activation of canonical or non-canonical VEGFR3-signaling, and enzymes controlling the proteolytic activation of VEGFC, play an essential role in LEC initial migration as well as in development and maintenance of lymphatic vessels (Karkkainen et al., 2004; Wang et al., 2020; Janssen et al., 2016; Jeltsch et al., 2014; Koltowska et al., 2015). In addition, directed migration of LECs is controlled by the CXCL12/CXCR4 chemokine axis (Peng et al., 2022; Niimi et al., 2020; Cha et al., 2012). Both *Vegfc* and *Cxcl12* are highly expressed by embryonic mesenchymal cells adjacent to the developing lymphatic vasculature (Karkkainen et al., 2004; Wang et al., 2020; Peng et al., 2022; Vallecillo-Garcia et al., 2023). Despite the knowledge gained on mechanisms supporting LEC migration in recent years (Shiiya and Hirashima, 2023), little is known about how the final pattern of lymphatic vessels in different tissues of the embryo is achieved. Several cell types have been described to influence lymphatic vessel development in zebrafish and mice, such as platelets, arterial endothelial cells, neurons, myeloid cells and mural cells (Wang et al., 2020; Peng et al., 2022; Bussmann et al., 2010; Uchida et al., 2015). However, embryonic mesenchymal cells adjacent to lymphatic vessels have remained understudied, in part due to the lack of specific markers that label mesenchymal cell subpopulations during development. Mesenchymal cells are important producers of the extracellular matrix (ECM) scaffold during embryogenesis and adult life (Walma and Yamada, 2020). Of note, LEC-ECM interactions and biophysical properties of the ECM associated with lymphatic vessels influence lymphatic vessel development and function (Frye et al., 2018; Wiig et al., 2010).

The transcription factor Osr1 is expressed in a variety of mesenchymal cells derived from the lateral plate and intermediate mesoderm (Mugford et al., 2008; Wang et al., 2005). In the limb, Osr1^+^ mesenchymal cells are present before skeletal muscle progenitors colonize the limb bud mesenchyme, and they produce guidance and a proper ECM scaffold for skeletal muscle patterning (Vallecillo-Garcia et al., 2017). *Osr1* expression decreases in late fetal stages of development but is reactivated after tissue damage (Vallecillo-Garcia et al., 2023, 2017; Stumm et al., 2018; Kotsaris et al., 2023). We recently showed that *Osr1* is required in mesenchymal cells to organize lymph node lymphatic vasculature assembly, and that *Osr1*-expressing cells cooperate with LECs in the lymph node anlage driving lymph node initiation (Vallecillo-Garcia et al., 2023). This suggests a crosstalk between mesenchymal Osr1^+^ cells and LECs as an important event for lymphatic vessel formation. Moreover, the lymphatic vasculature can be remodeled in pathogenic conditions such as inflammation, wound healing, tumor formation, hypertension or tissue transplantation (Jafree et al., 2021; Pichol-Thievend et al., 2018), where mesenchymal cell-LEC interactions might be necessary to achieve lymphangiogenesis. Nevertheless, the role of these mesenchymal *Osr1*-expressing cells or the ECM produced by them in the formation of murine lymphatic vasculature remains obscure.

Here, we show that mesenchymal cells expressing the transcription factor *Osr1* are in close association with the developing venous-derived lymphatic endothelial cells. Functionally, the lack of *Osr1* revealed a non-cell-autonomous function of embryonic Osr1^+^ mesenchymal cells that control lymphatic vessel formation by producing a beneficial ECM scaffold and signaling molecules necessary for LEC proliferation and directed migration.

## RESULTS

### Osr1^+^ mesenchymal cells accompany LECs during development and in adult tissues

**Fig. 1.**
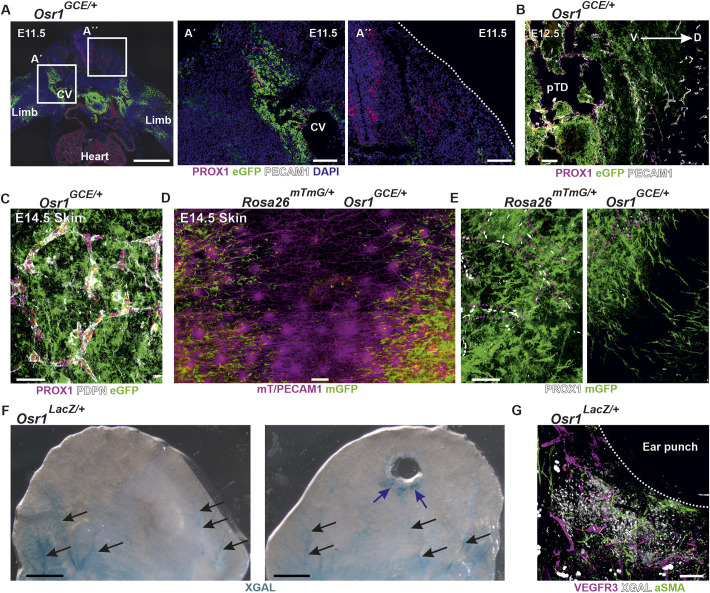
**Mesenchymal Osr1^+^ cells accompany lymphatic endothelial cells during development.** (A) Immunofluorescence of E11.5 *Osr1^GCE/+^* cross-section shows Osr1^+^ cells (eGFP) in the migration path of delaminating LECs labeled by PROX1 and PECAM1 dorsally of the cardinal vein. Boxed regions are shown at higher magnification at the right (A′,A″). (B) E12.5 sagittal section of *Osr1^GCE/+^* embryos show primordial thoracic duct surrounded by Osr1^+^ mesenchymal cells. (C) E14.5 whole-mount immunofluorescence shows lymphatic vasculature labeled by PDPN and PROX1 embedded in Osr1^+^ dermal fibroblasts in the dorsal skin of E14.5 *Osr1^GCE/+^* embryos. (D,E) Whole-mount immunofluorescence of E14.5 *Rosa26^mTmG/+^ Osr1^GCE/+^* dorsal skin shows the distribution of lineage traced Osr1 mesenchymal cells (mGFP). Stripes of Osr1-traced cells are localized ahead of blood vasculature labeled with mT/PECAM1 and lymphatic vasculature labeled with PROX1. Dashed lines represent the border of lymphatic vessels in E. (F) Whole-tissue X-gal staining reveals Osr1 expression in the dermal side of the ear from adult *Osr1^lacZ^* reporter animals in contralateral (left) and ear punch-injured (right). Blue arrows point to activated Osr1 expression in the injury area and black arrows to Osr1 expression in association with established vessels. (G) Osr1 expression close to the ear punch injury in adult *Osr1^lacZ^* reporter animals assessed by whole-tissue X-gal staining. Dashed lines represent the border of the regenerating tissue. Representative immunofluorescence images have been captured from at least three different animals. CV, cardinal vein; D, dorsal; pDT, primordial thoracic duct; V, ventral. Scale bars: 500 µm (A); 100 µm (A′,A″,B,G); 50 µm (C,E); 200 µm (D); 2 mm (F).

Impaired lymphatic vasculature assembly in embryonic lymph node anlage and back lymphedema found in *Osr1*-deficient embryos indicates a more general role of Osr1^+^ mesenchymal cells in lymphatic vasculature formation (Vallecillo-Garcia et al., 2023; Wang et al., 2005). Therefore, we analyzed *Osr1* expression and distribution of Osr1^+^ cells in association with the developing lymphatic vasculature. Using an Osr1-GFP reporter mouse line (*Osr1^GCE/+^*) (Mugford et al., 2008), we identified Osr1^+^ cells in close association with LECs during the first stages of lymphatic vessel development. At E11.5, Osr1^+^ cells were found in the mesenchyme populated by delaminating and migrating PROX1^+^ LECs dorsal and ventral of the cardinal vein (Fig. 1A,A′; Fig. S1A). Of note, at E11.5, Osr1^+^ cells were not found in dorsal skin tissues (Fig. 1A″). At E12.5, we observed that the primordial thoracic duct was surrounded by a mesenchyme rich in Osr1^+^ cells (Fig. 1B). At E14.5, when the first lymphatic vascular structures are established in the dermis of the back, whole-mount immunofluorescence of E14.5 *Osr1^GCE/+^* isolated skin showed dermal lymphatic vasculature embedded by Osr1^+^ dermal fibroblasts (Fig. 1C; Fig. S1B).

In order to follow Osr1^+^ mesenchymal cell descendants and their contribution to the mesenchyme adjacent to blood and lymphatic vasculature, we performed whole-mount immunofluorescence of E14.5 *Rosa26^mTmG/+^ Osr1^GCE/+^* back skin after tamoxifen induction at E11.5. Genetic tracing of E11.5 Osr1^+^ mesenchymal cells (schematic depiction in Fig. S1C) showed contribution to dermal fibroblasts between blood and lymph vessels (Fig. 1D), to mural smooth muscle actin-expressing cells (αSMA^+^) in arteries, and to cells associated with veins in the skin (Fig. S1C). In the back skin, Osr1^+^ descendants were found in the lymphatic avascular midline, suggesting that Osr1^+^ cells progress ahead of LECs in the back dermis (Fig. 1E), anticipating LEC migration through the forming dermis. Of note, Osr1^+^ descendants were located in close association with blood and lymphatic vasculature but, in agreement with previous results (Vallecillo-Garcia et al., 2023), mGFP signal was not found in endothelial cells (Fig. S1D).

Analyses of *Osr1* expression in embryonic stages and adult tissues have revealed that *Osr1* expression decreases at late stages of development (Vallecillo-Garcia et al., 2017; Stumm et al., 2018; Kotsaris et al., 2023) and Osr1^+^ cells are found in the stroma of some adult tissues (Vallecillo-Garcia et al., 2023, 2017; Kotsaris et al., 2023). We analyzed Osr1^+^ cell distribution in association with blood and lymphatic vasculature in adult ear dermis and lymph nodes of *Osr1^lacZ/+^* animals. Whole-mount *lacZ* staining revealed that Osr1^+^ cells were associated with arteries in the dermal tissue of the ear and with lymphatic vessels in the medulla of mesenteric lymph nodes of *Osr1^lacZ/+^* animals (Fig. 1F; Fig. S1E). Ear punch trauma led to an activation of *Osr1* expression in surrounding mesenchyme (Fig. 1F, right panel; Fig. S1F) in cells closely associated with lymphatic and blood vasculature (Fig. 1G; Fig. S1G). In summary, Osr1^+^ cells accompany LEC migration during lymphatic vasculature formation in the embryo and persist as mesenchymal vessel-associated cells, including mural cells, in adult tissues.

### Osr1^+^ mesenchymal cells control lymphatic vasculature formation

**Fig. 2.**
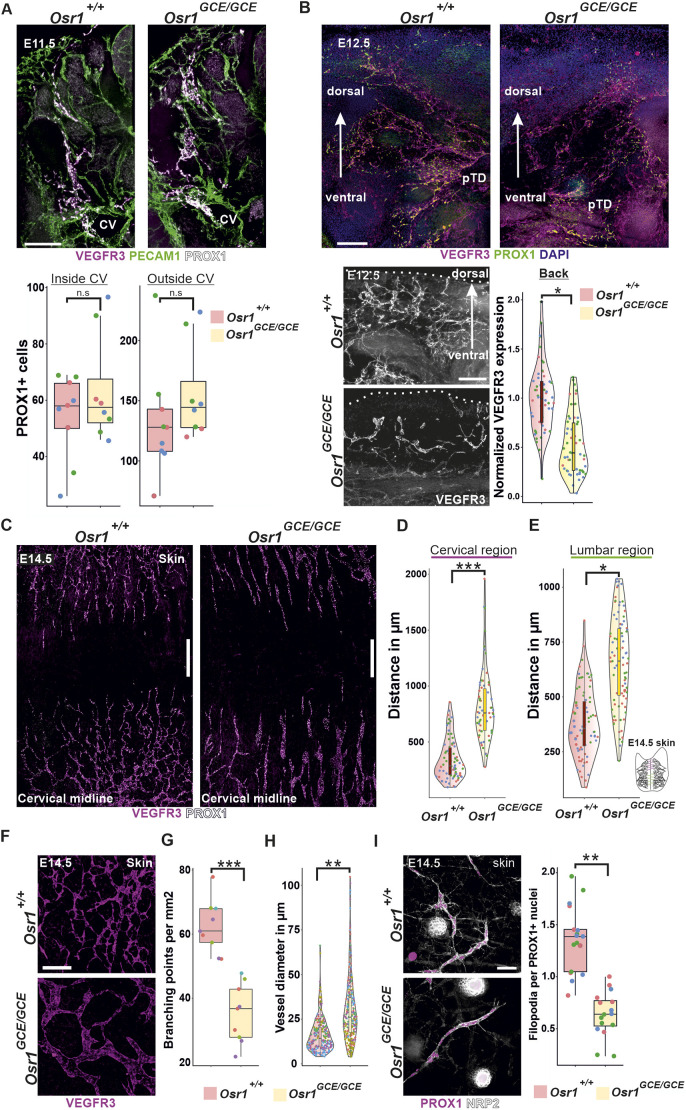
**Lack of Osr1 in mesenchymal cells leads to lymphatic vasculature defects.** (A) Maximal intensity projection images of *Osr1^+/+^* and *Osr1^GCE/GCE^* E11.5 100 µm cross-sections after immunofluorescence. Immunolabeling for PROX1, VEGFR3 and PECAM1 shows normal commitment and delamination of LECs and normal blood endothelial structures in the dorsal part of the cardinal vein. Below, quantification of PROX1^+^ nuclei inside of the cardinal vein and dorsally migrating outside of the cardinal vein. *n*=3. (B) At E12.5, maximal intensity projection images of 100 µm cross-sections reveal first impairments in lymphatic structures labeled for PROX1 and VEGFR3. Below, quantification of VEGFR3^+^ lymphatic vasculature in the most dorsal region of the back. *n*=3. Dotted line represents the edge of the embryo. (C) Whole-mount immunofluorescence of E14.5 *Osr1^+/+^* and *Osr1^GCE/GCE^* skin samples for VEGFR3 and PROX1. (D,E) Quantification of the distance in µm between the tips of the migrating lymphatic vasculature front and the center of the avascular line in the skin in cervical and lumbar regions. *n*=3. (F) Representative micrographs of E14.5 *Osr1^+/+^* and *Osr1^GCE/GCE^* skin labeled for VEGFR3. (G,H) Quantification of lymph vessel branching points per mm^2^ (G, *n*=4) and vessel diameter (H, *n*=5) in E14.5 *Osr1^+/+^* and *Osr1^GCE/GCE^* skin labeled for VEGFR3. (I) Representative images of skin whole-mount immunofluorescence showing reduced filopodia formation at the tips of lymphatic vessels in the back of E14.5 *Osr1^GCE/GCE^* embryos. Filopodia protrusions are immunolabeled with anti-NRP2 and LECs are detected by anti-PROX1. Right, quantification of filopodia protrusions per PROX1^+^ cell. *n*=3. Measurements obtained from the same embryo are represented as dots with the same color. Representative immunofluorescence images have been captured from at least three different embryos. Scale bars: 200 µm (A,F); 100 µm (B); 500 µm (C); 50 µm (I). *P<0.05, **P<0.01, ***P<0.001 (unpaired, two-tailed Student's *t*-tests). n.s., not significant. Box plots show median values (middle bars), first to third interquartile ranges (boxes), whiskers indicate 1.5× the interquartile ranges and dots indicate measured sample values. CV, cardinal vein; pDT, primordial thoracic duct.

As *Osr1*-expressing mesenchymal cells associated with migrating LECs and embedded the first lymphatic vasculature in the skin, we asked whether impairment of the lymphatic vasculature could be responsible for the back edema observed in *Osr1^GCE/GCE^* (knockout; KO) embryos (Fig. S2A; Wang et al., 2005). We first analyzed whether the commitment or delamination of LECs from the cardinal vein are affected in *Osr1^GCE/GCE^* embryos. Immunofluorescence of 100-µm thick sections from E11.5 *Osr1^+/+^* and *Osr1^GCE/GCE^* embryos showed no significant changes in LEC commitment or delamination into the surrounding mesenchyme, together with normal blood vasculature structures (Fig. 2A; Fig. S2B). In line, quantification of PROX1^+^ LECs inside the cardinal vein and migrating into the mesenchyme dorsally from the cardinal vein were not significantly changed in E11.5 Osr1*^GCE/GCE^* embryos (Fig. 2A, lower panel). At E12.5, the first impairments in lymphatic structures were identified by immunofluorescence in *Osr1^GCE/GCE^* embryos, before a back edema was visible. Migration of LECs towards the dorsal region of the embryo was decreased and the PROX1^+^/VEGFR3^+^ vascular network in the primordial thoracic duct region showed reduced vascular branching (Fig. 2B). We found a reduction of VEGFR3^+^ lymphatic vessels after immunofluorescence of 100-µm thick sections at the back of E12.5 *Osr1^GCE/GCE^* embryos (Fig. 2B, lower panel). In line, whole-mount immunofluorescence of isolated back skin from E14.5 embryos showed markedly impaired migration of dermal LECs in the skin of E14.5 *Osr1^GCE/GCE^* embryos, with significantly increased distances between growing fronts in both cervical and lumbar regions compared with *Osr1^+/+^* controls (Fig. 2C-E; Fig. S2C,D). In addition, lymphatic vessels showed a decrease in the number of branching points (Figs 2C,F,G) and increased caliber (Fig. 2F,H) in E14.5 *Osr1^GCE/GCE^* skin. In line with the defects found in LEC migration, LECs in the migrating front of E14.5 *Osr1^GCE/GCE^* dermis presented a reduced number of filopodia per PROX1^+^ LEC (Figs 2I; Fig. S2E) suggesting an impaired ECM-LEC interaction. Of note, in the skin of E14.5 *Osr1^GCE/GCE^* embryos, blood vasculature showed no significant impairment in vessel thickness or branching point numbers (Fig. S2F).

Overall, this shows that the transcription factor *Osr1* expressed in mesenchymal cells is crucial for the establishment of the lymphatic vasculature; the loss of *Osr1* leads to severe impairments in LEC migration and subsequent formation of lymphatic vasculature structures.

### Transcriptome analysis reveals impaired ECM interaction of LECs in E13.5 *Osr1* KO embryos

**Fig. 3.**
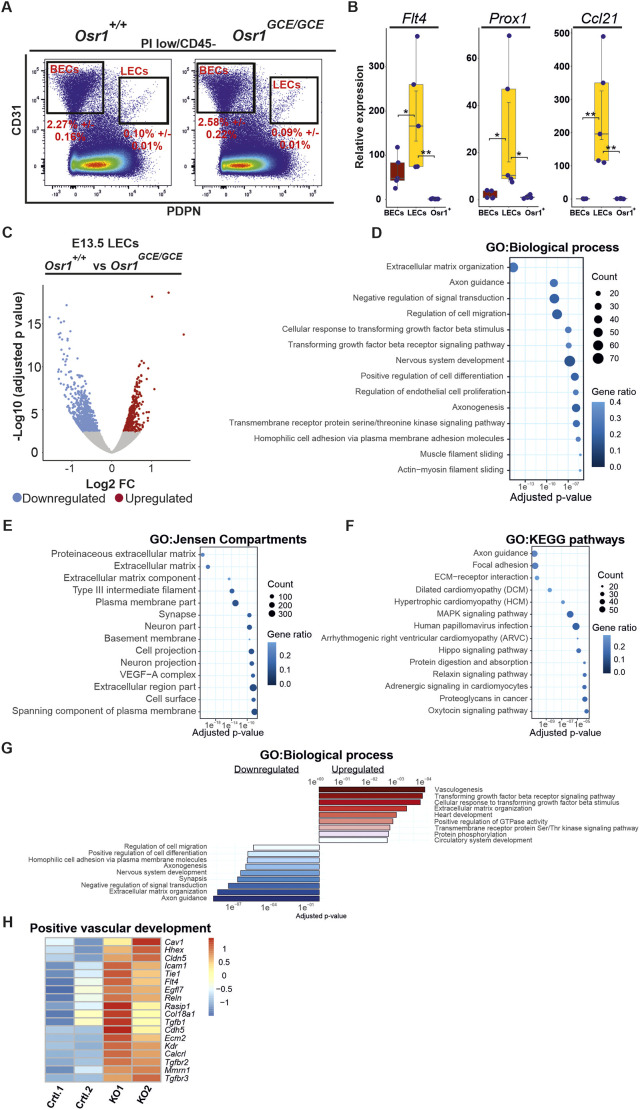
**Transcriptional adaptations of LECs to an environment lacking Osr1.** (A) Cell sorting strategy for isolating E13.5 LECs (CD45^−^ PDPN^+^ CD31^+^) and BECs (CD45^−^ PDPN^−^ CD31^+^) from Osr1^controls^ (*Osr1^+/+^* and *Osr1^GCE/+^*) and *Osr1^GCE/GCE^* embryos. Percentage of cells±standard deviation of the mean is shown. *n*=9. (B) Box plots from RT-qPCR analysis showing relative expression of Vegfr3, Prox1 and Ccl21 in E13.5 FACS isolated BECs, LECs and *Osr1^GCE/+^* cells. Relative expression was normalized to *Osr1^GCE/+^* cells. *n*=5. *P<0.05, **P<0.01 (one-way ANOVA with Dunnett's multiple comparisons). Error bars represent s.e.m. Box plots show median values (middle bars), first to third interquartile ranges (boxes), whiskers indicate 1.5× the interquartile ranges and dots indicate measured sample values. (C) Volcano plot showing transcriptome analysis of E13.5 FACS sorted LECs from *Osr1^controls^* and *Osr1^GCE/GCE^* embryos showing upregulated (red) and downregulated (blue) genes identified by an absolute log2 fold change>0.3 and an adjusted *P*-value<0.05. (D-F) Dot plot depiction of GO analysis for biological processes (D), Jensen Compartments (E) and KEGG pathways (F) using all deregulated genes; top 14 terms ranked by their adjusted *P*-value are shown. Count represents number of genes in the term; gene ratio represents the percentage of significant genes over the total genes in a given term. (G) Bar plot representation of GO analysis for biological processes performed in upregulated (red) or downregulated genes (blue). Terms were ranked by their adjusted *P*-value. (H) Heatmap depiction of TPM values for selected genes positively involved in lymphatic vessel formation. Raw scaled normalization is represented on the right.

At E13.5, *Osr1* is transcribed mainly in mesenchymal cell populations and is not detected in LECs (Vallecillo-Garcia et al., 2023, 2017). To assess LEC defects in E13.5 *Osr1-*deficient embryos, we first isolated E13.5 LECs (PDPN^+^ PECAM1^+^) via fluorescence activated cell sorting (FACS) (Fig. 3A). Enrichment of LECs was confirmed via real-time (RT)-qPCR analysis of key LEC markers *Vegfr3*, *Prox1* and *Ccl21* compared with FACS-isolated E13.5 blood endothelial cells (BECs) and GFP^+^ mesenchymal cells from *Osr1^GCE/+^* embryos (Fig. 3B). Of note, relative percentages of BECs and LECs were not impaired in E13.5 *Osr1^GCE/GCE^* embryos (Fig. S3A), suggesting that there was no general defect in LEC pool expansion and maintenance. Next, we performed transcriptome analysis of E13.5 LECs from *Osr1^controls^* and *Osr1^GCE/GCE^* embryos. Consistent with a high expression of LEC markers observed via RT-qPCR (Fig. 3B), RNA-seq analysis from E13.5 LECs showed higher expression of LEC markers compared with BEC (Jurisic and Detmar, 2009) and mesenchymal markers (Table S1). This analysis revealed 1386 differentially expressed genes (DEGs) with 877 down-, and 509 upregulated genes (Fig. 3C). We further characterized DEGs by performing gene ontology (GO) analysis. GO analysis for biological processes on all DEGs revealed an enrichment of terms associated with ECM organization and cell migration (Fig. 3D; Fig. S3B). ECM-associated terms were also identified by GO analysis using the Jensen compartment database (Fig. 3E), and the term ECM-receptor interaction was enriched after GO analysis using the Kyoto Encyclopedia of Genes and Genomes (KEGG) pathway database (Fig. 3F; Fig. S3C). Further GO analysis for biological processes by separating down- and upregulated genes showed an enrichment of ECM terms in genes downregulated in LECs of *Osr1^GCE/GCE^* embryos (Fig. 3G). In line, GO analysis against the Jensen Compartment database by separating down- and upregulated genes revealed genes associated with ECM-related terms as top ranked within downregulated genes (Fig. S3D). Among genes upregulated in LECs of E13.5 *Osr1^GCE/GCE^* embryos, GO analysis for biological processes revealed terms associated with vascular development ranking top (Fig. 3G). In line with this, selected genes known to have a positive effect in lymphatic vasculature formation (Schulte-Merker et al., 2011; Alitalo et al., 2005; Gauvrit et al., 2018; Liu et al., 2018; Xiang et al., 2020) were found to be upregulated in LECs of E13.5 *Osr1^GCE/GCE^* embryos (Fig. 3H).

In summary, lack of *Osr1* in mesenchymal cells leads to a series of transcriptional adaptations in embryonic LECs. Mesenchymal cell impairment leads to decreased expression of ECM and ECM-receptor interaction genes and downregulation of genes involved in cell migration in LECs of *Osr1^GCE/GCE^* embryos. Finally, E13.5 *Osr1^GCE/GCE^* LECs activate genes positively involved in lymphatic vessel formation, suggesting a compensatory mechanism.

### Transcriptome analysis of mesenchymal Osr1^+^ cell–LEC interactions via the ECM

**Fig. 4.**
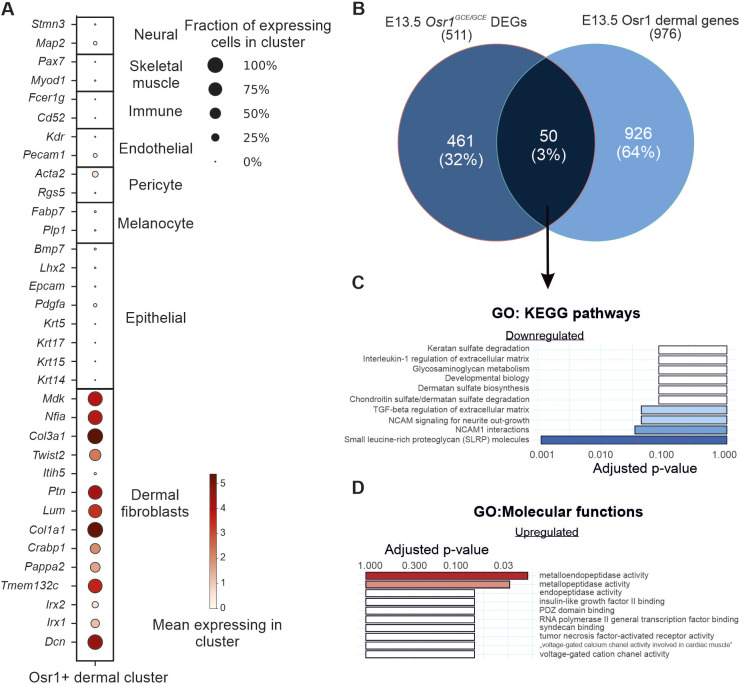
**LECs-Osr1 interactions in the dermis.** (A) Expression of key genes for the main cell populations of embryonic skin tissues (Gupta et al., 2019) in the E13.5 Osr1^+^ dermal cell cluster identified by scRNA-seq (Vallecillo-Garcia et al., 2023). (B) Venn diagram intersecting deregulated genes obtained after RNA-seq analysis of E13.5 *Osr1^GCE/+^* versus *Osr1^GCE/GCE^* cells and genes characterizing the E13.5 Osr1 dermal cluster identified by scRNA-seq analysis. (C,D) Bar plot representation of GO analysis using 50 genes deregulated in *Osr1^GCE/GCE^* cells and enriched in E13.5 dermal cluster for KEGG pathways in downregulated genes (blue) and for molecular functions in upregulated genes (red). Terms were ranked by their adjusted P-value.

RNA-seq analysis revealed that LECs in *Osr1^GCE/GCE^* embryos have a deregulated transcriptional signature of ECM organization and ECM-receptor interaction, with many genes related to these terms being downregulated (Fig. 3; Fig. S3). To address this from the perspective of Osr1^+^ mesenchymal cells, we used our previously published RNA-seq dataset of Osr1^+^ cells from E13.5 *Osr1^GCE/+^* versus *Osr1^GCE/GCE^* embryos (Vallecillo-Garcia et al., 2017). To specifically select for dermal fibroblast-expressed genes, we intersected the 511 E13.5 *Osr1^GCE/GCE^* DEGs with the 976 genes found to be highly abundant in the dermal cluster of E13.5 *Osr1*-expressing cells extracted from a published single-cell (sc)RNA-seq dataset (Vallecillo-Garcia et al., 2023), given that this cluster was enriched for key embryonic skin dermal fibroblast population markers (Ge et al., 2020; Gupta et al., 2019) (Fig. 4A). Among the genes deregulated in E13.5 *Osr1^GCE/GCE^* cells, we identified 50 deregulated ‘dermal’ genes (Fig. 4B; Table S6). We subjected this set of genes to GO analysis and found the terms ‘small leucine-rich proteoglycan (SLRP) molecules’, ‘NCAM1 interactions’ and ‘TGF-beta regulation of extracellular matrix’ as significant terms downregulated in this intersection (Fig. 4C). Further GO analysis of the molecular functions of upregulated genes in this intersection revealed that ‘metalloendopeptidase activity’ and ‘metallopeptidase activity’ were the only significantly enriched terms (Fig. 4D). Consistent with the observations of ECM defects seen in embryos lacking *Osr1* in other tissues (Vallecillo-Garcia et al., 2017; Kotsaris et al., 2023), this analysis suggested that in dermal E13.5 *Osr1*-deficient cells, ECM production and organization mediated by mesenchymal cells may be also impaired.

### Impaired ECM scaffold in the dermis of *Osr1*-deficient embryos

**Fig. 5.**
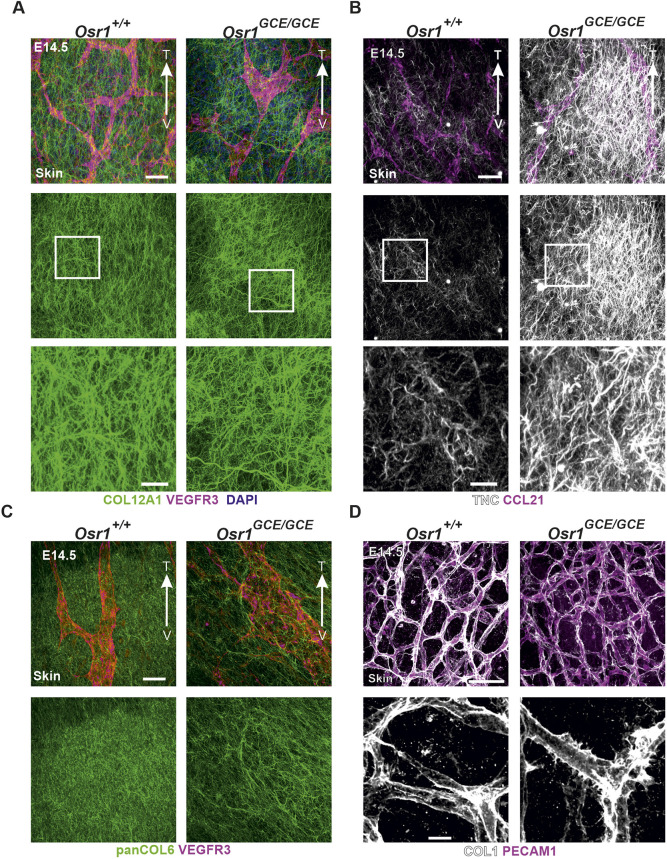
**Impaired dermal ECM in E14.5 *Osr1^GCE/GCE^* embryos*.*** (A-C) Representative micrographs of E14.5 skin whole-mount immunofluorescence showing ECM impairments in *Osr1^GCE/GCE^* embryos. ECM was labeled for COL12A1 (A), TNC (B) and COLVI (C). Lymphatic vasculature is labeled for VEGFR3 (A,C) or CCL21 (B). (D) Representative micrographs of E14.5 skin whole-mount immunofluorescence showing defects in the basal lamina of blood vessels. Endothelial cells are labeled for PECAM1 and basal lamina for COL1. Representative immunofluorescence images have been captured from at least three different embryos. In A-C, arrows show the direction of lymphatic vessels from ventral side (V) to tips (T) pointing to the avascular zone. Scale bars: 50 µm (A-D, upper panels); 20 µm (A,B,D, bottom panels).

Transcriptome analyses of E13.5 Osr1^+^ cells and LECs suggested defective ECM organization in the developing lymph vasculature of *Osr1^GCE/GCE^* embryos. We therefore characterized the ECM in direct contact with the dermal vasculature. Dermal fibroblasts are the primary source of ECM scaffold components such as *Col12a1*, *Col1a1*, *Fn1*, *Col3a1*, or small leucine-rich proteoglycans (SLRPs) such as *Dcn* or *Lum*; of note, *Osr1* expression is enriched in dermal fibroblasts (Rezza et al., 2016) (Fig. S4A). Whole-mount immunofluorescence of E14.5 skin using antibodies against the ECM proteins COL12A1, COL6 and TNC revealed an impaired ECM scaffold in direct contact with lymphatic vasculature showing frayed fiber organization and increased TNC expression in E14.5 *Osr1^GCE/GCE^* embryos (Fig. 5A-C). The representative images shown in Fig. 5A-C display lymphatic vessels and the surrounding ECM of lymphatic vessels at the front of the migrating zone; of note, ECM-organization defects were observed in ventral regions as well as in the region of the dorsal migrating front of LECs (Fig. S4B). Despite normal formation of dermal blood vessels observed in E14.5 *Osr1^GCE/GCE^* embryos (Fig. S2F), closer appreciation of the basal lamina in dermal blood capillaries showed an irregular, ruffled COL1 staining along the PECAM1^+^ endothelial layer (Fig. 5D). In contrast to blood capillaries, lymphatic vessels at this stage showed almost an absent COL1 staining along the VEGFR3^high^ endothelial layer in both controls and *Osr1^GCE/GCE^* embryos (Fig. S4C).

### Mesenchymal Osr1^+^ cells are a source of *Vegfc* and control LEC proliferation via a proactive-ECM

**Fig. 6.**
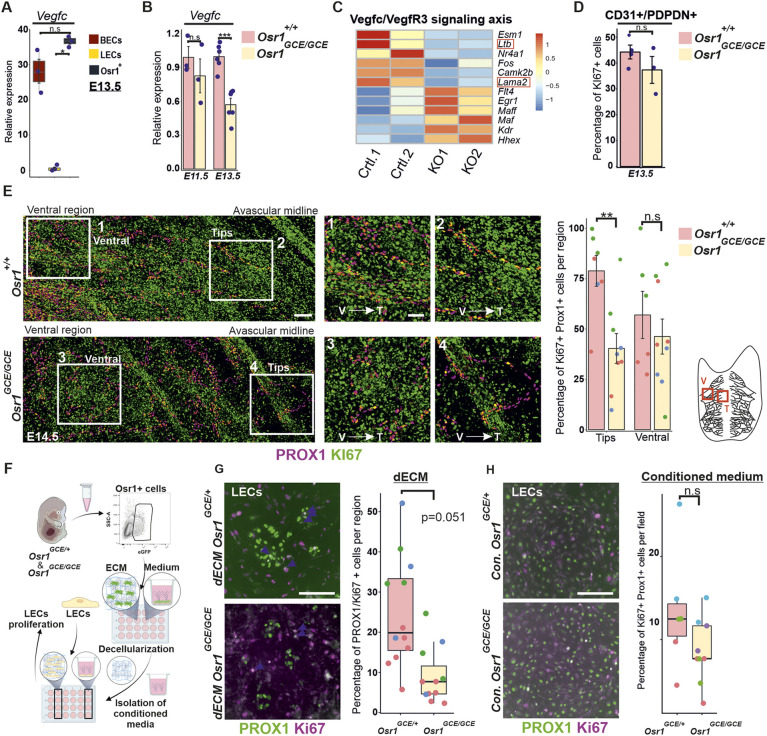
**Mesenchymal Osr1^+^ cells promote LEC proliferation at the migrating front via the ECM.** (A) Relative expression of *Vegfc* in E13.5 *Osr1^GCE/+^* cells, BECs and LECs isolated by FACS. *n*=3. (B) RT-qPCR analysis showing *Vegfc* relative expression in E11.5 (*n*=3) and E13.5 (*n*=6) FACS isolated *Osr1^GCE/+^* and *Osr1^GCE/GCE^* cells. (C) Heatmap depiction of TPM values for selected genes involved in the VEGFC/VEGFR3 signaling pathway. Raw normalized scale is represented on the right. Genes surrounded by a red square are not in agreement with upregulation of the VEGFC/VEGFR3 signaling pathway described by others. (D) Quantification of Ki67^+^/PROX1^+^ cells in cultured FACS-isolated LECs from E13.5 *Osr1^GCE/+^* and *Osr1^GCE/GCE^* embryos shows no differences in proliferation. *n*=3. (E) Representative micrographs of E14.5 skin whole-mount immunofluorescence showing Ki67^+^ proliferative LECs labeled by PROX1. Quantification of PROX1^+^/Ki67^+^ cells in ventral (V) and migration front (T) regions is shown on the right, together with a schematic representation of skin lymphatic vessels and regions measured. *n*=3. (F) Schematic of experimental workflow for decellularized (d)ECM and conditioned media production from E13.5 FACS isolated *Osr1^GCE/+^* and *Osr1^GCE/GCE^* cells. Subsequent tdLEC culture was performed on dECM or using conditioned media. (G) Immunofluorescence of tdLECs cultured on dECM for 48 h. LECs are labeled for PROX1 and Ki67 for proliferation. Quantification of proliferative LECs is shown on the right. *n*=3. (H) Immunofluorescence of tdLECs cultured for 24 h in conditioned media coming from E13.5 FACS isolated *Osr1^GCE/+^* and *Osr1^GCE/GCE^* cells. LECs are labeled for PROX1 and Ki67 for proliferation. Quantification of proliferative LECs is shown on the right**.** Control *n*=3 and KO *n*=4. Measurements obtained from the same embryo are represented as dots with the same color. Representative immunofluorescence images have been captured from at least three different embryos. Scale bars: 100 µm (E); 50 µm (E1-E4); 200 µm (G,H). *P<0.05, **P<0.01, ***P<0.001 (A: one-way ANOVA with Dunnett's multiple comparisons; B, D, E, H: unpaired, two-tailed Student's *t*-test; G: paired, two-tailed Student's *t*-test). n.s., not significant. Error bars represent s.e.m. Box plots show median values (middle bars), first to third interquartile ranges (boxes), whiskers indicate 1.5× the interquartile ranges and dots indicate measured sample values.

Activation of the VEGFC/VEGFR3 signaling pathway is essential for lymphatic vessel formation driving LEC delamination from the cardinal vein and LEC proliferation (Karkkainen et al., 2004; Zhang et al., 2010; Wang et al., 2001; Makinen et al., 2001; Flister et al., 2010). Available scRNA-seq data for the stages E9.5-E13.5 (Cao et al., 2019) showed that mesenchymal cells and endothelial cells were the major source of *Vegfc* expression (Fig. S5A). We confirmed that E13.5 Osr1^+^ cells showed high expression of *Vegfc*, together with endothelial cells, by comparing *Vegfc* transcriptional expression in Osr1^+^ cells, BECs and LECs separated by FACS (Fig. 6A). At E11.5, *Vegfc* expression in BECs was higher compared with mesenchymal Osr1^+^ cells; however, at E13.5 BECs and Osr1^+^ cells express comparable *Vegfc* levels (Fig. S5B). Transcriptome analysis of E13.5 *Osr1^GCE/+^* and E13.5 *Osr1^GCE/GCE^* mesenchymal cells revealed a decrease in *Vegfc* transcripts in cells lacking *Osr1* (Vallecillo-Garcia et al., 2017). We confirmed *Vegfc* transcript downregulation in E13.5 *Osr1^GCE/GCE^* mesenchymal cells, whereas at E11.5, *Osr1^GCE/GCE^* mesenchymal cells produced similar *Vegfc* transcript levels as controls (Fig. 6B). Contradictory to the notion that reduced *Vegfc* in mesenchymal cells may cause a downregulation of *Vegfr3* in LECs, we observed an increase in *Vegfr3* expression in LECs of E13.5 *Osr1^GCE/GCE^* embryos, together with other genes involved in the VEGFR3-signaling axis such as *Hhex*, *Maf* or *Egr1* (Koltowska et al., 2015; Gauvrit et al., 2018; Shin et al., 2008; Dieterich et al., 2017) (Fig. 6C). As BECs are also a source of *Vegfc*, we analyzed *Vegfc* expression in E13.5 *Osr1^GCE/GCE^* embryos via RT-qPCR analysis of FACS-isolated BECs and did not observe a change in *Vegfc* expression (Fig. S5C). Of note, available transcriptome analysis of E14.5 skin populations confirms fibroblasts and melanocytes as the main cell types expressing *Vegfc* (Sennett et al., 2015) (Fig. S5D).

E13.5 LECs isolated by FACS from E13.5 whole *Osr1^+/+^* and *Osr1^GCE/GCE^* embryos and cultured *in vitro* did not show defects in cell proliferation (Fig. 6D). Interestingly, using whole-mount immunofluorescence we detected a heterogeneous proliferation of LECs in wild type embryos, depending on their distribution in the skin. LECs in the migrating front show higher proliferation quantified by PROX1/Ki67 co-staining, compared to LECs on the ventral side (Fig. 6E). Quantification of E14.5 LECs proliferation showed a reduced proliferation in E14.5 *Osr1^GCE/GCE^* embryos specifically in the migrating front, while LECs in the ventral lymphatic vasculature (Fig. 6E) showed similar proliferation in *Osr1*-deficient and control embryos.

In order to untangle the mechanism used by Osr1^+^ mesenchymal cells to control LEC proliferation, we aimed to separate the effects coming from signaling molecules secreted by Osr1^+^ cells and the effects of the ECM scaffold produced by Osr1^+^ cells. For this purpose, we isolated E13.5 *Osr1^GCE/+^* and *Osr1^GCE/GCE^* cells via FACS and let them produce either an ECM scaffold or a conditioned medium (Fig. 6F). Next, we isolated dermal LECs from the tail (tdLECs) of adult wild-type mice (Fig. S5E,F) and quantified the effects of decellularized ECM (dECM) or cell-conditioned media on tdLECs proliferation (Fig. 6F). LECs were cultured for 48 h on dECM produced by Osr1^+^ cells (Fig. S5G). LECs cultured on dECM produced by E13.5 *Osr1^GCE/GCE^* cells showed reduced proliferation, quantified by PROX1^+^/Ki67^+^ co-staining, compared with LECs cultured on dECM produced by E13.5 *Osr1^GCE/+^* cells (Fig. 6G). Conversely, conditioned medium produced by E13.5 *Osr1^GCE/GCE^* cells did not significantly reduce LEC proliferation compared with E13.5 *Osr1^GCE/+^* conditioned medium (Fig. 6H).

We conclude that, although mesenchymal Osr1^+^ cells are an important source of *Vegfc* in the mouse, decreased *Vegfc* in *Osr1^GCE/GCE^* cells did not lead to decreased VEGFR3 downstream target expression in LECs. Instead, a defective ECM secreted by *Osr1^GCE/GCE^* cells affects LEC proliferation *in vitro* in line with decreased LEC proliferation in E14.5 *Osr1^GCE/GCE^* embryos at the migrating front of the growing lymph vasculature.

### Mesenchymal Osr1^+^ cells provide beneficial guidance for LEC migration

**Fig. 7.**
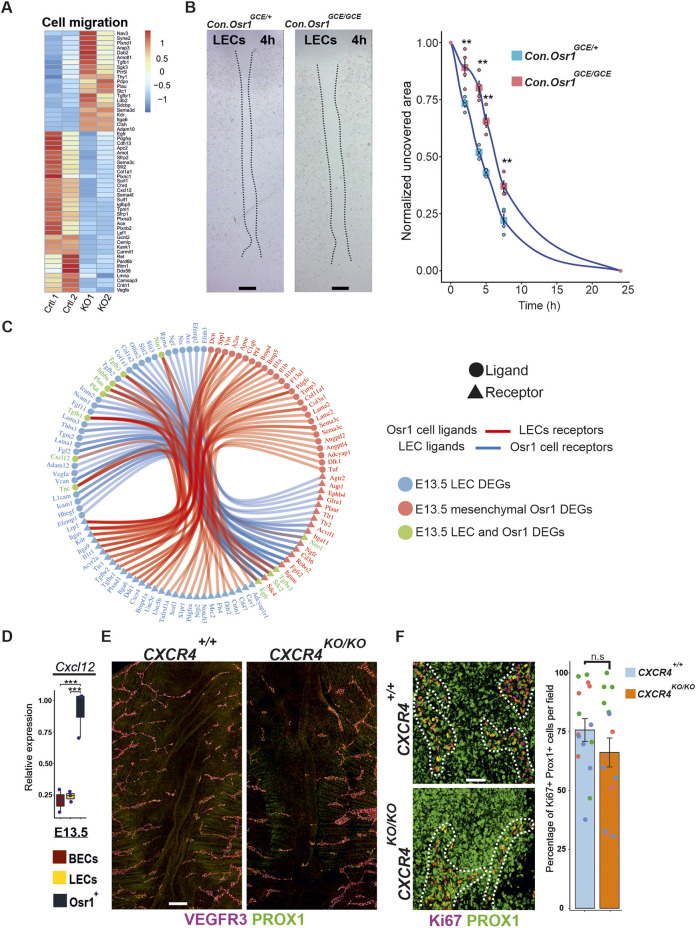
**Mesenchymal Osr1^+^ cells promote LEC migration.** (A) Heatmap depiction of TPM values of genes belonging to the GO biological process ‘regulation of cell migration’. Raw scaled normalization is represented on the right. (B) Migration assay using tdLECs under conditioned media produced by E13.5 *Osr1^GCE/+^* and *Osr1^GCE/GCE^* cells. Representative images after 4 h of culture shown left, quantification of wound closure at 0, 2, 4, 5, 7.5 and 24 h after induction with conditioned media shown on the right. *n*=4. (C) Ligand-receptor pair analysis using DEGs of E13.5 *Osr1^GCE/GCE^* cells and LECs from E13.5 *Osr1^GCE/GCE^* embryos. (D) RT-qPCR analysis showing Cxcl12 relative expression in E13.5 FACS isolated *Osr1^GCE/+^* Osr1^+^ cells, BECs and LECs. *n*=3. (E) E14.5 skin whole-mount immunofluorescence of *Cxcr4^+/+^* and *Cxcr4^KO/KO^* embryos showing lymphatic vessel impairments. Lymphatic vasculature is labeled by VEGFR3. (F) Representative micrographs of E14.5 skin whole-mount immunofluorescence showing normal proliferation of LECs in the migrating front from *Cxcr4^KO/KO^* embryos. LECs are labeled for PROX1 and proliferation measured by Ki67. Quantification of Ki67^+^/PROX1^+^ cells per region is shown on the right. *n*=4. Representative immunofluorescence images have been captured from at least three different embryos. *P<0.05, **P<0.01, ***P<0.001 (D: one-way ANOVA with Dunnett's multiple comparisons; B,F: unpaired, two-tailed Student's *t*-tests). n.s., not significant. Error bars represent s.e.m. Box plot shows median values (middle bars), first to third interquartile ranges (boxes), whiskers indicate 1.5× the interquartile ranges and dots indicate measured sample values. Scale bars: 500 µm (B); 200 µm (E); 50 µm (F).

In line with a reduced LEC migration observed in the skin of E14.5 *Osr1^GCE/GCE^* embryos, cell migration was one of the most enriched terms in the GO analysis for biological processes in E13.5 LEC RNA-seq data, in which the majority of deregulated genes were downregulated (Fig. 7A). We therefore aimed to clarify whether Osr1 controls the expression of signaling molecules in mesenchymal cells that could act on LECs to promote their migration. We assessed LEC migration *in vitro* by performing scratch assays in cultures of tdLECs isolated from the dermis of adult tail tissues supplemented with conditioned media produced by E13.5 *Osr1^GCE/+^* and *Osr1^GCE/GCE^* cells. tdLECs cultured for 24 h in conditioned medium from E13.5 *Osr1^GCE/GCE^* cells migrated slower into the acellular space compared with LECs cultured in conditioned medium from E13.5 *Osr1^GCE/+^* control cells (Fig. 7B). To evaluate putative interactions between E13.5 Osr1^+^ cells and LECs, we analyzed ligand-receptor interactions using all deregulated genes found in E13.5 *Osr1^GCE/GCE^* cells (511 genes) and in E13.5 LECs (1386 genes) that matched with the interacting pairs defined in Skelly et al. (2018) (Fig. 7C; Table S7). Within the interactions of deregulated ligands in Osr1^+^ cells and deregulated LEC receptors, we found that the Osr1-LEC ligand-receptor pairs TNC-EGFR/ITGA9/ITGAV represented interactions of genes found upregulated in both cell types in line with increased TNC protein abundance in *Osr1^GCE/GCE^* skin (Fig. 5B). By contrast, the ligand-receptor pairs COL3A1-DDR1*/*2 and CXCL12-CXCR4 depicted ligands highly expressed by Osr1^+^ cells (*Col3a1* and *Cxcl12*), which were downregulated in E13.5 *Osr1^GCE/GCE^* cells. Conversely, their interaction partners (*Ddr1*/*2* and *Cxcr4*) were downregulated in LECs of *Osr1^GCE/GCE^* embryos. The chemokine CXCL12 controls LEC migration in zebrafish development and in newborn mice via its receptor CXCR4 (Peng et al., 2022; Cha et al., 2012). Therefore, we asked whether CXCL12 might have a similar function in embryonic dermal lymphatic vasculature formation in mouse embryos. First, we assessed *Cxcl12* expression in E13.5 Osr1^+^ cells, BECs and LECs and observed that mesenchymal Osr1^+^ cells appear as the main source of *Cxcl12* expression (Fig. 7D). Next, we used the *Cxcr4^KO/KO^* line (Zou et al., 1998) to assess the importance of the CXCL12/CXCR4 axis and performed whole-mount immunofluorescence for LEC markers. At E14.5 LECs showed impaired migration to the dorsal midline in E14.5 *Cxcr4^KO/KO^* embryos, with a concomitant increase in lymphatic vessel caliber and reduced arborization (Fig. 7E; Fig. S6A), similar to E14.5 *Osr1^GCE/GCE^* embryos. In line with the pro-migratory function assigned to *cxcl12* in zebrafish (Peng et al., 2022), LECs in *Cxcr4^KO/KO^* embryos did not show a reduced proliferation at the tip of the migrating front (Fig. 7F). This suggests that, in the mouse, CXCL12/CXCR4 signaling is required for LEC dorsal migration downstream of mesenchymal *Osr1*.

## DISCUSSION

The formation of lymphatic vasculature during development is crucial for body fluid homeostasis and, therefore, for animal survival. Although several steps in lymphatic vessel development have been well characterized, the role of mesenchymal cells in lymphatic vessel development has remained understudied.

We have shown that mesenchymal Osr1^+^ cells accompany the early migration path of LECs from the cardinal vein to peripheral tissues, including the mesenchyme surrounding the primordial thoracic duct, and Osr1^+^ cells remain in close association with lymphatic vasculature in several vascular beds. Lineage tracing experiments suggest that mesenchymal Osr1^+^ cells appear in the mesenchyme preceding the LEC migration front in the dermis of the embryo. Osr1^+^ descendants also contribute to mural cells of arteries and veins; it remains to be elucidated whether Osr1^+^ cells constitute a population of mural cells for mature lymphatic vessels. Interestingly, in the medulla of lymph nodes, *Osr1* expression remains active in close association to blood and lymphatic vessels, and *Osr1* and *Vegfc/Vegfa* expression are both found in lymph node mesenchymal stromal subpopulations (data not shown, Cyster Lab Shyni Server).

Reminiscent of *Osr1* expression induction in skeletal muscle mesenchymal fibro-adipogenic progenitors triggered by acute muscle injury (Stumm et al., 2018), we observed transcriptional activation of *Osr1* in the dermis of the ear after trauma. This indicates that *Osr1* activation may represent a general mechanism employ by mesenchymal cells as a trauma response, possibly to induce a pro-remodeling phenotype.

Lack of *Osr1* in mesenchymal cells in *Osr1^GCE/GCE^* embryos led to impairment of lymphatic vasculature formation, indicating that Osr1^+^ mesenchymal cells are a new important player controlling LEC behavior in a non-cell-autonomous fashion. In line with the function assigned to mural cells in zebrafish controlling LEC migration and survival (Peng et al., 2022), we observed LEC migration defects in *Osr1*-deficient embryos and a reduced dermal LEC proliferation specifically at the tips of the migrating front. Conversely, blood vessel morphology and pattern were not significantly impaired in *Osr1*-deficient embryos. Of note, the collagen-rich basal lamina of capillaries in the skin of E14.5 *Osr1^GCE/GCE^* embryos shows defects in COL1 abundance, hinting at a possible function of Osr1^+^ cells in blood vessel stability, which remains to be explored. The differential impact of *Osr1* deficiency on blood and lymphatic vasculature formation is in agreement with the earlier formation of the blood vascular plexus in the embryo (Walls et al., 2008) and a later appearance of Osr1^+^ mesenchymal cells in close proximity to blood vasculature (Mugford et al., 2008; Stricker et al., 2006).

We found that Osr1^+^ cells are a source of *Vegfc* in embryonic tissues. Interestingly, *Vegfc* expression at early stages of development was higher in endothelial cells (Cao et al., 2019), whereas at E13.5 Osr1^+^ mesenchymal cells and BECs expressed similar amounts of *Vegfc*. Despite *Vegfc* downregulation observed only in E13.5 *Osr1^GCE/GCE^* mesenchymal cells, the LEC transcriptome data suggested that embryonic E13.5 LECs show increased expression of genes involved in the VEGFC-VEGFR3 cascade, at least when assessed at the transcriptional level. This might represent a compensatory mechanism in line with the transcriptional upregulation of ECM components, ECM-interaction genes and other genes positively involved in vascular development, as observed in GO analysis of E13.5 LECs from *Osr1^GCE/GCE^* embryos. Alternatively, two important further aspects may separately or in combination play a role in this transcriptional upregulation. One aspect is the ECM and its mechanical properties sensed by LECs in *Osr1^GCE/GCE^* embryos. We have shown that Osr1^+^ cells are important producers of ECM proteins and that lack of *Osr1* leads to impaired ECM in muscle connective tissue (Vallecillo-Garcia et al., 2017), and in the skin of E14.5 embryos embedding lymphatic vasculature and capillaries (Fig. 5; Fig. S4). It has been demonstrated that the VEGFR3 tyrosine kinase function is highly dependent on mechanical forces (Galvagni et al., 2010; Planas-Paz et al., 2012; Baeyens et al., 2015), representing a crucial aspect in signaling pathway activation downstream of VEGFR3, even independent of VEGFC action. It was shown that reduced tissue stiffness leads to the upregulation of genes positively implicated in lymphatic vascular formation, e.g. *Vegfr3*, *Itga9* or *Hey1* (Frye et al., 2018), which we found upregulated in LECs from E13.5 *Osr1^GCE/GCE^* embryos. Intriguingly, we have observed reduced tissue stiffness upon loss of *Osr1* in a model of skeletal muscle injury (Kotsaris et al., 2023). Thus, the defective EMC we found in *Osr1^GCE/GCE^* embryos may lead to the transcriptional upregulation of genes involved in lymphangiogenesis in LECs. Another important aspect also coupled to the ECM component is fluid shear stress sensed by LECs. LECs are very sensitive to changes in shear stress and VEGFR3 builds a mechanosensory complex modulating different aspects of LEC behavior (Baeyens et al., 2015; Angeli and Lim, 2023). *Osr1^GCE/GCE^* embryos show malformation in the septum primum (Wang et al., 2005) and we here found lymphatic vasculature defects; both aspects very likely affect the shear stress experienced by LECs in E13.5 *Osr1^GCE/GCE^* embryos. Thus, the combination of changes in fluid shear stress experienced by LECs on the luminal side and an impaired ECM and defective support of signaling molecules at the basal side could, in combination, explain upregulation of genes involved in lymphangiogenesis in LECs of E13.5 *Osr1^GCE/GCE^* embryos. Furthermore, *Vegfc^+/−^* heterozygous embryos showed only very subtle defects in embryonic lymphangiogenesis (Hagerling et al., 2013; Srinivasan et al., 2014). Altogether, this argues against reduced *Vegfc* expression in *Osr1^GCE/GCE^* cells as the primary explanation for the lymphatic vasculature defects observed in *Osr1* KO embryos.

In the embryonic dermis, fibroblasts are the main producers of ECM components and therefore key players in the formation of the ECM scaffold embedding dermal blood and lymphatic vasculature (Sennett et al., 2015). Lack of *Osr1* leads to a severely disorganized ECM scaffold in the dermis of E14.5 embryos, paralleling our previous observations in skeletal muscle (Vallecillo-Garcia et al., 2017). In addition, the most prominent terms after GO analysis of deregulated genes in LECs of E13.5 *Osr1^GCE/GCE^* embryos are related to ECM and ECM-interaction genes, suggesting that LECs themselves react to the altered ECM produced by *Osr1*-deficient mesenchymal cells. Decellularized ECM, but not conditioned medium, produced by E13.5 *Osr1^GCE/GCE^* cells *in vitro* failed to properly sustain LEC proliferation.

Altogether, this suggests that the reduced proliferation of LECs at the tips of the migrating zone in the dorsal skin of *Osr1^GCE/GCE^* embryos was caused by aberrant ECM deposition from *Osr1*-expressing mesenchymal cells rather than by deregulated expression of signaling molecules such as VEGFC. This agrees with previous reports showing that ECM composition and stiffness can modulate BEC (Yeh et al., 2012; Sack et al., 2016; LaValley et al., 2017) and LEC (Frye et al., 2018) behavior.

In addition to producing the bulk of ECM, mesenchymal cells express signaling molecules that control LEC migration (Peng et al., 2022). Conditioned media experiments suggested that Osr1^+^ mesenchymal cells produce signaling molecules necessary for LEC migration, and transcriptome-based Osr1 cell–LEC interaction analysis highlighted the CXCL12/CXCR4 axis as a possible mechanism. Of note, *Cxcl12* expression is directly regulated by *Osr1* and *Cxcl12* is highly expressed by Osr1^+^ cells (Vallecillo-Garcia et al., 2017). In support of this idea, *CXCR4^KO/KO^* embryos displayed similar defects in lymphatic vessel formation as *Osr1^GCE/GCE^* embryos. However, in the dermis of E14.5 *CXCR4^KO/KO^* embryos, LEC proliferation at the tip of the migrating front was not affected. In agreement, *cxcr4* inhibition in zebrafish mainly affected LEC migration and not their proliferation (Peng et al., 2022). This suggests that the CXCL12/CXCR4 signaling pathway may contribute to LEC migration during lymphatics development in the mouse and in zebrafish, whereas LEC proliferation, specifically at the migration front, is primarily controlled by the ECM. It is noteworthy that the defects in arterial orientation and impairments in smooth muscle cell coverage observed in CXCR4 KO mouse embryos (Ara et al., 2005; Li et al., 2013, 2021) could affect the source of CXCL12 signals required for LEC migration, making it difficult to distinguish between primary and secondary effects.

However, the ECM and factors involved in endothelial-ECM interaction also play a role in controlling endothelial migration and stability (Zhang et al., 2021; Levchenko et al., 2003; Aase et al., 2007). Interestingly, *Amot* and genes encoding direct AMOT interactors such as *Kank1*, *Kank3* and *Flnc* (Angeli and Lim, 2023) are downregulated in LECs of E13.5 *Osr1^GCE/GCE^* embryos (Fig. S6B). We also observed a reduced formation of filopodia at the tips of migrating LECs in E14.5 *Osr1^GCE/GCE^* embryos, altogether suggesting a defective LEC-ECM interaction. We therefore cannot exclude that the altered ECM in *Osr1^GCE/GCE^* embryos may also in part contribute to impaired LEC migration.

In summary, our data show that mesenchymal Osr1^+^ cells play a fundamental role in LEC migration, proliferation and lymphatic vasculature assembly. Hereby, *Osr1* is a key player controlling the production of critical ECM scaffold components and signaling ligands in a bimodal manner to create a microenvironment necessary for lymphatic vessel formation. This parallels the mode of action applied by Osr1^+^ cells in the developmental formation and adult regeneration of skeletal muscle (Vallecillo-Garcia et al., 2017; Kotsaris et al., 2023) and the developmental formation of lymph nodes (Vallecillo-Garcia et al., 2023), and thus suggests an overarching mechanism by which mesenchymal cells control organ formation with the transcription factor *Osr1* at a key nexus.

## MATERIALS AND METHODS

### Animals

Mice were maintained in an enclosed, pathogen-free facility, and experiments were performed in accordance with European Union regulations and under permission from the Landesamt für Gesundheit und Soziales (LaGeSo) Berlin, Germany (permission numbers ZH120, G0346/13, G0240/11, G0268-16). Mouse lines have been described previously; *Osr1^GCE^* (Mugford et al., 2008), *R26R^mTmG^* (Muzumdar et al., 2007), *Osr1^lacZ^* (Stumm et al., 2018), *Cxcr4*^+/−^ (Zou et al., 1998).

### Tamoxifen and Progesterone administration for Osr1^+^ cell lineage tracing

As we described previously (Vallecillo-Garcia et al., 2023, 2017), tamoxifen (Sigma Aldrich) was dissolved in a 1:10 ethanol/sunflower oil mixture. For lineage tracing experiments, we bred *R26R^mTmG^*^/mTmG^ females to *Osr1^GCE^*^/+^ males. Pregnant females were injected with 150 µl of a 20 mg ml^−1^ tamoxifen stock. Tissues were collected at E14.5.

### Tissue preparation

Embryonic tissues were fixed in 4% paraformaldehyde (PFA) for 2 h on ice. Tissues were dehydrated in two steps using 15% and 30% (w/v) sucrose (Roth) solutions before O.C.T. (Sakura) cryo-embedding in a chilled ethanol bath. Embryonic tissue was sectioned at 12 or 100 μm thickness.

### Immunolabeling

Cryosections were warmed up for at least 30 min at room temperature (RT). Sections and E14.5 isolate skin tissue were blocked with 5% (v/v) horse serum (Vector Laboratories) in 0.1% (v/v) Triton X-100 (Sigma-Aldrich) PBS for 1 h at RT. Primary antibodies in blocking solution were incubated at 4°C overnight, followed by secondary antibody staining of 1 h at RT. Antibodies used are listed in Tables S2 and S4. Specimens were counterstained with 5 µg µl^−1^ 4′, 6-diamidino-2-phenylindole (DAPI; Invitrogen) and mounted with FluoromountG (SouthernBiotech).

### Cell isolation and flow cytometry

Isolation of E13.5 Osr1^+^ cells has been described before (Vallecillo-Garcia et al., 2023, 2017). Briefly, inner organs, tail, limbs and cranial tissue above the tongue were removed from E13.5 *Osr1^GCE^*^/+^ and *Osr1^GCE^*^/GCE^ embryos. Next, embryonic tissue was minced using a small scissor in 1 ml high-glucose Dulbecco's modified eagle medium (DMEM, Pan Biotech) containing 10% fetal bovine serum (FBS, Pan Biotech) and 1% penicillin/streptomycin (P/S) solution. Further enzymatic digestion was performed using 0.7 mg ml^−1^ of Collagenase (Collagenase A, Roche) in DMEM medium at 37°C for 45 min. For the isolation of E13.5 LECs and BECs, embryonic tissue was dissected from E13.5 *Osr1^controls^* and *Osr1^GCE/GCE^* embryos and treated as described above for E13.5 Osr1^+^ mesenchymal cells. Antibody labeling (for antibodies see Table S3) was performed for 20 min on ice.

Isolation of primary tail-derived LECs (tdLECs) has been described before (Hagerling et al., 2018). Briefly, tails of at least seven wild-type adult animals (8-30 weeks) were used. Tails were cut at the attachment site and washed twice with Hank's Balanced Salt Solution (HBSS) containing 1% P/S. Next, epidermis and dermis were isolated mechanically from the underlying musculoskeletal system. Isolated tissue was cut in pieces of 2 cm and digested for 1 h at 37°C in a HBSS solution containing 1% P/S and 2 U ml^−1^ Dispase II (Roche). The epidermal layer was separated from the dermis using two tweezers. Collected dermal tissues were further digested enzymatically in 30 ml DMEM medium containing 10% FBS, 1% P/S and 1 mg ml^−1^ collagenase A (Roche) for 90 min. Digested tissue was filtered through a 100 µm cell strainer and cells were collected by centrifugation at 300 ***g*** for 10 min. Cell suspensions were cultured on 0.4% gelatine-coated dishes using LEC medium that contained high-glucose DMEM, 20% FBS, 1% P/S solution, 10 µg/ml endothelial cell growth supplement (ECGS) (Thermo Fisher Scientific), 50 µM 2-mercaptoethanol, 1% non-essential amino acids solution (Thermo Fisher Scientific) and 50 µM for 4-7 days. Before FACS purification, cells were detached using Accutase (Thermo Fisher Scientific) for epitope conservation and washed once with HBSS supplemented with 1% P/S and 0.4% FBS. Antibody labeling was performed for 20 min on ice.

Before flow cytometry, cell suspensions were washed using a solution containing PBS, 0.4% FBS and 2 mM EDTA, collected by centrifugation at 300 g for 5 min and passed through a 35-µm cell strainer filter (BD Biosciences). To assess viability, cells were stained with Propidium Iodide (2 μg ml^−1^, eBioscience) immediately before sorting or analysis.

Sorts and analyses were performed on a FACS Aria II and FACS Aria fusion (BD Biosciences). Data were collected using FACSDIVA software. Further analyses were performed using FlowJo 10 (FlowJo LLC) software. Sorting gates were defined based on unstained and fluorescence negative controls. Cells were collected into 400 µl high-glucose DMEM containing 10% FBS, and 1% P/S solution for Osr1^+^ mesenchymal cells or LEC medium.

### Conditioned media and wound healing assay

For the production of conditioned media, 80,000 E13.5 *Osr1^GCE^*^/+^ and *Osr1^GCE^*^/GCE^ cells isolated by FACS were plated in 24-well plates. After 100% confluence was reached, Osr1^+^ cells were cultured in 300 µl high-glucose DMEM containing only 1% P/S solution. After 24 h, media containing secreted molecules from E13.5 *Osr1^GCE^*^/+^ and *Osr1^GCE^*^/GCE^ cells were collected and used as a conditioned medium. Conditioned media from several time points was collected.

For wound healing assays, 24-well plates were coated with 0.4% gelatine and 20,000 tdLECs (P3-P5) were seeded into two-wells culture-inserts (Ibidi^®^). After 45 min two-wells culture-inserts were removed and gap closure was monitored every 2 h using a Leica DMi8 microscope.

### ECM deposition and decellularization

For ECM deposition, 80,000 embryonic E13.5 *Osr1^GCE^*^/+^ and *Osr1^GCE^*^/GCE^ cells isolated by FACS were plated on a 10 mm coverslip coated with 1 mg/ml fibronectin and cultured in DMEM containing 10% FBS and 1% P/S solution until 100% confluence. Next, cells were cultured for 3 weeks in DMEM containing 10% FBS, 1% P/S solution and 2 µ/ml ascorbic acid. ECM produced by Osr1^+^ cells was decellularized using a freeze/thaw method as described previously (Vallecillo-Garcia et al., 2017). Briefly, culture medium was aspirated and cultures were washed with PBS. Next, PBS was removed and exchanged with 300 µl distilled water. Cultures were frozen in a −80°C freezer and thawed in a 37°C water bath. Remaining water was careful aspirated by pipetting and tdLECs were seeded on the dECM. To assess tdLECs proliferation cultures on dECM, they were cultured for 48 h in LEC medium.

### Imaging

X-Gal staining of whole lymph nodes or the dermis of the ear was documented using a Zeiss SteREO Discovery V12 stereomicroscope. Confocal images of immunolabeled sections were taken using the confocal laser scanning microscope systems LSM710, LSM810 (Zeiss) or Leica DMi8 microscopes. Images were captured using Zen 2010 (Zeiss) and LAS Life System (Leica). For quantifications shown in Fig. 2A, at least three consecutive 100 µm sections were quantified per embryo. In Fig. 2B, four to five regions along the rostro-caudal axis of the back from four to five consecutive 100 µm sagittal sections were quantified per embryo. VEGFR3 expression was quantified using the ImageJ algorithm ‘integrated density’ as the sum of pixels per region of VEGFR3 staining. For normalization, each value was normalized to the average value obtained from wild-type sections. In Fig. 2D,E, the lumbar and cervical regions were quantified separately, assessing the distance of the tips to the center of the avascular midline in at least 20-30 lymphatic vessels per embryo. In Fig. 2G,H and Fig. S2F, branching points, vessel thickness and vessel density were quantified in three different regions of 1 mm^2^ skin per embryo. In Fig. 2I, the number of filopodia per PROX1^+^ LEC was quantified in six different regions at the tip of the migrating zone per embryo. In Figs 6E and 7F, PROX1^+^/KI67^+^ double positive cells were quantified in three different regions at the tip and three different regions at the ventral side per embryo. In Fig. 6G,H, Prox1^High^ cells were quantified per region, choosing three to four regions of 1 mm^2^ per biological replicate (cells or medium derived from one embryo). In each region 20-500 cells per region were quantified. In Fig. 2A,B,D,E,G-I, Fig. S2F, Fig. 6E,G,H and Fig. 7F, measurements from a single embryo are depicted in the same color.

### Quantitative real-time PCR

Total RNA extraction from FACS isolated cells was performed using Direct-zol^TM^ RNA MicroPrep (Zymo Research) following the manufacturer's protocol. Reverse transcription was conducted using the M MuLV Reverse Transcriptase Kit (Biozym). Relative gene expression analyses were performed using GoTaq^®^ qPCR kit (Promega) or Blue S'Green qPCR kit (Byozim) on a 7900HT Real Time PCR system or QuantStudio 7 Flex Real-Time-PCR-System (Applied Biosystems). Primer sequence information is provided in Table S5. Data were acquired and analyzed using SDS 2.0 and QuantStudio^TM^ Real-Time PCR software (Applied Biosystems).

### Transcriptome analysis

To obtain the total RNA amount necessary for RNA-seq, we pooled LECs from eight to ten E13.5 *Osr1^controls^* embryos and eight to ten *Osr1^GCE/GCE^* embryos per sample. E13.5 LECs were isolated by FACS as described above. Total RNA was isolated using Direct-zol RNA Microprep (ZYMO RESEARCH) following the manufacturer's protocol. Total RNA was measured using a Qubit 4 Fluorometer and RNA quality was assessed using an Agilent RNA 6000 Nano kit before library preparation. Library preparation was conducted according to Illumina instructions TruSeq Library Preparation Kit V2. Next, libraries were subjected to high-throughput sequencing using an HiSeq 2500 device. Obtained fastq data were further analyzed using the platform Galaxy Europe (https://usegalaxy.eu). We obtained and mapped 53-81 million reads using STAR (Dobin et al., 2013) against the genome of *Mus musculus* version mm10. Quantification of aligned reads at the gene level was conducted using featureCounts (Liao et al., 2014). Differential gene expression analysis was performed using DESeq2 (Love et al., 2014). Transcript per million (TPM) abundances were calculated using normalized gene counts from DESeq2 analysis of E13.5 *Osr1^GCE/GCE^* LEC samples. Genes with an absolute log2 fold change of ≥0.3, a Benjamini-Hochberg adjusted *P*-value (padj)<0.05, and TPM value>1 were considered as being differentially expressed between E13.5 *Osr1^cantrols^* and *Osr1^GCE/GCE^* LECs. GO analysis was performed using Enrichr (Chen et al., 2013). The ligand-receptor interaction network was drawn using graph-tool v2.45_5 (https://graph-tool.skewed.de/) with a hierarchical edge bundling. Raw fastq and count data were uploaded to the Gene Expression Omnibus (GEO) database under the accession number GSE269397.

### Statistical analysis

Unpaired, two-tailed Student's *t*-test and one-way ANOVA with Dunnett's post-hoc comparison were performed using Prism 8 (GraphPad) software. Error bars in all figures, including supplementary information, represent the mean±standard error of the mean (s.e.m.). In Fig. 6G, a paired, two-tailed Student's *t*-test was used due to the differential proliferation showed by tdLECs isolated from different experiments.

## Supplementary Material



10.1242/develop.202747_sup1 Supplementary information

Table S6. Dermal fibroblast intersection analysis. List of genes found upregulated in E13.5 *Osr1^GCE/+^* cluster 7 (log2FC ≥ 0.2 and percFC ≥ and deregulated in E13.5 *Osr1^GCE/GCE^* mesenchymal cells (log2FC ≥ 0.2 and padj ≤ 0.1).

Table S7. Mesenchymal-LEC ligand-receptor pair analysis. List of ligand-receptor pairs found after analysis of deregulated genes in E13.5 *Osr1^GCE/GCE^* mesenchymal cells (511 genes) and in E13.5 LECs (1386 genes) from *Osr1^controls^* and *Osr1^GCE/GCE^* embryos. For each ligand-receptor pair, the role as a receptor or ligand and the expression (upregulated or downregulated) in E13.5 LECs and mesenchymal cells are defined.

## References

[DEV202747C1] Aase, K., Ernkvist, M., Ebarasi, L., Jakobsson, L., Majumdar, A., Yi, C., Birot, O., Ming, Y., Kvanta, A., Edholm, D. et al. (2007). Angiomotin regulates endothelial cell migration during embryonic angiogenesis. *Genes Dev.* 21, 2055-2068. 10.1101/gad.432007 17699752 PMC1948860

[DEV202747C2] Alitalo, K., Tammela, T. and Petrova, T. V. (2005). Lymphangiogenesis in development and human disease. *Nature* 438, 946-953. 10.1038/nature04480 16355212

[DEV202747C3] Angeli, V. and Lim, H. Y. (2023). Biomechanical control of lymphatic vessel physiology and functions. *Cell. Mol. Immunol.* 20, 1051-1062. 10.1038/s41423-023-01042-9 37264249 PMC10469203

[DEV202747C4] Ara, T., Tokoyoda, K., Okamoto, R., Koni, P. A. and Nagasawa, T. (2005). The role of CXCL12 in the organ-specific process of artery formation. *Blood* 105, 3155-3161. 10.1182/blood-2004-07-2563 15626744

[DEV202747C5] Baeyens, N., Nicoli, S., Coon, B. G., Ross, T. D., Van den Dries, K., Han, J., Lauridsen, H. M., Mejean, C. O., Eichmann, A., Thomas, J. L., et al. (2015). Vascular remodeling is governed by a VEGFR3-dependent fluid shear stress set point. *eLife* 4, e04645. 10.7554/eLife.04645 25643397 PMC4337723

[DEV202747C6] Bussmann, J., Bos, F. L., Urasaki, A., Kawakami, K., Duckers, H. J. and Schulte-Merker, S. (2010). Arteries provide essential guidance cues for lymphatic endothelial cells in the zebrafish trunk. *Development* 137, 2653-2657. 10.1242/dev.048207 20610484

[DEV202747C7] Cao, J., Spielmann, M., Qiu, X., Huang, X., Ibrahim, D. M., Hill, A. J., Zhang, F., Mundlos, S., Christiansen, L., Steemers, F. J. et al. (2019). The single-cell transcriptional landscape of mammalian organogenesis. *Nature* 566, 496-502. 10.1038/s41586-019-0969-x 30787437 PMC6434952

[DEV202747C8] Cha, Y. R., Fujita, M., Butler, M., Isogai, S., Kochhan, E., Siekmann, A. F. and Weinstein, B. M. (2012). Chemokine signaling directs trunk lymphatic network formation along the preexisting blood vasculature. *Dev. Cell* 22, 824-836. 10.1016/j.devcel.2012.01.011 22516200 PMC4182014

[DEV202747C9] Chen, E. Y., Tan, C. M., Kou, Y., Duan, Q., Wang, Z., Meirelles, G. V., Clark, N. R. and Ma'ayan, A. (2013). Enrichr: interactive and collaborative HTML5 gene list enrichment analysis tool. *BMC Bioinformatics* 14, 128. 10.1186/1471-2105-14-128 23586463 PMC3637064

[DEV202747C10] Dieterich, L. C., Ducoli, L., Shin, J. W. and Detmar, M. (2017). Distinct transcriptional responses of lymphatic endothelial cells to VEGFR-3 and VEGFR-2 stimulation. *Sci. Data* 4, 170106. 10.1038/sdata.2017.106 28850122 PMC5574372

[DEV202747C11] Dobin, A., Davis, C. A., Schlesinger, F., Drenkow, J., Zaleski, C., Jha, S., Batut, P., Chaisson, M. and Gingeras, T. R. (2013). STAR: ultrafast universal RNA-seq aligner. *Bioinformatics* 29, 15-21. 10.1093/bioinformatics/bts635 23104886 PMC3530905

[DEV202747C12] Flister, M. J., Wilber, A., Hall, K. L., Iwata, C., Miyazono, K., Nisato, R. E., Pepper, M. S., Zawieja, D. C. and Ran, S. (2010). Inflammation induces lymphangiogenesis through up-regulation of VEGFR-3 mediated by NF-kappaB and Prox1. *Blood* 115, 418-429. 10.1182/blood-2008-12-196840 19901262 PMC2808162

[DEV202747C13] Francois, M., Caprini, A., Hosking, B., Orsenigo, F., Wilhelm, D., Browne, C., Paavonen, K., Karnezis, T., Shayan, R., Downes, M. et al. (2008). Sox18 induces development of the lymphatic vasculature in mice. *Nature* 456, 643-647. 10.1038/nature07391 18931657

[DEV202747C14] Frye, M., Taddei, A., Dierkes, C., Martinez-Corral, I., Fielden, M., Ortsater, H., Kazenwadel, J., Calado, D. P., Ostergaard, P., Salminen, M. et al. (2018). Matrix stiffness controls lymphatic vessel formation through regulation of a GATA2-dependent transcriptional program. *Nat. Commun.* 9, 1511. 10.1038/s41467-018-03959-6 29666442 PMC5904183

[DEV202747C15] Galvagni, F., Pennacchini, S., Salameh, A., Rocchigiani, M., Neri, F., Orlandini, M., Petraglia, F., Gotta, S., Sardone, G. L., Matteucci, G. et al. (2010). Endothelial cell adhesion to the extracellular matrix induces c-Src-dependent VEGFR-3 phosphorylation without the activation of the receptor intrinsic kinase activity. *Circ. Res.* 106, 1839-1848. 10.1161/CIRCRESAHA.109.206326 20431062

[DEV202747C16] Gauvrit, S., Villasenor, A., Strilic, B., Kitchen, P., Collins, M. M., Marin-Juez, R., Guenther, S., Maischein, H.-M., Fukuda, N., Canham, M. A. et al. (2018). HHEX is a transcriptional regulator of the VEGFC/FLT4/PROX1 signaling axis during vascular development. *Nat. Commun.* 9, 2704. 10.1038/s41467-018-05039-1 30006544 PMC6045644

[DEV202747C17] Ge, W., Tan, S. J., Wang, S. H., Li, L., Sun, X. F., Shen, W. and Wang, X. (2020). Single-cell transcriptome profiling reveals dermal and epithelial cell fate decisions during embryonic hair follicle development. *Theranostics* 10, 7581-7598. 10.7150/thno.44306 32685006 PMC7359078

[DEV202747C18] Gupta, K., Levinsohn, J., Linderman, G., Chen, D., Sun, T. Y., Dong, D., Taketo, M. M., Bosenberg, M., Kluger, Y., Choate, K. et al. (2019). Single-cell analysis reveals a hair follicle dermal niche molecular differentiation trajectory that begins prior to morphogenesis. *Dev. Cell* 48, 17-31.e6. 10.1016/j.devcel.2018.11.032 30595533 PMC6361530

[DEV202747C19] Hagerling, R., Pollmann, C., Andreas, M., Schmidt, C., Nurmi, H., Adams, R. H., Alitalo, K., Andresen, V., Schulte-Merker, S. and Kiefer, F. (2013). A novel multistep mechanism for initial lymphangiogenesis in mouse embryos based on ultramicroscopy. *EMBO J.* 32, 629-644. 10.1038/emboj.2012.340 23299940 PMC3590982

[DEV202747C20] Hagerling, R., Hoppe, E., Dierkes, C., Stehling, M., Makinen, T., Butz, S., Vestweber, D. and Kiefer, F. (2018). Distinct roles of VE-cadherin for development and maintenance of specific lymph vessel beds. *EMBO J.* 37, e98271. 10.15252/embj.201798271 30297530 PMC6236332

[DEV202747C21] Jafree, D. J., Long, D. A., Scambler, P. J. and Ruhrberg, C. (2021). Mechanisms and cell lineages in lymphatic vascular development. *Angiogenesis* 24, 271-288. 10.1007/s10456-021-09784-8 33825109 PMC8205918

[DEV202747C22] Janssen, L., Dupont, L., Bekhouche, M., Noel, A., Leduc, C., Voz, M., Peers, B., Cataldo, D., Apte, S. S., Dubail, J. et al. (2016). ADAMTS3 activity is mandatory for embryonic lymphangiogenesis and regulates placental angiogenesis. *Angiogenesis* 19, 53-65. 10.1007/s10456-015-9488-z 26446156 PMC4700087

[DEV202747C23] Jeltsch, M., Jha, S. K., Tvorogov, D., Anisimov, A., Leppanen, V. M., Holopainen, T., Kivelä, R., Ortega, S., Kärpanen, T. and Alitalo, K. (2014). CCBE1 enhances lymphangiogenesis via A disintegrin and metalloprotease with thrombospondin motifs-3-mediated vascular endothelial growth factor-C activation. *Circulation* 129, 1962-1971. 10.1161/CIRCULATIONAHA.113.002779 24552833

[DEV202747C24] Jurisic, G. and Detmar, M. (2009). Lymphatic endothelium in health and disease. *Cell Tissue Res.* 335, 97-108. 10.1007/s00441-008-0644-2 18648856

[DEV202747C25] Karkkainen, M. J., Haiko, P., Sainio, K., Partanen, J., Taipale, J., Petrova, T. V., Jeltsch, M., Jackson, D. G., Talikka, M., Rauvala, H. et al. (2004). Vascular endothelial growth factor C is required for sprouting of the first lymphatic vessels from embryonic veins. *Nat. Immunol.* 5, 74-80. 10.1038/ni1013 14634646

[DEV202747C26] Koltowska, K., Paterson, S., Bower, N. I., Baillie, G. J., Lagendijk, A. K., Astin, J. W., Chen, H., Francois, M., Crosier, P. S., Taft, R. J. et al. (2015). mafba is a downstream transcriptional effector of Vegfc signaling essential for embryonic lymphangiogenesis in zebrafish. *Genes Dev.* 29, 1618-1630. 10.1101/gad.263210.115 26253536 PMC4536310

[DEV202747C27] Kotsaris, G., Qazi, T. H., Bucher, C. H., Zahid, H., Pohle-Kronawitter, S., Ugorets, V., Jarassier, W., Börno, S., Timmermann, B., Giesecke-Thiel, C. et al. (2023). Odd skipped-related 1 controls the pro-regenerative response of fibro-adipogenic progenitors. *NPJ Regen. Med.* 8, 19. 10.1038/s41536-023-00291-6 37019910 PMC10076435

[DEV202747C28] LaValley, D. J., Zanotelli, M. R., Bordeleau, F., Wang, W., Schwager, S. C. and Reinhart-King, C. A. (2017). Matrix stiffness enhances VEGFR-2 internalization, signaling, and proliferation in endothelial cells. *Converg. Sci. Phys. Oncol.* 3, 044001. 10.1088/2057-1739/aa9263 29531793 PMC5844494

[DEV202747C29] Levchenko, T., Aase, K., Troyanovsky, B., Bratt, A. and Holmgren, L. (2003). Loss of responsiveness to chemotactic factors by deletion of the C-terminal protein interaction site of angiomotin. *J. Cell Sci.* 116, 3803-3810. 10.1242/jcs.00694 12902404

[DEV202747C30] Li, W., Kohara, H., Uchida, Y., James, J. M., Soneji, K., Cronshaw, D. G., Zou, Y.-R., Nagasawa, T. and Mukouyama, Y.-R. (2013). Peripheral nerve-derived CXCL12 and VEGF-A regulate the patterning of arterial vessel branching in developing limb skin. *Dev. Cell* 24, 359-371. 10.1016/j.devcel.2013.01.009 23395391 PMC3591512

[DEV202747C31] Li, W., Liu, C., Burns, N., Hayashi, J., Yoshida, A., Sajja, A., González-Hernández, S., Gao, J.-L., Murphy, P. M., Kubota, Y. et al. (2021). Alterations in the spatiotemporal expression of the chemokine receptor CXCR4 in endothelial cells cause failure of hierarchical vascular branching. *Dev. Biol.* 477, 70-84. 10.1016/j.ydbio.2021.05.008 34015362 PMC8277738

[DEV202747C32] Liao, Y., Smyth, G. K. and Shi, W. (2014). featureCounts: an efficient general purpose program for assigning sequence reads to genomic features. *Bioinformatics* 30, 923-930. 10.1093/bioinformatics/btt656 24227677

[DEV202747C33] Lioux, G., Liu, X., Temino, S., Oxendine, M., Ayala, E., Ortega, S., Kelly, R. G., Oliver, G. and Torres, M. (2020). A second heart field-derived vasculogenic niche contributes to cardiac lymphatics. *Dev. Cell* 52, 350-63.e6. 10.1016/j.devcel.2019.12.006 31928974 PMC7374559

[DEV202747C34] Liu, X., Gu, X., Ma, W., Oxendine, M., Gil, H. J., Davis, G. E., Cleaver, O. and Oliver, G. (2018). Rasip1 controls lymphatic vessel lumen maintenance by regulating endothelial cell junctions. *Development* 145, dev165092. 10.1242/dev.165092 30042182 PMC6141773

[DEV202747C35] Love, M. I., Huber, W. and Anders, S. (2014). Moderated estimation of fold change and dispersion for RNA-seq data with DESeq2. *Genome Biol.* 15, 550. 10.1186/s13059-014-0550-8 25516281 PMC4302049

[DEV202747C36] Makinen, T., Veikkola, T., Mustjoki, S., Karpanen, T., Catimel, B. and Nice, E. C. (2001). Isolated lymphatic endothelial cells transduce growth, survival and migratory signals via the VEGF-C/D receptor VEGFR-3. *EMBO J.* 20, 4762-4773. 10.1093/emboj/20.17.4762 11532940 PMC125596

[DEV202747C37] Mugford, J. W., Sipila, P., McMahon, J. A. and McMahon, A. P. (2008). Osr1 expression demarcates a multi-potent population of intermediate mesoderm that undergoes progressive restriction to an Osr1-dependent nephron progenitor compartment within the mammalian kidney. *Dev. Biol.* 324, 88-98. 10.1016/j.ydbio.2008.09.010 18835385 PMC2642884

[DEV202747C38] Muzumdar, M. D., Tasic, B., Miyamichi, K., Li, L. and Luo, L. (2007). A global double-fluorescent Cre reporter mouse. *Genesis* 45, 593-605. 10.1002/dvg.20335 17868096

[DEV202747C39] Niimi, K., Kohara, M., Sedoh, E., Fukumoto, M., Shibata, S., Sawano, T., Tashiro, F., Miyazaki, S., Kubota, Y., Miyazaki, J. et al. (2020). FOXO1 regulates developmental lymphangiogenesis by upregulating CXCR4 in the mouse-tail dermis. *Development* 147, dev181545. 10.1242/dev.181545 31852686

[DEV202747C40] Oliver, G. (2004). Lymphatic vasculature development. *Nat. Rev. Immunol.* 4, 35-45. 10.1038/nri1258 14704766

[DEV202747C41] Peng, D., Ando, K., Hussmann, M., Gloger, M., Skoczylas, R., Mochizuki, N., Betsholtz, C., Fukuhara, S., Schulte-Merker, S., Lawson, N. D. et al. (2022). Proper migration of lymphatic endothelial cells requires survival and guidance cues from arterial mural cells. *eLife* 11, e74094. 10.7554/eLife.74094 35316177 PMC9042226

[DEV202747C42] Pichol-Thievend, C., Betterman, K. L., Liu, X., Ma, W., Skoczylas, R., Lesieur, E., Bos, F. L., Schulte, D., Schulte-Merker, S., Hogan, B. M. et al. (2018). A blood capillary plexus-derived population of progenitor cells contributes to genesis of the dermal lymphatic vasculature during embryonic development. *Development* 145, dev160184. 10.1242/dev.160184 29773646 PMC6001371

[DEV202747C43] Planas-Paz, L., Strilic, B., Goedecke, A., Breier, G., Fassler, R. and Lammert, E. (2012). Mechanoinduction of lymph vessel expansion. *EMBO J.* 31, 788-804. 10.1038/emboj.2011.456 22157817 PMC3280555

[DEV202747C44] Rezza, A., Wang, Z., Sennett, R., Qiao, W., Wang, D., Heitman, N., Mok, K. W., Clavel, C., Yi, R., Zandstra, P. et al. (2016). Signaling networks among stem cell precursors, transit-amplifying progenitors, and their niche in developing hair follicles. *Cell Rep* 14, 3001-3018. 10.1016/j.celrep.2016.02.078 27009580 PMC4826467

[DEV202747C45] Sack, K. D., Teran, M. and Nugent, M. A. (2016). Extracellular matrix stiffness controls VEGF signaling and processing in endothelial cells. *J. Cell. Physiol.* 231, 2026-2039. 10.1002/jcp.25312 26773314

[DEV202747C46] Schulte-Merker, S., Sabine, A. and Petrova, T. V. (2011). Lymphatic vascular morphogenesis in development, physiology, and disease. *J. Cell Biol.* 193, 607-618. 10.1083/jcb.201012094 21576390 PMC3166860

[DEV202747C47] Sennett, R., Wang, Z., Rezza, A., Grisanti, L., Roitershtein, N., Sicchio, C., Mok, K. W., Heitman, N. J., Clavel, C., Ma'ayan, A. et al. (2015). An integrated transcriptome atlas of embryonic hair follicle progenitors, their niche, and the developing skin. *Dev. Cell* 34, 577-591. 10.1016/j.devcel.2015.06.023 26256211 PMC4573840

[DEV202747C48] Shiiya, T. and Hirashima, M. (2023). From lymphatic endothelial cell migration to formation of tubular lymphatic vascular network. *Front. Physiol.* 14, 1124696. 10.3389/fphys.2023.1124696 36895637 PMC9989012

[DEV202747C49] Shin, J. W., Huggenberger, R. and Detmar, M. (2008). Transcriptional profiling of VEGF-A and VEGF-C target genes in lymphatic endothelium reveals endothelial-specific molecule-1 as a novel mediator of lymphangiogenesis. *Blood* 112, 2318-2326. 10.1182/blood-2008-05-156331 18614759 PMC2532805

[DEV202747C50] Skelly, D. A., Squiers, G. T., McLellan, M. A., Bolisetty, M. T., Robson, P., Rosenthal, N. A. and Pinto, A. R. (2018). Single-cell transcriptional profiling reveals cellular diversity and intercommunication in the mouse heart. *Cell Rep.* 22, 600-610. 10.1016/j.celrep.2017.12.072 29346760

[DEV202747C51] Srinivasan, R. S., Escobedo, N., Yang, Y., Interiano, A., Dillard, M. E., Finkelstein, D., Mukatira, S., Gil, H. J., Nurmi, H., Alitalo, K. et al. (2014). The Prox1-Vegfr3 feedback loop maintains the identity and the number of lymphatic endothelial cell progenitors. *Genes Dev.* 28, 2175-2187. 10.1101/gad.216226.113 25274728 PMC4180978

[DEV202747C52] Stanczuk, L., Martinez-Corral, I., Ulvmar, M. H., Zhang, Y., Lavina, B., Fruttiger, M., Adams, R. H., Saur, D., Betsholtz, C., Ortega, S. et al. (2015). cKit lineage hemogenic endothelium-derived cells contribute to mesenteric lymphatic vessels. *Cell Rep.* 10, 1708-1721. 10.1016/j.celrep.2015.02.026 25772358

[DEV202747C53] Stricker, S., Brieske, N., Haupt, J. and Mundlos, S. (2006). Comparative expression pattern of Odd-skipped related genes Osr1 and Osr2 in chick embryonic development. *Gene Expr. Patterns* 6, 826-834. 10.1016/j.modgep.2006.02.003 16554187

[DEV202747C54] Stumm, J., Vallecillo-Garcia, P., Vom Hofe-Schneider, S., Ollitrault, D., Schrewe, H., Economides, A. N., Marazzi, G., Sassoon, D. A. and Stricker, S. (2018). Odd skipped-related 1 (Osr1) identifies muscle-interstitial fibro-adipogenic progenitors (FAPs) activated by acute injury. *Stem Cell Res.* 32, 8-16. 10.1016/j.scr.2018.08.010 30149291

[DEV202747C55] Tammela, T. and Alitalo, K. (2010). Lymphangiogenesis: molecular mechanisms and future promise. *Cell* 140, 460-476. 10.1016/j.cell.2010.01.045 20178740

[DEV202747C56] Uchida, Y., James, J. M., Suto, F. and Mukouyama, Y. S. (2015). Class 3 semaphorins negatively regulate dermal lymphatic network formation. *Biol. Open* 4, 1194-1205. 10.1242/bio.012302 26319580 PMC4582121

[DEV202747C57] Vaahtomeri, K., Karaman, S., Makinen, T. and Alitalo, K. (2017). Lymphangiogenesis guidance by paracrine and pericellular factors. *Genes Dev.* 31, 1615-1634. 10.1101/gad.303776.117 28947496 PMC5647933

[DEV202747C58] Vallecillo-Garcia, P., Orgeur, M., Vom Hofe-Schneider, S., Stumm, J., Kappert, V., Ibrahim, D. M., Börno, S. T., Hayashi, S., Relaix, F., Hildebrandt, K. et al. (2017). Odd skipped-related 1 identifies a population of embryonic fibro-adipogenic progenitors regulating myogenesis during limb development. *Nat. Commun.* 8, 1218. 10.1038/s41467-017-01120-3 29084951 PMC5662571

[DEV202747C59] Vallecillo-Garcia, P., Orgeur, M., Comai, G., Poehle-Kronnawitter, S., Fischer, C., Gloger, M., Dumas, C. E., Giesecke-Thiel, C., Sauer, S., Tajbakhsh, S. et al. (2023). A local subset of mesenchymal cells expressing the transcription factor Osr1 orchestrates lymph node initiation. *Immunity* 56, 1204-19.e8. 10.1016/j.immuni.2023.04.014 37160119

[DEV202747C60] Walls, J. R., Coultas, L., Rossant, J. and Henkelman, R. M. (2008). Three-dimensional analysis of vascular development in the mouse embryo. *PLoS One* 3, e2853. 10.1371/journal.pone.0002853 18682734 PMC2478714

[DEV202747C61] Walma, D. A. C. and Yamada, K. M. (2020). The extracellular matrix in development. *Development* 147, dev175596. 10.1242/dev.175596 32467294 PMC7272360

[DEV202747C62] Wang, J. F., Zhang, X. F. and Groopman, J. E. (2001). Stimulation of beta 1 integrin induces tyrosine phosphorylation of vascular endothelial growth factor receptor-3 and modulates cell migration. *J. Biol. Chem.* 276, 41950-41957. 10.1074/jbc.M101370200 11553610

[DEV202747C63] Wang, Q., Lan, Y., Cho, E.-S., Maltby, K. M. and Jiang, R. (2005). Odd-skipped related 1 (Odd 1) is an essential regulator of heart and urogenital development. *Dev. Biol.* 288, 582-594. 10.1016/j.ydbio.2005.09.024 16223478 PMC3869089

[DEV202747C64] Wang, G., Muhl, L., Padberg, Y., Dupont, L., Peterson-Maduro, J., Stehling, M., le Noble, F., Colige, A., Betsholtz, C., Schulte-Merker, S. et al. (2020). Specific fibroblast subpopulations and neuronal structures provide local sources of Vegfc-processing components during zebrafish lymphangiogenesis. *Nat. Commun.* 11, 2724. 10.1038/s41467-020-16552-7 32483144 PMC7264274

[DEV202747C65] Wiig, H., Keskin, D. and Kalluri, R. (2010). Interaction between the extracellular matrix and lymphatics: consequences for lymphangiogenesis and lymphatic function. *Matrix Biol.* 29, 645-656. 10.1016/j.matbio.2010.08.001 20727409 PMC3992865

[DEV202747C66] Xiang, M., Grosso, R. A., Takeda, A., Pan, J., Bekkhus, T., Brulois, K., Dermadi, D., Nordling, S., Vanlandewijck, M., Jalkanen, S. et al. (2020). A single-cell transcriptional roadmap of the mouse and human lymph node lymphatic vasculature. *Front. Cardiovasc. Med.* 7, 52. 10.3389/fcvm.2020.00052 32426372 PMC7204639

[DEV202747C67] Yeh, Y. T., Hur, S. S., Chang, J., Wang, K. C., Chiu, J. J., Li, Y. S. and Chien, S. (2012). Matrix stiffness regulates endothelial cell proliferation through septin 9. *PLoS One* 7, e46889. 10.1371/journal.pone.0046889 23118862 PMC3485289

[DEV202747C68] Zhang, L., Zhou, F., Han, W., Shen, B., Luo, J., Shibuya, M. and He, Y. (2010). VEGFR-3 ligand-binding and kinase activity are required for lymphangiogenesis but not for angiogenesis. *Cell Res.* 20, 1319-1331. 10.1038/cr.2010.116 20697430

[DEV202747C69] Zhang, Y., Zhang, Y., Kameishi, S., Barutello, G., Zheng, Y., Tobin, N. P., Nicosia, J., Hennig, K., Chiu, D. K.-C., Balland, M. et al. (2021). The Amot/integrin protein complex transmits mechanical forces required for vascular expansion. *Cell Rep.* 36, 109616. 10.1016/j.celrep.2021.109616 34433061

[DEV202747C70] Zou, Y. R., Kottmann, A. H., Kuroda, M., Taniuchi, I. and Littman, D. R. (1998). Function of the chemokine receptor CXCR4 in haematopoiesis and in cerebellar development. *Nature* 393, 595-599. 10.1038/31269 9634238

