# Supplementary material for: Mesenchymal Osr1+ cells regulate embryonic lymphatic vessel formation
Source: Development. 2024 Sep 2;151(17):dev202747. doi: 10.1242/dev.202747 (PMC11441984; doi:10.1242/dev.202747)
Supplement: Supplementary information [file develop-151-202747-s1.pdf]

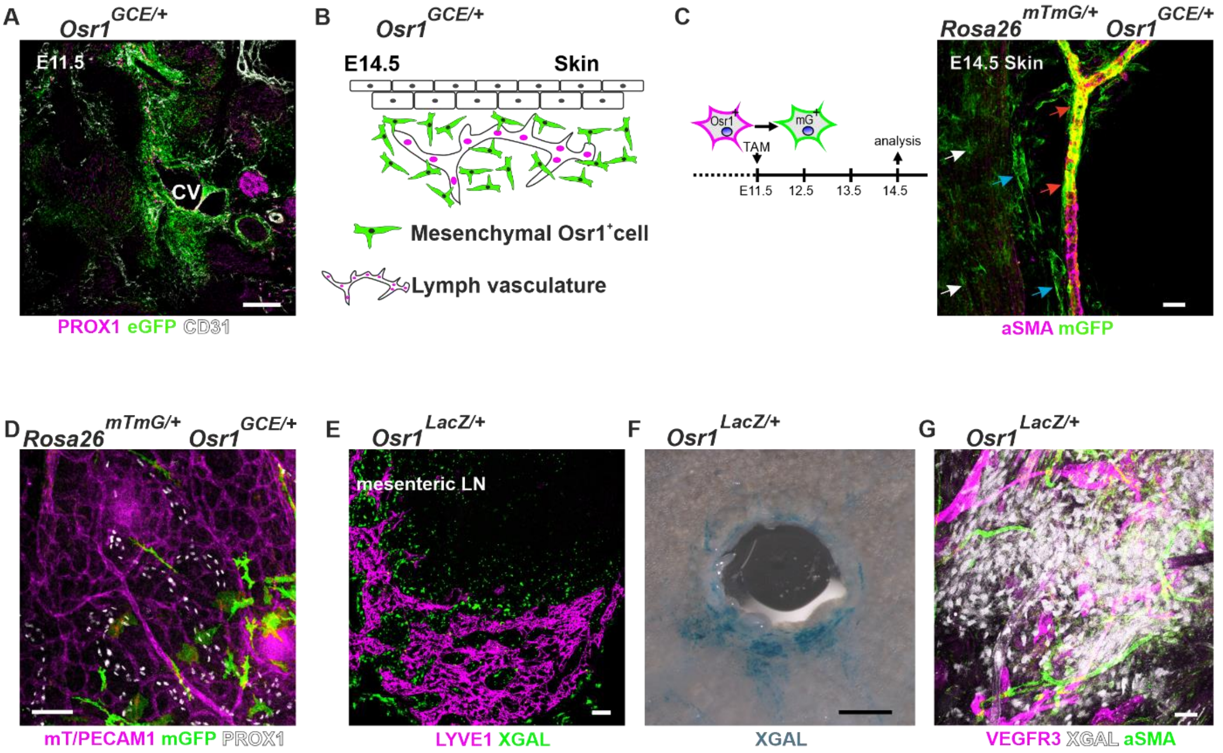


**Fig. S1. related to Fig. 1**. Mesenchymal Osr1+ cells are in close proximity to vascular structures during development, in adults and after trauma. **(A)** Maximal intensity projection of E11.5 100 µm cross-section of Osr1$^{GCE/+}$ embryos. PROX1+ LECs delaminating from the cardinal vein (CV) are surrounded by Osr1+ (eGFP) mesenchymal cells. **(B)** Schematic representation of E14.5 dermis showing lymphatic vasculature surrounded by Osr1+ mesenchymal cells. **(C)** Schematic representation of CreERt2 recombinase induction, at the left. Whole-mount immunofluorescence of E14.5 skin from Rosa26$^{mTmG/+}$ Osr1$^{GCE/+}$ embryos with tamoxifen induction at E11.5, at the right. Osr1 lineage cells are found as dermal fibroblasts (white arrows), as aSMA+ cells of arteria (red arrows) and in close association to veins (blue arrows). **(D)** Representative immunofluorescence image of E14.5 skin from Rosa26$^{mTmG/+}$ Osr1$^{GCE/+}$ embryos showing E11.5 lineage traced Osr1+ cells in association to blood and lymphatic vasculature. **(E)** Osr1+ cells (LacZ+ identified by XGal staining) are found in the medulla of mesenteric lymph nodes in close association with lymphatic vasculature labelled for LYVE1. **(F, G)** Activation of Osr1 expression in mesenchyme upon tissue trauma. Osr1 expression is reactivated in the ear dermis of adult Osr1$^{LacZ/+}$ animals. Immunofluorescence of XGAL stained ear dermis revealed Osr1+ cells intermingling with lymphatic and blood vasculature labelled for VEGFR3 and αSMA in the regenerating area. Representative immunofluorescence images have been captured from at least 3 different embryos. Scale bar represents in **A** 100 µm, in **C** 50 µm, in **D** 100 µm, in **E** 200µm, in **F** 500 µm and in **G** 50 µm.

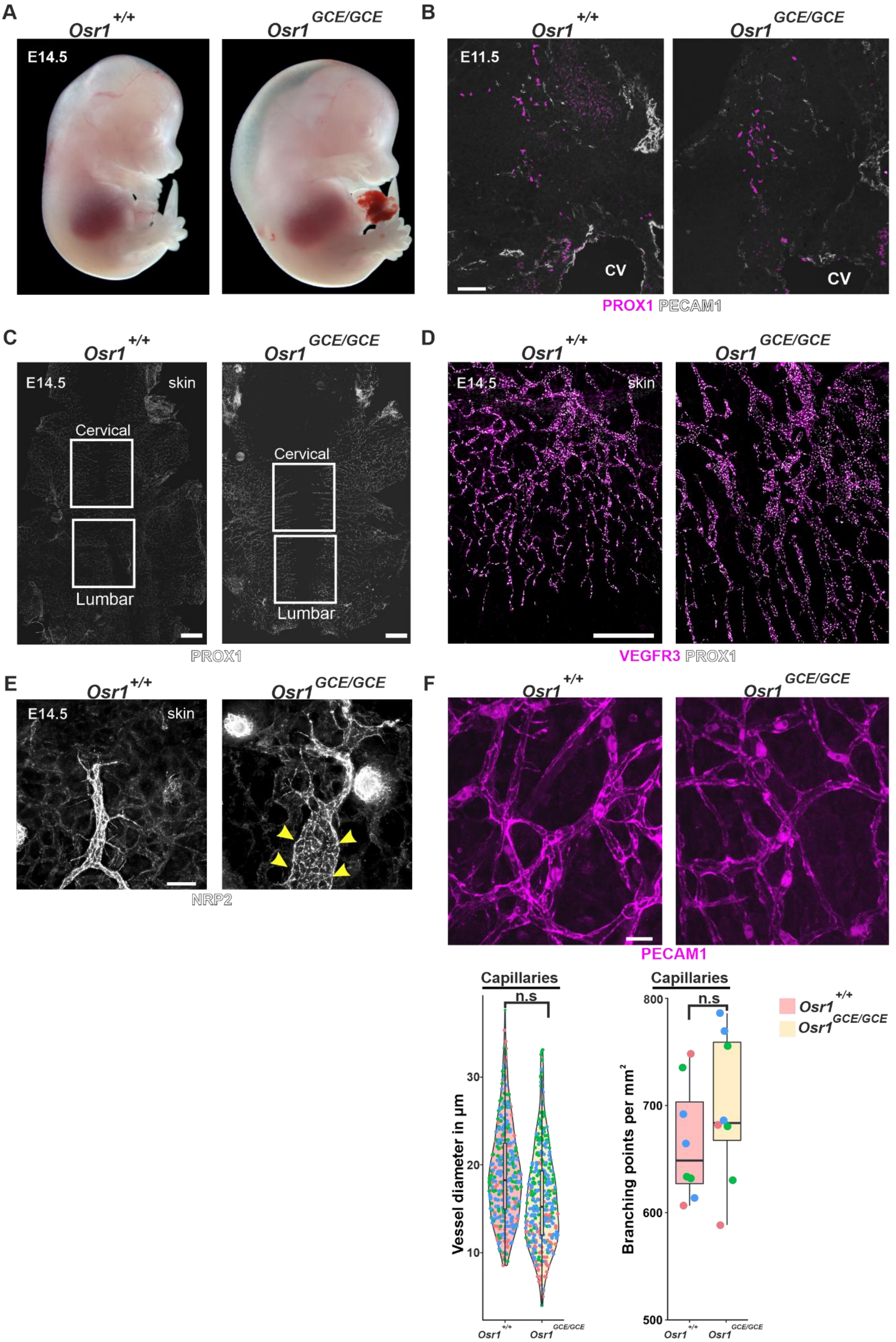


Development • Supplementary information

**Fig. S2. related to Fig. 2.** Lack of Osr1 in mesenchymal cells leads to lymphatic vasculature defects. **(A)** Images of E14.5 Osr1$^{+/+}$ and Osr1$^{GCE/GCE}$ embryos showing prominent back edema. **(B)** Immunofluorescence of E11.5 cross-section shows normal delamination of LECs labelled for PROX1 from the cardinal vein (CV). **(C)** Whole-mount immunofluorescence of E14.5 Osr1$^{+/+}$ and Osr1$^{GCE/GCE}$ skin immunolabelled for PROX1 shows impaired/delayed LECs migration in the cervical and lumbar region. **(D)** Representative micrographs of E14.5 Osr1$^{+/+}$ and Osr1$^{GCE/GCE}$ labelled for VEGFR3 and PROX1 show reduced lymphatic vessel arborization and increase vessel caliber. **(E)** Reduced filopodia at the tips of lymphatic vessels in E14.5 Osr1$^{GCE/GCE}$ embryos after skin whole-mount immunofluorescence using an anti-NRP2 antibody. Arrowheads point to enlarged lymphatic vessels with reduced filopodia. **(F)** Whole-mount immunofluorescence of E14.5 Osr1$^{+/+}$ and Osr1$^{GCE/GCE}$ skin immunolabelled for PECAM1 shows normal blood vessel formation. Quantification of branching points per mm$^2$ and blood vessel caliber in E14.5 Osr1$^{+/+}$and Osr1$^{GCE/GCE}$ skins shown below (n=3). Measurements obtained from the same embryo are represented as dots with the same color. Representative immunofluorescence images have been captured from at least 3 different embryos. Scale bar represents in **B** 100 µm, in **C** 1 mm, in **D** 500 µm, in **E** 50 µm and in **F** 20 µm.

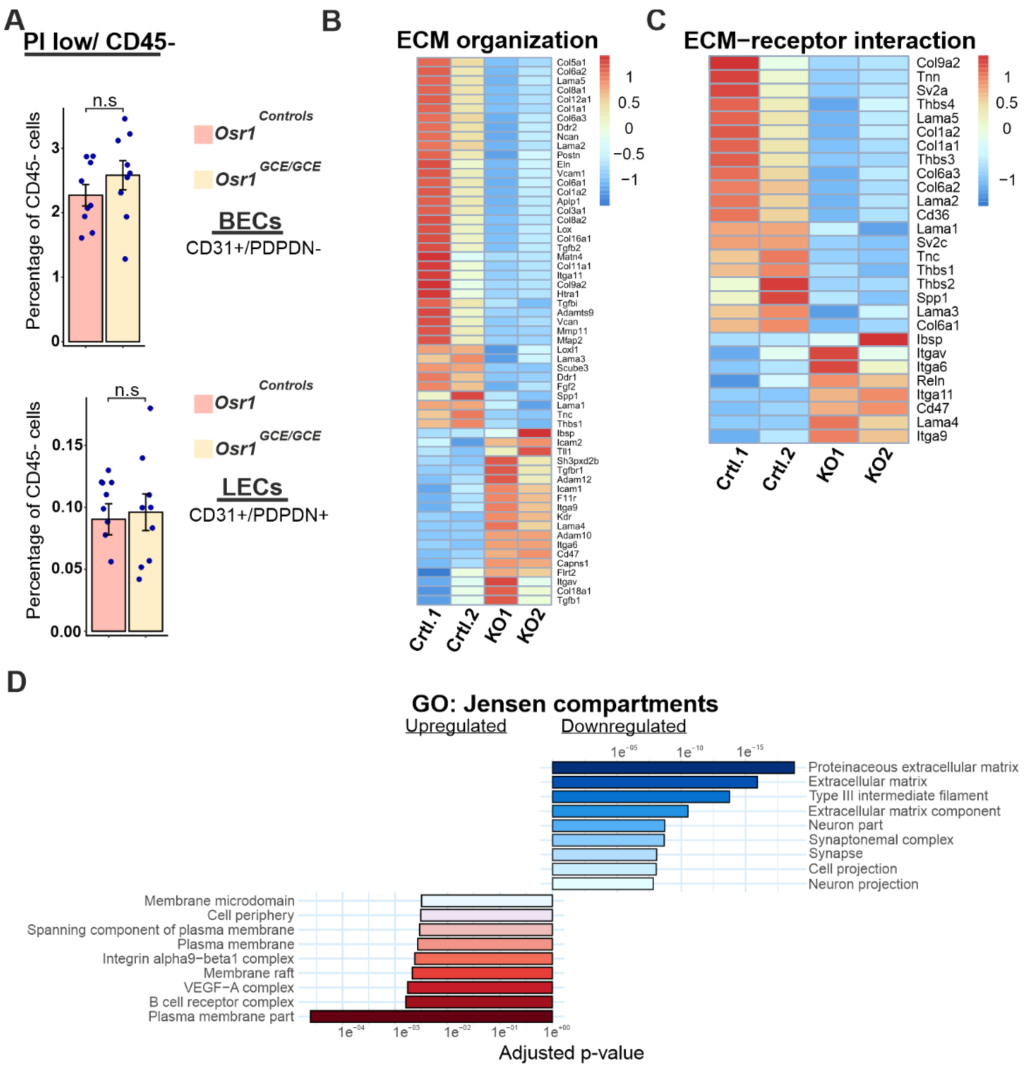


**Fig. S3 related to Fig. 3.** Transcriptional adaptations of LECs to an environment lacking Osr1. **(A)** Quantification of E13.5 LEC (PDPN+ CD31+) and BEC (PDPN- CD31+) relative percentage from Osr1$^{controls}$ (Osr1$^{+/+}$ and Osr1$^{GCE/+}$) and Osr1$^{GCE/GCE}$ embryos (n=9). **(B, C)** Heatmap depiction of TPM values for the genes associated with the GO terms ECM organization and ECM-receptor interaction detected after E13.5 LECs RNA-seq. Raw scaled normalization is represented at the right. **(D)** Bar plot representation of GO analysis for Jensen compartments performed in genes up- or downregulated in LECs from Osr1$^{GCE/GCE}$ embryos. Terms were ranked by their adjusted p-value. In **A**, p-values were obtained from student-t tests. Error bar represents s.e.m., * p<0.05, and ** p<0.01.

Development • Supplementary information

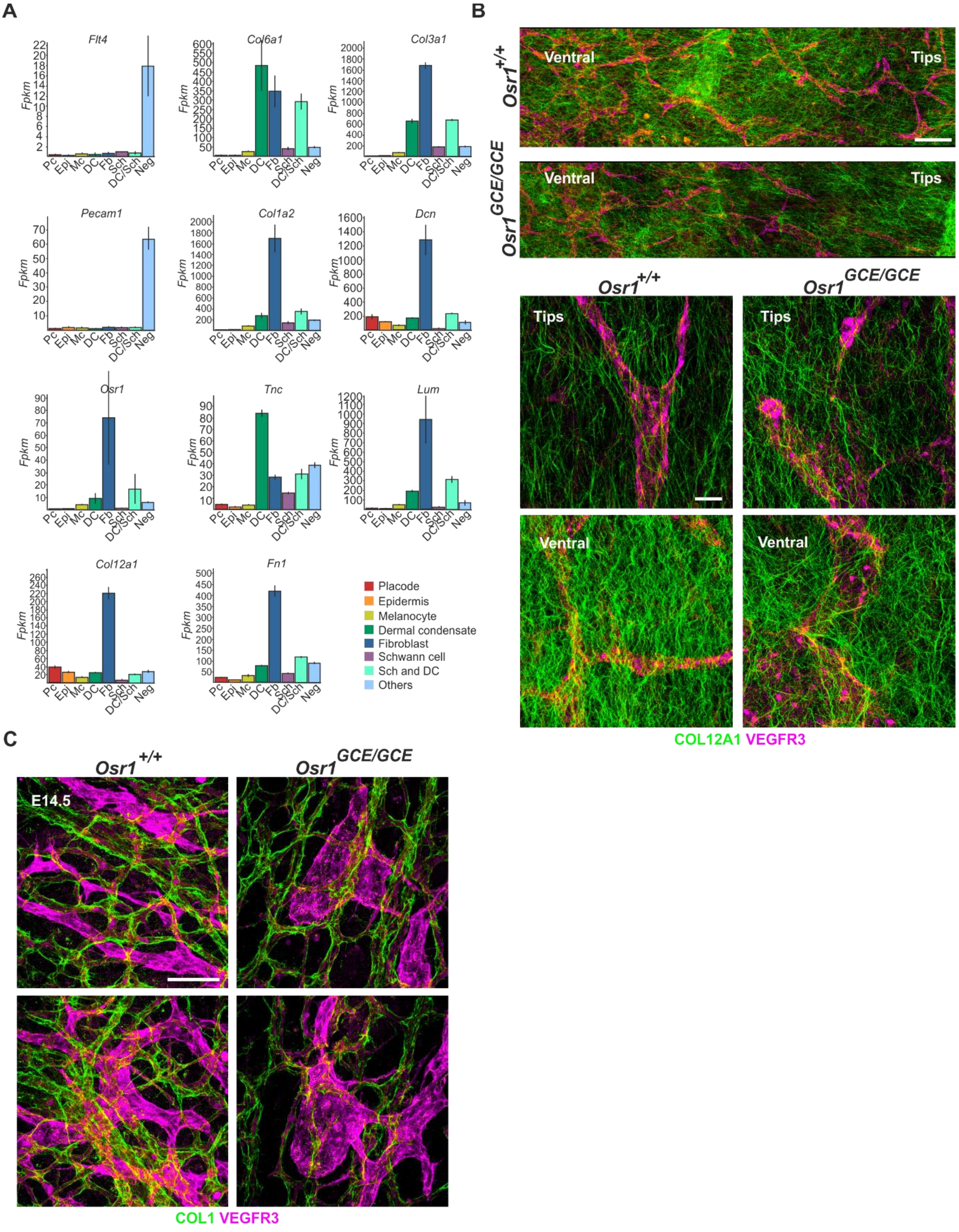


**Fig. S4 related to Fig. 5.** Impaired dermal ECM in E14.5 Osr1$^{GCE/GCE}$ embryos. **(A)** Bar plot depiction of relative expression for selected genes in E14.5 skin cell populations (legend shown bottom right). Expression is represented in FPKM values obtained from RNA-seq analyses (37). **(B, C)** Whole-mount immunofluorescence of E14.5 Osr1$^{+/+}$ and Osr1$^{GCE/GCE}$ skin immunolabelled for the ECM protein COL12A1, the lymphatic vessel marker VEGFR3 and COL1 that labels blood vessel basal lamina. Representative immunofluorescence images have been captured from at least 3 different embryos. Scale bar represents in **B,** 100 µm, 20 µm (below) and in **C,** 50 µm.

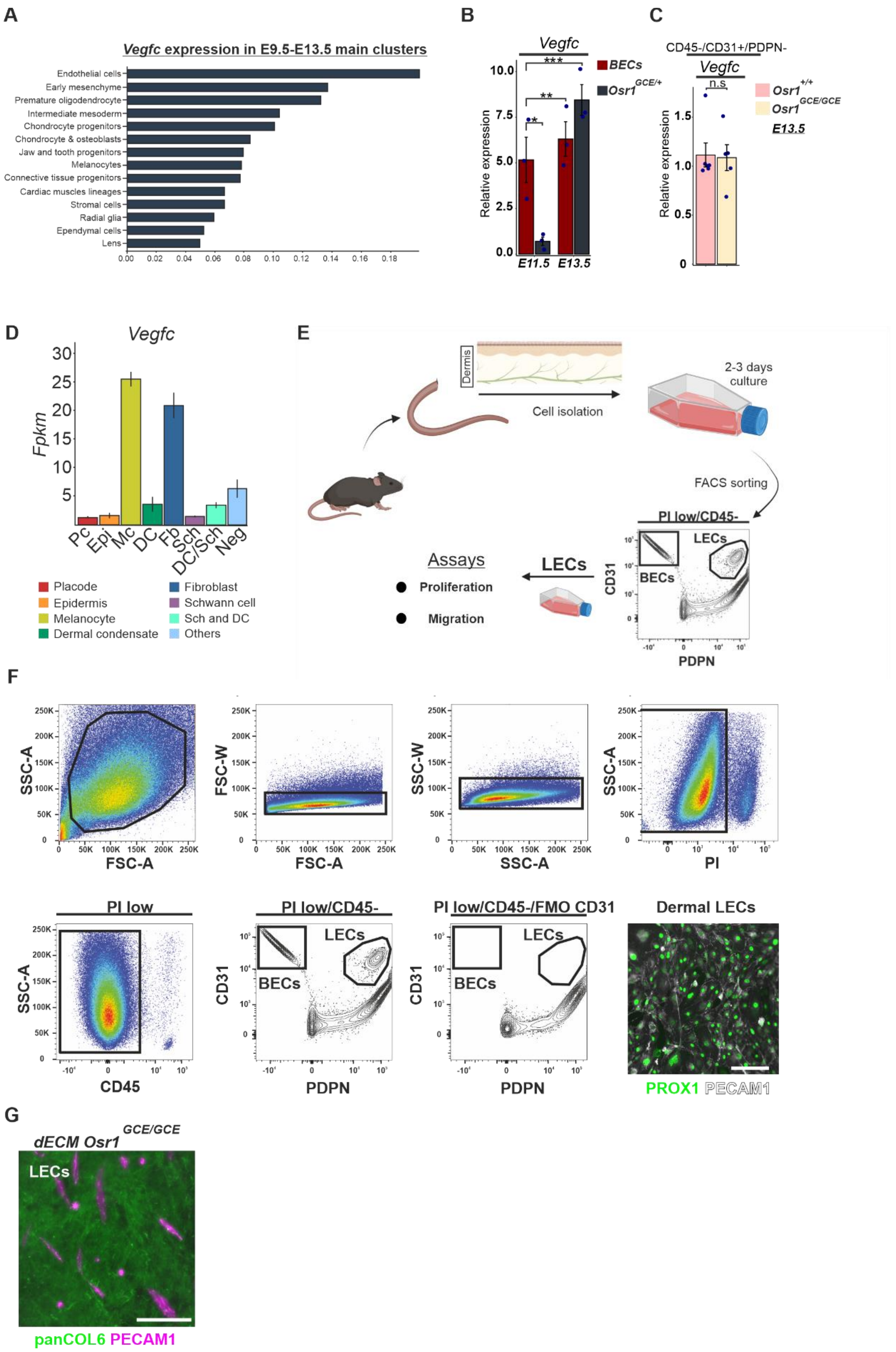


**Fig. S5 related to Fig. 6.** Mesenchymal Osr1+ cells promote LEC proliferation at the migrating front via the ECM. **(A)** Bar plot depiction of relative Vegfc expression in embryonic subpopulations from E9-E13.5 tissues obtained from scRNA-seq analyses (42). **(B)** Relative expression of Vegfc transcripts measured by RT-qPCR in FACS isolated BECs (CD31+PDPN-) and Osr1+ cells from E11.5 and E13.5 Osr1$^{GCE/+}$

Development • Supplementary information

embryonic tissues. N=3. **(C)** Relative expression of Vegfc transcripts measured by RT-qPCR in FACS isolated BECs (CD31+PDPN-) from E13.5 Osr1 controls (Osr1$^{+/+}$ and Osr1$^{GCE/+}$) or Osr1$^{GCE/GCE}$ embryos. N=5. **(D)** Bar plot depiction of Vegfc relative expression in E14.5 skin cell populations. Expression is represented in FPKM values obtained from RNA-seq analysis (37). **(E)** Schematic representation of tail skin dermal LEC (tdLEC) isolation from C57Bl/6 adult animals and culturing for subsequent migration and proliferation assays. **(F)** FACS gating strategy for tdLECs isolation using CD45, CD31 and PDPN antibodies. Immunofluorescence of culture tdLECs using PECAM1 and PROX1 antibodies confirms LEC purity after FACS isolation. **(G)** Representative immunofluorescence of decellularized ECM (dECM) produced by E13.5 Osr1$^{GCE/+}$ cells after 2 days of tdLEC culturing on dECM. An anti-panCOL6 antibody was used for ECM labeling and an anti-PECAM1 antibody for LEC labeling. In **B**, p values were obtained from student-t tests. Error bar represents s.e.m. and * $p< 0.05$, ** $p< 0.01$ and *** $p< 0.001$. Scale bar in **F** and **G** represents 200 µm.

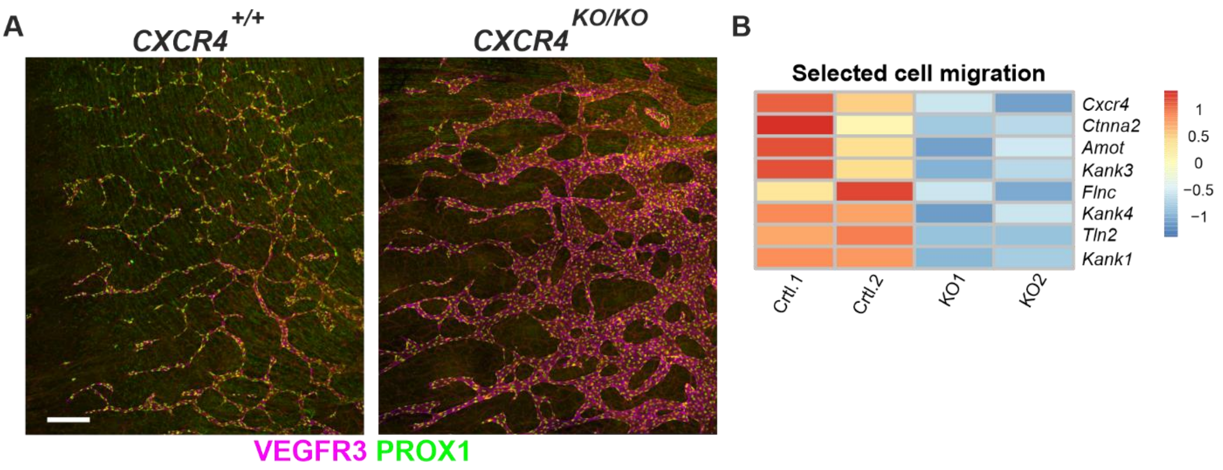


**Fig. S6. related to Fig. 7.** CXCR4 controls LEC dermal migration. **(A)** Whole-mount immunofluorescence of E14.5 CXCR4$^{+/+}$ and CXCR4$^{KO/KO}$ skin labelled for the lymphatic vessel markers VEGFR3 and PROX1. **(B)** Heatmap depiction of TPM values for selected genes involved in cell migration being deregulated in E13.5 LECs RNA-seq analysis. Raw scaled normalization is represented at the right. Representative immunofluorescence images have been captured from at least 3 different embryos. Scale bar in **A** represents 200 µm.

**Table S1.**

| Gene name | Mean TPM | TPM Rank |
|---|---|---|
| *Col4a1* | 1068 | 73 |
| *Cdh5* | 1044 | 75 |
| *Fos* | 976 | 89 |
| *Col18a1* | 924 | 96 |
| *Fosb* | 837 | 112 |
| *Plxnd1* | 808 | 118 |
| *Junb* | 739 | 133 |
| *Col4a2* | 727 | 136 |
| *Mmrn1* | 706 | 141 |
| *Nrp2* | 619 | 162 |
| *Flt4* | 583 | 184 |
| *Egr1* | 499 | 231 |
| *Tie1* | 476 | 251 |
| *Prox1* | 312 | 404 |
| *Lyve1* | 293 | 448 |
| *Kdr* | 490 | 240 |
| *Pecam1* | 387 | 312 |
| *Nrp1* | 21 | 6073 |
| *Cd44* | 15 | 8408 |
| *Postn* | 131 | 1238 |
| *Lum* | 72 | 2652 |
| *Pdgfrα* | 44 | 4333 |
| *Dcn* | 37 | 5009 |
| *Osr1* | 6 | 11724 |

## Table S2. Primary antibodies.

| Antibody | Clone | Conjugate | Concentration/ Dilution | Source |
|---|---|---|---|---|
| **Mouse anti-MyHC** | Monoclonal | Unconjugated | 1:500 | Chemicon |
| **Chicken anti-GFP** | Polyclonal | Unconjugated | 1:1000 | Aves |
| **Rabbit anti-α-SMA** | Polyclonal | Unconjugated | 2 µg $ml^{-1}$ | Abcam |
| **Hamster anti-Pecam1** | Polyclonal | Unconjugated | 1:250 | DSHB (2H8) |
| **Rat anti-Pecam1** | Mec 13.3 | Unconjugated | 1:100 | Biolegend |
| **Goat anti-collagen I** | Polyclonal | Unconjugated | 1:400 | Novus Biologicals |
| **Hamster anti PDPN** | Polyclonal | Unconjugated | 1:100 | DSHB (8.1.1) |
| **Rat anti CD45** | 30-F11 | Unconjugated | 1:100 | Biolegend |
| **Rabbit anti TNC** | Polyclonal | Unconjugated | 1:200 | Chemicon |
| **Rabbit anti PROX1** | Polyclonal | Unconjugated | 1:200 | ReliaTech |
| **Rabbit anti LYVE1** | Polyclonal | Unconjugated | 1:200 | Abcam (33682) |
| **Mouse anti VCAM1** | Polyclonal | Unconjugated | 1:100 | DSHB (P3C4) |
| **Goat anti VEGFR3** | Polyclonal | Unconjugated | 1:200 | R&D System |
| **Goat anti CCL21** | Polyclonal | Unconjugated | 10 µg $ml^{-1}$ | R&D System (AF457) |
| **Rat anti PDGFRβ** | APB5 | PE | 1:100 | Biolegend |
| **Rabbit anti-Col12a1** | Polyclonal | Unconjugated | 1:500 | M. Koch |
| **Rabbit anti Ki-67** | Polyclonal | Unconjugated | 1:200 | Abcam |
| **Goat anti Nrp2** | Polyclonal | Unconjugated | 10 µg $ml^{-1}$ | R&D System (AF2215) |
| **Goat anti hPROX1** | Polyclonal | Unconjugated | 1:100 | R&D System(AF2727) |
| **Rabbit anti panCol6** | Polyclonal | Unconjugated | 1:250 | Abcam |

## Table S3. FACS antibodies

| Antibody | Clone | Conjugate | Concentration/ Dilution | Source |
|---|---|---|---|---|
| **Rat anti-T119** | TER-119 | APC | 1:100 | eBioscience |
| **Rat anti CD31** | 390 | APC | 1:100 | eBioscience |
| **Rat anti PDPN** | PMab2 | Alexa Fluor 594 | 1:100 | Biolegend |
| **Rat anti-CD45** | 30-F11 | APC | 1:100 | eBioscience |

Development • Supplementary information

## Table S4. Secondary antibodies.

| Antibody | Conjugate(s) | Source |
|---|---|---|
| **Donkey anti-mouse** | Alexa Fluor 488, 568 and 680 | Molecular Probes |
| **Donkey anti-rabbit** | Alexa Fluor 488, 568 and 647 | Molecular Probes |
| **Donkey anti-goat** | Alexa Fluor 488, 568 and 680 | Molecular Probes |
| **Goat anti-hamster** | Alexa Fluor 488, 568 | Molecular Probes |
| **Donkey anti-rat** | Alexa Fluor 488, 568, 647 | Molecular Probes |
| **Donkey anti-chicken** | Alexa Fluor 488 | Molecular Probes |
| **Donkey anti-sheep** | Alexa Fluor 488, 568 | Molecular Probes |

## Table S5. Primer sequences (RT-qPCR).

| Gene | Forward | Reverse |
|---|---|---|
| ***Gadph*** | CTGCACCACCAACTGCTTAG | GGATGCAGGGATGATGTTCT |
| ***Cxcl12*** | GCTCCACCCACAAGGTTAAG | CTGGCAGAAGGCCTTGAATA |
| ***Osr1*** | GCACACTGATGAGCGACCT | TGTAGCGTCTTGTGGACAGC |
| ***Ccl21*** | CCAAGTTTAGGCTGTCCCATC | TTAGAGGTTCCCCGGTTCTT |
| ***Flt4*** | TCATTGGGGGCCTCTCCATA | GTCCCCCAGGATCTCCACTA |
| ***Prox1*** | TCGCAGCTCATCAAGTGGTT | TAGTGCATGTTGAGGGCTCG |
| ***Vegfc*** | CACCAGCACAGGTTACCTCA | TGCTGGCAGAGAACGTCTAA |

## Table S6. Dermal fibroblast intersection analysis.

List of genes found upregulated in E13.5 *Osr1*$^{GCE/+}$ cluster 7 (log2FC ≥ 0.2 and percFC ≥ 0 and deregulated in E13.5 *Osr1*$^{GCE/GCE}$ mesenchymal cells (log2FC ≥ 0.2 and padj ≤ 0.1).

Available for download at
https://journals.biologists.com/dev/article-lookup/doi/10.1242/dev.202747#supplementary-data

## Table S7. Mesenchymal-LEC ligand-receptor pair analysis.

List of ligand-receptor pairs found after analysis of deregulated genes in E13.5 *Osr1*$^{GCE/GCE}$ mesenchymal cells (511 genes) and in E13.5 LECs (1386 genes) from *Osr1*$^{controls}$ and *Osr1*$^{GCE/GCE}$ embryos. For each ligand-receptor pair, the role as a receptor or ligand and the expression (upregulated or downregulated) in E13.5 LECs and mesenchymal cells are defined.

Available for download at
https://journals.biologists.com/dev/article-lookup/doi/10.1242/dev.202747#supplementary-data

Development • Supplementary information
