# Supplementary material for: Mesenchymal Osr1+ cells regulate embryonic lymphatic vessel formation
Source: Development. 2024 Sep 2;151(17):dev202747. doi: 10.1242/dev.202747 (PMC11441984; doi:10.1242/dev.202747)
Supplement: Supplementary file 1 [file develop-151-202747_review_history.pdf]

Mesenchymal Osr1^{+} cells regulate embryonic lymphatic vessel formation

Pedro Vallecillo-Garcia, Mira Nicola Kühnlein, Mickael Orgeur, Nils Rouven Hansmeier, Georgios Kotsaris, Zarah Gertrud Meisen, Bernd Timmermann, Claudia Giesecke-Thiel, René Hägerling and Sigmar Stricker
DOI: 10.1242/dev.202747

Editor: Benoit Bruneau

Review timeline

Original submission:	31 January 2024
Editorial decision:	20 February 2024
First revision received:	10 June 2024
Editorial decision:	26 June 2024
Second revision received:	17 July 2024
Accepted:	17 July 2024

Original submission

First decision letter

MS ID#: DEVELOP/2024/202747

MS TITLE: Mesenchymal Osr1+ cells regulate embryonic lymphatic vessel formation

AUTHORS: Pedro Vallecillo Garcia, Mira Nicola Kuehnlein, Mickael Orgeur, Nils Rouven Hansmeier, Georgios Kotsaris, Bernd Timmermann, Claudia Giesecke-Thiel, Rene Haegerling, and Sigmar Stricker

I have now received all the referees' reports on the above manuscript, and have reached a decision. The referees' comments are appended below, or you can access them online: please go to BenchPress and click on the 'Manuscripts with Decisions' queue in the Author Area.

As you will see, the referees express considerable interest in your work, but have some significant criticisms and recommend a substantial revision of your manuscript before we can consider publication. If you are able to revise the manuscript along the lines suggested, which may involve further experiments, I will be happy receive a revised version of the manuscript. Your revised paper will be re-reviewed by one or more of the original referees, and acceptance of your manuscript will depend on your addressing satisfactorily the reviewers' major concerns. Please also note that Development will normally permit only one round of major revision. If it would be helpful, you are welcome to contact us to discuss your revision in greater detail. Please send us a point-by-point response indicating your plans for addressing the referees' comments, and we will look over this and provide further guidance.

Please attend to all of the reviewers' comments and ensure that you clearly highlight all changes made in the revised manuscript. Please avoid using 'Tracked changes' in Word files as these are lost in PDF conversion. I should be grateful if you would also provide a point-by-point response detailing how you have dealt with the points raised by the reviewers in the 'Response to Reviewers' box. If you do not agree with any of their criticisms or suggestions please explain clearly why this is so.

© 2024. Published by The Company of Biologists under the terms of the Creative Commons Attribution License (https://creativecommons.org/licenses/by/4.0/).

Reviewer 1

Advance summary and potential significance to field

This study from Vallecillo-Garcia and colleagues describes a novel role for Osr1-positive mesenchymal cells during lymphatic vascular development. Osr1-positive mesenchymal cells are demonstrated to be in close association with lymphatic endothelial cells throughout development of the mouse embryo and are proposed to regulate lymphatic endothelial cell proliferation and migration through the production of extracellular matrix components and growth/migration cues including VEGFC and CXCL12. Several important points remain to be addressed/validated to fully support the authors' claims, particularly with regard to ascribing roles to VEGFC and CXCL12 produced by Osr1-positive mesenchymal cells. These are detailed below.

Comments for the author

Major comments:

1. Figure 1: Are Osr1-positive mesenchymal cells any more closely associated with the developing lymphatics than any other structures in the embryo? Are they more closely associated with lymphatic vessels than with blood vessels? A quantitative analysis of their localisation with respect to different structures would be informative.

2. Fig 1D: PECAM staining in this panel is not convincing, one would expect to see both blood vessels and lymphatic vessels using this marker.

3. Figures 2A and 2B: The lymphatic vascular defects, or lack thereof, in these panels should be quantified with respect to migration and/or numbers of PROX1-positive cells outside the CVs, particularly where significance is claimed.

4. Page 6, Supp Fig 2E: The number of filopodia should be counted to make the claim that they are reduced. It appears that filopodia are in fact, still formed (Supp Fig 2E).

5. Page 8, Fig 3G: The GO analysis reveals that Extracellular Matrix Organisation is enriched in both up and down-regulated genes. Are distinct ECM terms enriched in the up- versus down-regulated genes of Osr1 GCE/GCE embryos?

6. Figure 3D-G: Given the enrichment of GO terms associated with axon guidance and nervous system development, is innervation of the skin also affected in Osr1GCE/GCE embryos? One might expect this to be the case.

7. Could the authors postulate as to why genes important for lymphatic vascular development are upregulated in mutant embryos when lymphangiogenesis is affected?

8. Figure 5: Inclusion of higher magnification images of the ECM components assessed in the skin of WT and GCE/GCE mice would be informative for the reader to gain deeper insight to the nature of the ECM defects. The orientation of the images shown with respect to the embryo should also be indicated.

9. Page 11: Fig Supp 4C, Figure 5D: Do the authors intend to differentiate between reduced accumulation of COL1 protein and impaired COL1 deposition? It's not clear whether the images shown represent this, or whether they simply illustrate reduced levels of COL1 in the vicinity of blood vessels.

10. The claim that LECs positioned at the front of the migrating dermal vessels proliferate more than those positioned more laterally should be supported by quantification. Details regarding exactly where cell counts were performed need to be provided in the figure legend and the Methods. Figure 6E: n=4? If each colour represents a field from one embryo, the n should be 3 as currently shown. Additional details of the method used to quantify proliferation and how many cells were counted should be added to the Methods section. At present the data is not completely convincing. Likewise, the Methods would benefit from more detail describing the proliferation

© 2024. Published by The Company of Biologists under the terms of the Creative Commons Attribution License (https://creativecommons.org/licenses/by/4.0/).

assays illustrated in Fig 6G/H. In which media were the cells cultured in Figure 6G? How many cells were counted? Panel G: what is the PROX1-negative, Ki67-positive staining in these panels if these represent LECs grown on de-cellularised matrix? Are total levels of VEGFC altered in GCE/GCE skin? Could migration assays be performed on the GCE/GCE compared to GCE/+ de-cellularised matrix?

11. Figure 7B: The migration assays shown suggest a delay to migration promoted by GCE/GCE conditioned media. Is this predicted to be sufficient to account for reduced LEC migration observed in embryos? Would it be more relevant to look at the migration of LECs on Osr1-deficient mesenchymal cells?

12. While the data shown in Figure 7E demonstrate a similar phenotype in CXCR4-deficient embryos to that observed in Osr1 mutant embryos, they do not definitively ascribe the phenotype observed in Osr1GCE/GCE mutants to a loss of CXCL12 in Osr1 deficient mesenchymal cells. This should be investigated using conditional mutant mouse lines, or the claim that CXCL12/CXCR4 signaling is required for LEC dorsal migration downstream of mesenchymal Osr1 should be toned down. The well established roles of CXCL12/CXCR4 signaling in blood vascular development mean that effects observed in the blood vasculature of CXCR4 KO mice may also impact the lymphatic phenotype. The blood vascular phenotype of CXCR4 KO mice should also be shown.

Reviewer 2

Advance summary and potential significance to field

The coordination of lymphatic endothelial cell (LEC) migration in the 3D space of an embryo remains incompletely understood. Vallecillo-Garcia et. al. has characterised the role of Osr1+ mesenchymal cells in regulating LECs behaviours.
They determined that Osr1+ cells appear on the LECs migratory path prior to the initiation of LEC migration. Furthermore, mice deficient in Osr1+ show defects in lymphatic vessel sprouting and morphology. Transcriptional analysis of LECs and Osr1+ mesenchymal cells identified ligand and receptor pairs that could explain the cellular interaction. As Osr1+ cells express Vegfc, the authors investigated VEGFC/VEGFR3 signalling in LECs from Osr1 KO embryos where they observed an increase in the expression of the target genes, suggesting that the interaction of Osr1+ cells with LECs is not a positive regulator of this signalling cascade. In addition, the authors showed that CXCR4 has similar phenotypes in migration but not the proliferation of LECs as the Osr1 KO embryo. They have shown that mesenchymal cells support LEC by maintaing the ECM scaffold. Together, the study concluded that Osr+ cells non-autonomously coordinate proliferation via ECM deposition and migration of LECs via CXCR4-CXCL12 signalling.
This is an interesting study which provides significant insights into how LEC migration is coordinated by uncovering the interaction of Osr1+ mesenchymal cells with LECs; however, some points require further clarification before publication.

Comments for the author

The main observation of the defects in lymphatic vessels is done at 14.5, when the embryos show profound oedema. Swelling of the tissues and changes in the interstitial pressure by itself can induce lymphatic phenotypes (Urner et al., 2018 EMBO J). It would be important to confirm which of the observed phenotypes are due to Osr1 KO and which due to oedema. A suggestion would be to assess the proliferation and migration phenotypes prior to oedema formation. In addition quantification of pTD LECs would be informative.

The authors noted puzzling results wherein Vegfc is downregulated in Osr1 KO mesenchymal cells, yet the targets of VEGFC/VEGFR3 are upregulated at the transcriptional level in LECs. It would be beneficial to include a discussion of what these results indicate with their potential biological implications. In addition, to better understand these phenotypes, it would be beneficial to supplement these observations with an analysis of the levels of pERK or pAKT in LECs.

The authors conducted elegant experiments using decellularised ECM or cell-conditioned media to assess how Osr1+ mesenchymal cells regulate LEC proliferation vs. migration. To further understand whether VEGFC is indeed secreted from Osr1+ cells and if this is inhibited in KO cells performing

© 2024. Published by The Company of Biologists under the terms of the Creative Commons Attribution License (https://creativecommons.org/licenses/by/4.0/).

western blot analysis for VEGFC on the culture medium. Such an experiment would also elucidate whether Osr1 cells exclusively secrete VEGFC and if the ECM they produce is necessary for VEGFC activation/processing.

The authors state that there is a close association between the Osr1+ mesenchymal cells and LEC. Do these cells make direct contact or LECs only need to sense the ECM? Providing a more detailed and higher-resolution analysis of the interaction could be beneficial.

It has been shown that LEC clusters form in the mid-line and ahead of LEC migrating front in the back skin (Martinez-Corral et al, 2015 Circ Res., Pichol-Thievend et al, 2018 Development). Do the Osr1+cell associate with these LEC clusters? Could they serve as an indication of where these clusters will form?

I would like to compliment the authors on the images in the paper, particularly the figure S2E, as well as the whole back skin overview in Figure S2C helps to appreciate the overall phenotype.

Minor
Figure 3H it would be informative to expand this map with additional markers for LEC vs BEC. This would further validate the correct cell type enrichment in LECs.

Please clarify this sentence: “Contradictory to the notion that reduced Vegfc in mesenchymal cells may be responsible for the lymphatic vessel defects observed in Osr1 deficient embryos, we observed an increase in Flt4 expression in LECs of E13.5 Osr1GCE/GCE embryos (figure 3F)” for better comprehension.

One could consider using “Zebrafish” instead of “zebra fish” to align with the common terminology.

First revision

Author response to reviewers' comments

Author Responses to reviewers’ questions

We would like to thank the reviewers for critically assessing our manuscript and providing us with insightful suggestions to improve our paper. We hope that below we can answer and clarify all issues raised. Please find our point-by-point responses to your questions and suggestions below.

Reviewer 1 Advance Summary and Potential Significance to Field...

This study from Vallecillo-Garcia and colleagues describes a novel role for Osr1-positive mesenchymal cells during lymphatic vascular development. Osr1-positive mesenchymal cells are demonstrated to be in close association with lymphatic endothelial cells throughout development of the mouse embryo and are proposed to regulate lymphatic endothelial cell proliferation and migration through the production of extracellular matrix components and growth/migration cues including VEGFC and CXCL12. Several important points remain to be addressed/validated to fully support the authors’ claims, particularly with regard to ascribing roles to VEGFC and CXCL12 produced by Osr1-positive mesenchymal cells. These are detailed below.

Reviewer 1 Comments for the Author...

Major comments:
1. Figure 1: Are Osr1-positive mesenchymal cells any more closely associated with the developing lymphatics than any other structures in the embryo? Are they more closely associated with lymphatic vessels than with blood vessels? A quantitative analysis of their localisation with respect to different structures would be informative.
We thank the reviewer for highlighting this point. We would like to point out that *Osr1* expression

© 2024. Published by The Company of Biologists under the terms of the Creative Commons Attribution License (https://creativecommons.org/licenses/by/4.0/).

is not exclusively associated with the developing lymph vasculature; we also highlighted this in the introduction stating that *Osr1* is expressed in mesenchymal cells at many positions in the embryo, where it is associated with organogenesis in different contexts, such as myogenesis in the limb or lymph node initiation (as we have described earlier). In this manuscript, we specifically focus on the association of *Osr1*-expressing cells with the developing lymphatic vasculature. Nevertheless, we agree with the reviewer that the comparative localization of Osr1+ cells with respect to the blood vasculature is of interest here.
However, at E11.5 *Osr1* expression is found along the rostro-caudal axis in the dorsal region of the cardinal vein, where the first LECs from venous origin are delaminating and migrating. Similarly, at E12.5 the primordial thoracic duct (pTD) and further dorsally migrating LECs are surrounded by Osr1+ cells, whereas blood capillaries formed outside of this region are not surrounded by Osr1+ mesenchymal cells.
At the stage E14.5, however *Osr1* is highly expressed in dermal fibroblasts, which are in close proximity to lymphatic vessels and vein capillaries in the skin.
We want to emphasize that Osr1+ cells are not more proximal to LECs than BECs in the region where Osr1+ cells and endothelial cells are found (Figures 1A and S1A, at E11.5 Osr1 cells equally close to BECs and LECs) but that due to the regionalized distribution of Osr1+ cells and venous-derived LECs, LECs are embedded in Osr1+ mesenchymal cells from E11.5 to E14.5. Contrary, blood vessels already formed at this stage in other regions of the embryo (e.g. distal limb bud, dorsal root ganglion or spinal cord) are devoid of Osr1+ mesenchymal cells.
Therefore, we think a quantification of relative distance would not be informative here. Nevertheless, why blood vessels react less to *Osr1*-deficiency compared to lymphatic vasculature is an interesting question, but not a focus of this study.

2. Fig 1D: PECAM staining in this panel is not convincing, one would expect to see both blood vessels and lymphatic vessels using this marker.

We would like to clarify this point. The reviewer is right, the purple staining in Figures 1D and S1D is not specific for endothelial cells (BECs or LECs). For these experiments, we have used the line *Rosa26*$^{mT/mG}$ *Osr1*$^{Cre/+}$. After Cre activation only Osr1+ cells are recombined and expressed a membrane GFP. The rest of the cells in the embryo still express a membrane Tomato that can be detected in the red channel. Due to the restriction of fluorescence channels available in our microscope, we have stained PECAM1+ cells in the same channel as Tomato+ cells (Figure 1D and S1D, mT/PECAM1). In order to specifically stain LECs in the skin, what was our main focus, we have counterstained with PROX1 (Figure 1E). Thus, we observed in purple PECAM1^{high} vasculature in the skin and also many other structures that are not PECAM1 positive but Tomato positive.

3. Figures 2A and 2B: The lymphatic vascular defects, or lack thereof, in these panels should be quantified with respect to migration and/or numbers of PROX1-positive cells outside the CVs, particularly where significance is claimed.

We thank the reviewer for highlighting this important point. We have now analyzed the amount of PROX1+ cells inside and outside of the cardinal vein in E11.5 *Osr1*$^{+/+}$ and *Osr1*$^{GCE/GCE}$ KO embryos. We have now quantified LECs at E11.5. At E11.5, we do not observe a significant reduction of LECs outside of the cardinal vein (Figure 2A). We also now analyzed the amount of VEGFR3+ lymphatic vasculature formed distally from the pTD close to the back skin as a readout for LEC migration at the stage E12.5, i.e. before back edema is visible in *Osr1*$^{GCE/GCE}$ embryos. For that, we have assessed the amount of VEGFR3+ lymphatic vasculature in 100 µm thick sagittal sections after immunofluorescence. As shown now in Figure 2B, we observed reduced VEGFR3+ lymphatic vasculature in the most dorsal part of E12.5 *Osr1*$^{GCE/GCE}$ embryos indicating that lymphatic vasculature defects in *Osr1*-deficient embryos appear before a back edema can be observed at E13.5.

4. Page 6, Supp Fig 2E: The number of filopodia should be counted to make the claim that they are reduced. It appears that filopodia are in fact, still formed (Supp Fig 2E).

We have now quantified the number of filopodia per PROX1+ cell in the distal part of the migrating zone in E14.5 *Osr1*$^{GCE/+}$ and *Osr1*$^{GCE/GCE}$ embryos. These results are now displayed in Figure 2I

© 2024. Published by The Company of Biologists under the terms of the Creative Commons Attribution License (https://creativecommons.org/licenses/by/4.0/).

and show that, while filopodia indeed still form in E14.5 *Osr1*$^{GCE/GCE}$ embryos, filopodia formation in LECs at the migrating front in the skin are significantly reduced.

5. Page 8, Fig 3G: The GO analysis reveals that Extracellular Matrix Organisation is enriched in both up and down-regulated genes. Are distinct ECM terms enriched in the up- versus down-regulated genes of Osr1 GCE/GCE embryos?

This is an interesting question; in figure 3G, we performed gene ontology analyses for the term Biological Process using separately downregulated (blue bars) and upregulated (red bars) genes found in LECs from E13.5 *Osr1*$^{GCE/GCE}$ embryos. Therefore, both set of genes are distinct and contain different genes.

Below, we have listed the downregulated and upregulated "Extracellular Matrix Organization" genes found in LECs from E13.5 *Osr1*$^{GCE/GCE}$ embryos. In line with a transcriptional upregulation of genes involved in vascular development (figure 3G), ECM or ECM-interaction genes found upregulated are in part those generally found highly expressed by lymphatic endothelial cells or blood endothelial cells (**highlighted**). Contrary, ECM or ECM-interaction genes found downregulated are more generally found in ECM of different tissues (**highlighted**). This goes in line with a disturbed ECM or ECM interaction in LECs as response of an impaired environment produced by Osr1 KO mesenchymal cells and a compensatory upregulation of vascular genes in LECs.

Downregulated genes in LECs from E13.5 *Osr1*$^{GCE/GCE}$ embryos:
Col16a1, ***Ddr1***, *Lama5*, *Lama2*, *Lama1*, ***Eln***, *Ncan*, ***Col11a1***, ***Col12a1***, *Lama3*, ***Tnc***, *Htra1*, *Fgf2*, ***Thbs1***, *Scube3*, *Scube1*, *Spp1*, *Itga11*, ***Adamts9***, ***Postn***, *Tgfb2*, *Vcam1*, *Tgfr1*, ***Col1a1***, ***Mmp11***, ***Col3a1***, *Vcan1*, ***Col1a2***, ***Lox***, *Loxl1*, ***Col5a1***, ***Col6a2***, ***Col6a1***, ***Mfap2***, ***Col8a2***, ***Col8a1***, ***Col6a3***, ***Col9a2***, ***Tgfbi***, ***Matn4*** *and* ***Ddr2***

Upregulated genes in LECs from E13.5 *Osr1*$^{GCE/GCE}$ embryos:
Col18a1, *Tgfb1*, ***Lama4***, *Adam10*, *F11r*, ***Icam1***, ***Icam2***, *Capns1*, *Flrt2*, *Ibsp*, *Sh3pxd2b*, *Adam12*, ***Kdr***, ***Itgav***, *Itga6*, *Tll1*, *Cd47 and* ***Itga9***.

In case the reviewer is referring to ECM terms obtained after GO analysis of genes found deregulated in Osr1+ mesenchymal cells from E13.5 *Osr1*$^{GCE/+}$ vs *Osr1*$^{GCE/GCE}$ embryos, we have run this GO analysis previously (Vallecillo-Garcia, Orgeur et al. 2017).

6. Figure 3D-G: Given the enrichment of GO terms associated with axon guidance and nervous system development, is innervation of the skin also affected in Osr1GCE/GCE embryos? One might expect this to be the case.

We thank the reviewer for raising this point. It has indeed been established that neurons guide the formation of arteries during embryogenesis and that neurons provide important VEGFC-processing enzymes crucial for lymphatic vasculature formation (Vaahtomeri, Karaman et al. 2017, Wang, Muhl et al. 2020). We agree that genes associated with axon guidance found after GO analysis in E13.5 LECs may reflect this. We, however, point out that we recapitulated the two major LEC defects (migration and distal cell proliferation) in isolated *in vitro* systems that exclude confounding effects as potentially mispatterned neurons. Furthermore, in our previous study investigating the role of motoneurons in lymph node initiation at the stage E13.5 (Vallecillo-Garcia, Orgeur et al. 2023), we did not observe major changes in motoneuron formation. Also, motoneurons of E14.5 hindlimbs stained by whole-mount immunofluorescence (Figure below) do not show major changes.

Nevertheless, an in-depth study would have to be performed to completely rule out this possibility, which is out of the scope of the present study.

© 2024. Published by The Company of Biologists under the terms of the Creative Commons Attribution License (https://creativecommons.org/licenses/by/4.0/).

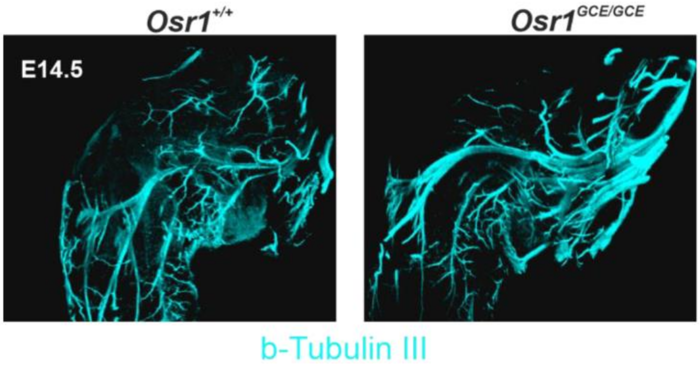

7. Could the authors postulate as to why genes important for lymphatic vascular development are upregulated in mutant embryos when lymphangiogenesis is affected?

We thank the reviewer for raising this intriguing aspect. We were initially puzzled by our LEC RNA-Seq results showing upregulation of many genes involved positively in lymphangiogenesis despite the clear lymphatic defects observed in *Osr1*-deficient embryos. We have initially interpreted this as compensatory upregulation, and have now strongly expanded this discussion:
"Alternatively, two important further aspects may separately or in combination play a role in this transcriptional upregulation. One aspect is the ECM and its mechanical properties sensed by LECs in *Osr1*$^{GCE/GCE}$ embryos. We have shown that Osr1+ cells are important producers of ECM proteins and that lack of *Osr1* leads to impaired ECM in muscle connective tissue (Vallecillo-Garcia, Orgeur et al. 2017), and in the skin of E14.5 embryos embedding lymphatic vasculature and capillaries (Figure 5 and S4). It has been demonstrated that the VEGFR3 tyrosine kinase function is highly dependent on mechanical forces (Galvagni, Pennacchini et al. 2010, Planas-Paz, Strilic et al. 2012, Baeyens, Nicoli et al. 2015) representing a crucial aspect in signaling pathway activation downstream of VEGFR3, even independent of VEGFC action. It was shown that reduced tissue stiffness leads to the upregulation of genes positively implicated in lymphatic vascular formation, e.g. *Flt4*, *Itga9* or *Hey1* (Frye, Taddei et al. 2018), which we found upregulated in LECs from E13.5 *Osr1*$^{GCE/GCE}$ embryos. Intriguingly, we have observed reduced tissue stiffness upon loss of *Osr1* in a model of skeletal muscle injury (Kotsaris, Qazi et al. 2023). Thus, the defective EMC we found in *Osr1*$^{GCE/GCE}$ embryos may lead to the transcriptional upregulation of genes involved in lymphangiogenesis in LECs. Another important aspect also coupled to the ECM component is fluid shear stress sensed by LECs. LECs are very sensitive to changes in shear stress and VEGFR3 builds a mechanosensory complex modulating different aspects of LEC behavior (Baeyens, Nicoli et al. 2015, Angeli and Lim 2023). *Osr1*$^{GCE/GCE}$ embryos show malformation in the septum primum (Wang, Lan et al. 2005) and we here found lymphatic vasculature defects; both aspects very likely affect the shear stress experienced by LECs in E13.5 *Osr1*^{GCEGCE} embryos. Thus, the combination of changes in fluid shear stress experienced by LECs on the luminal side and an impaired ECM and defective support of signaling molecules at the basal side could, in combination, explain upregulation of genes involved in lymphangiogenesis in LECs of E13.5 *Osr1*$^{GCE/GCE}$ embryos. Furthermore, *Vegfc*$^{+/-}$ heterozygous embryos showed only very subtle defects in embryonic lymphangiogenesis (Hagerling, Pollmann et al. 2013, Srinivasan, Escobedo et al. 2014). Altogether, this argues against reduced *Vegfc* expression in *Osr1*$^{GCE/GCE}$ cells as the primary explanation for the lymphatic vasculature defects observed in *Osr1* knockout embryos."

8. Figure 5: Inclusion of higher magnification images of the ECM components assessed in the skin of WT and GCE/GCE mice would be informative for the reader to gain deeper insight to the nature of the ECM defects. The orientation of the images shown with respect to the embryo should also be indicated.

We thank the reviewer for the advice. We have included in figure 5A and 5B higher magnification of the ECM markers COL12A1 and TNC showing ECM impairments in the skin of E14.5 *Osr1*$^{GCE/GCE}$ embryos. Besides, we have included arrows pointing to the orientation of lymphatic vessels growing towards the skin from the ventral to the dorsal side to allow orientation. The T represents Tips and points to the most dorsal lymphatic vasculature growing into the avascular midline in the middle of the back skin. Contrary, V represents ventral and shows established lymphatic vessels at the opposite side of the tips. For clarification, the representative images shown in figure 5A-C display

© 2024. Published by The Company of Biologists under the terms of the Creative Commons Attribution License (https://creativecommons.org/licenses/by/4.0/).

lymphatic vessels and the surrounding ECM of lymphatic vessels at the front of the migrating zone, whereas images from blood capillaries have been captured more ventrally. This notion has also been added to the manuscript.

9. Page 11: Fig Supp 4C, Figure 5D: Do the authors intend to differentiate between reduced accumulation of COL1 protein and impaired COL1 deposition? It's not clear whether the images shown represent this, or whether they simply illustrate reduced levels of COL1 in the vicinity of blood vessels.

We thank the reviewer for this clarification. Indeed, we cannot discriminate between impaired Collagen deposition versus reduced Collagen content. In figure 5D, we show an irregular COL1 staining compared to the smooth COL1 deposition in the basal lamina found in control embryos.

Besides, in figure S4C we have included the marker VEGFR3 to show that in contrast to blood capillaries forming lymphatic vasculature do not show a prominent basal lamina along the endothelial layer enriched in COL1 at this stage yet.

We have adapted the text as follows:
"Despite normal formation of dermal blood vessels observed in E14.5 *Osr1*$^{GCE/GCE}$ embryos (figure S2F), closer appreciation of the basal lamina in dermal blood capillaries showed an irregular, ruffled COL1 staining along the PECAM1+ endothelial layer (figure 5D). In contrast to blood capillaries, lymphatic vessels at this stage showed almost an absent COL1 staining along the VEGFR3^{high} endothelial layer (figure S4C)."

10. The claim that LECs positioned at the front of the migrating dermal vessels proliferate more than those positioned more laterally should be supported by quantification. Details regarding exactly where cell counts were performed need to be provided in the figure legend and the Methods. Figure 6E: n=4? If each colour represents a field from one embryo, the n should be 3 as currently shown. Additional details of the method used to quantify proliferation and how many cells were counted should be added to the Methods section. At present the data is not completely convincing. Likewise, the Methods would benefit from more detail describing the proliferation assays illustrated in Fig 6G/H. In which media were the cells cultured in Figure 6G? How many cells were counted? Panel G: what is the PROX1-negative, Ki67-positive staining in these panels if these represent LECs grown on de-cellularised matrix? Are total levels of VEGFC altered in GCE/GCE skin? Could migration assays be performed on the GCE/GCE compared to GCE/+ de-cellularised matrix?
Thank you for pointing out this lack of information making our results difficult to interpret. We point out that in figure 6E we have indeed performed such quantification. The left image shows an overview of whole-mount skin immunofluorescence images showing lymph vessels from the ventral side up to the distal dorsal tips. Right to that, the boxed ventral and dorsal tip regions are shown as magnifications, such images have been used for quantification (figure 6E 1-4). At the right side, we have included bar graphs with the quantification of PROX1+/KI67+ cells in these regions together with a small scheme at the right corner, again highlighting the anatomical locations used for quantification. To make this clearer, we have now added further details of the quantification performed in figures 6E-H to the methods section, including also a more detailed description of the *in vitro* experiments shown in figure 6F-H. The reviewer is right, in figure 6E, n=3, we apologize. We have adapted the methods related to the imaging quantification as follows:

Imaging

X-Gal staining of whole lymph nodes or the dermis of the ear were documented with a Zeiss SteREO Discovery V12 stereomicroscope. Confocal images of immunolabelled sections were taken using the confocal laser scanning microscope systems LSM710, LSM810 (Zeiss) or Leica DMi8 microscopes. Images were captured using Zen 2010 (Zeiss) and LAS Life System (Leica). For quantifications shown in figure 2A, at least 3 consecutive 100µm sections were quantified per embryo. In figure 2B, 4-5 regions along the rostro-caudal axis of the back from 4-5 consecutive 100µm sagittal sections were quantified per embryo. In figure 2D and 2E, lumbar and cervical region were quantified separately assessing the distance of the tips to the centre of the avascular midline in at least 20-30 lymphatic vessels per embryo. In figure 2G, 2G and S2F, branching points, vessel thickness and vessel density were quantified in 3 different regions of 1 mm^2 skin per embryo. In figure 2I, number of filopodia per PROX1+ LEC were quantified in 6 different regions at the tip of

© 2024. Published by The Company of Biologists under the terms of the Creative Commons Attribution License (https://creativecommons.org/licenses/by/4.0/).

the migrating zone per embryo. In figures 6E and 7F, PROX1+/KI67+ double positive cells were quantified in 3 different regions at the tip and 3 different regions at the ventral side per embryo. In figure 6G and 6H, Prox1^{High} cells were quantified per region, choosing 3-4 regions of 1 mm^2 per biological replicate (cells or medium derived from one embryo). In each region 500-20 cells per region were quantified. In figures 2A, 2B, 2D, 2E, 2G, 2H, 2I, S2F, 6E, 6G, 6H and 7F measurements from a single embryo are depicted in the same colour.

To the question, "In which media were the cells cultured in Figure 6G?", we have already described in the method part that LECs were culture on dECMs for 48h in LEC medium.

"To assess tdLECs proliferation cultures on dECM, they were cultured for 48 h in LECs medium."
"LEC medium that contains high-glucose DMEM, 20% FBS, 1% P/S solution, 10 µg/ml endothelial cell growth supplement (ECGS) (Thermo scientific), 50 µM 2-Mercaptoethanol, 1% Non-essential amino acids solution (Thermo scientific) and 50 µM for 4-7 days."

The reviewer also pointed to an important aspect of the LEC isolation with the question "what is the PROX1-negative, Ki67-positive staining in these panels if these represent LECs grown on de-cellularised matrix?". For *in vitro* experiments we isolated dermal LECs from adult mice as shown in figures S5E and F. Despite selecting CD31+/PDPN+ lymphatic endothelial cells via FACS, pdLECs grown in culture tend to display reduced PROX1 expression or lose PROX1 expression after several passages. In addition, few fibroblasts (PDPN+) could contaminate our pdLEC isolation. To standardize quantification in our *in vitro* experiments, we therefore decided to only quantify PROX1^{high} LECs. In our view, these cells most closely reflect in vivo LECs.

To the question, "Are total levels of VEGFC altered in GCE/GCE skin?", we have only accessed *Vegfc* transcripts in Osr1+ mesenchymal cells, BECs and LECs isolated from whole embryos. Only in *Osr1*$^{GCE/GCE}$ cells we observed a reduction in *Vegfc* (Figure 6B). As shown in Figure S5D, transcriptional analysis of E14.5 skin (Rezza, Wang et al. 2016) showed that melanocytes together with fibroblasts are the most important source of *Vegfc* in E14.5 skin. We have tried to measure VEGFC in whole tissue lysates from E11.5-E13.5 Osr1 embryos with the antibody (AF752, bio-techne) and in immunofluorescence showing only unfruitful results with high background staining of VEGFC in skeletal muscle tissue (figure below). To our knowledge the measurement of murine VEGFC using an antibody has been elusive and either human VEGFC or a VEGFC recombinant form or a *Vegfc*-reporter had to be used for this purpose in mice (Karkkainen, Haiko et al. 2004, Nurmi, Saharinen et al. 2015, Bui, Enis et al. 2016, Wang, Muhl et al. 2020). We think that conducting such experiments are out of the scope of this manuscript. We would like to point out that, as discussed in the manuscript, our evidence argues against a role of reduced *Vegfc* transcript expression in our model.

© 2024. Published by The Company of Biologists under the terms of the Creative Commons Attribution License (https://creativecommons.org/licenses/by/4.0/).

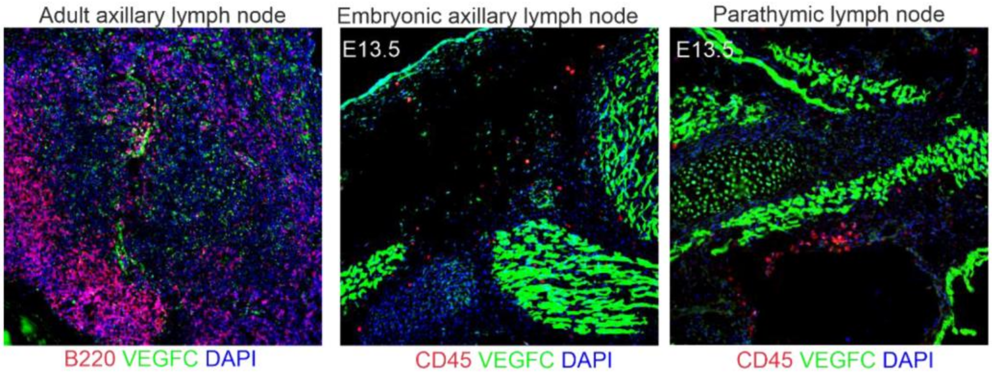

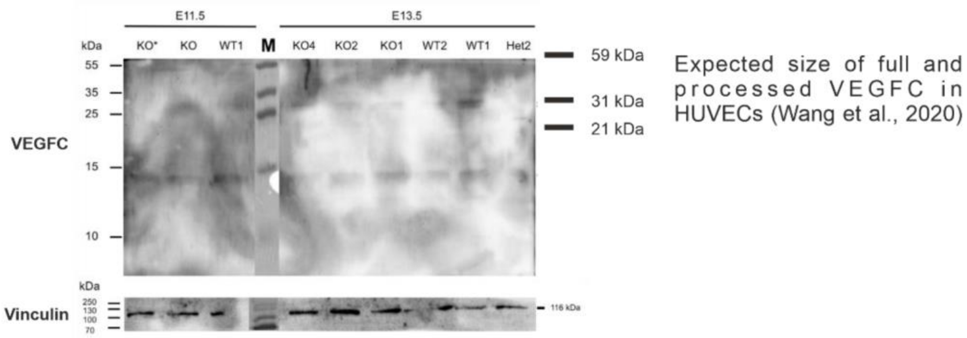

To the question, Could migration assays be performed on the GCE/GCE compared to GCE/+ de-cellularised matrix?. De-cellularized assays are quite challenging with loosely attached de-cellularized matrix to the surface after the treatment used for de-cellularization. In addition, LECs have to migrate directionally on the de-cellularized ECM in order to access migration. This excludes conventional scratch assays or the like; we were not able to identify an assay where LEC migration can be monitored on de-cellularized ECM without disrupting the de-cellularized ECM.

11. Figure 7B: The migration assays shown suggest a delay to migration promoted by GCE/GCE conditioned media. Is this predicted to be sufficient to account for reduced LEC migration observed in embryos? Would it be more relevant to look at the migration of LECs on Osr1-deficient mesenchymal cells?
We certainly cannot quantitatively infer from the *in vitro* scratch assay to the *in vivo* phenotype. Importantly, we do not state that migration is solely controlled by secreted signaling factors. Indeed, as mentioned above (comment 10) we are not able to assess the effect of *Osr1* cell-secreted ECM on migration. We have in fact addressed this issue in the discussion:
"However, the ECM and factors involved in endothelial-ECM interaction also play a role in controlling endothelial migration and stability (Levchenko, Aase et al. 2003, Aase, Ernkvist et al. 2007, Zhang, Zhang et al. 2021). Interestingly, *Amot* and genes encoding direct AMOT interactors such as *Kank1*, *Kank3* and *Flnc* (54) are downregulated in LECs of E13.5 *Osr1*$^{GCE/GCE}$ embryos (figure S7B). We also observed a reduced formation of filopodia at the tips of migrating LECs in E14.5 *Osr1*$^{GCE/GCE}$ embryos altogether suggesting a defective LEC-ECM interaction. We therefore cannot exclude that the altered ECM in *Osr1*$^{GCE/GCE}$ embryos may also in part contribute to impaired LEC migration. "

12. While the data shown in Figure 7E demonstrate a similar phenotype in CXCR4- deficient embryos to that observed in Osr1 mutant embryos, they do not definitively ascribe the phenotype observed in Osr1GCE/GCE mutants to a loss of CXCL12 in Osr1 deficient mesenchymal cells. This should be investigated using conditional mutant mouse lines, or the claim that CXCL12/CXCR4 signaling is required for LEC dorsal migration downstream of mesenchymal Osr1 should be toned down. The well established roles of CXCL12/CXCR4 signaling in blood vascular development mean that effects observed in the blood vasculature of CXCR4 KO mice may also impact the lymphatic phenotype. The blood vascular phenotype of CXCR4 KO mice should also be shown.

We agree with the reviewer that the phenotype observed in Osr1 KO embryos can´t be only ascribed to a defective CXCL12-CXCR4 signaling in these embryos.

© 2024. Published by The Company of Biologists under the terms of the Creative Commons Attribution License (https://creativecommons.org/licenses/by/4.0/).

Firstly, we only observe a reduction in *Cxcl12* in E13.5 *Osr1*$^{GCE/GCE}$ cells. Secondly, we hypothesize that a combination of signaling molecules produced by Osr1+ cells and the ECM synthesized by them are responsible for the defects in lymphatic vasculature in Osr1- deficient embryos. We have adapted our initial conclusion
"This suggests that in the mouse CXCL12/CXCR4 signaling is required for LEC dorsal migration downstream of mesenchymal *Osr1*." to
"This suggests that in the mouse CXCL12/CXCR4 signaling contribute to LEC dorsal migration downstream of mesenchymal *Osr1*."
The role of CXCL12/CXCR4 signaling in the formation of blood vasculature as well as the blood vascular phenotype of CXCR4 KO embryos have been well documented in the literature (Ara, Tokoyoda et al. 2005, Takabatake, Sugiyama et al. 2009, Li, Kohara et al. 2013, Li, Liu et al. 2021). In an initial step neural derived CXCL12 is essential for arterial differentiation and smooth muscle cell coverage. Studies performed in zebrafish have revealed that arterial endothelial cells and mural cells are a source of Cxcl12 supporting LEC migration via its receptor Cxcr4 (Cha, Fujita et al. 2012, Peng, Ando et al. 2022). Therefore, defects in arterial orientation or impairments in smooth muscle cell coverage observed in CXCR4 KO could influence the source of CXCL12 signals that is required for LEC migration later making difficult to separate between primary and secondary effects. However, we point out that CXCL12 and CXCR4 KOs did not show defects in vascular density of developing skin vasculature (Li, Kohara et al. 2013, Li, Liu et al. 2021). Consistently, whole-mount immunofluorescence of E14.5 skin did not show impairments in the capillary plexus of CXCR4^{KO} embryos (Figure below).

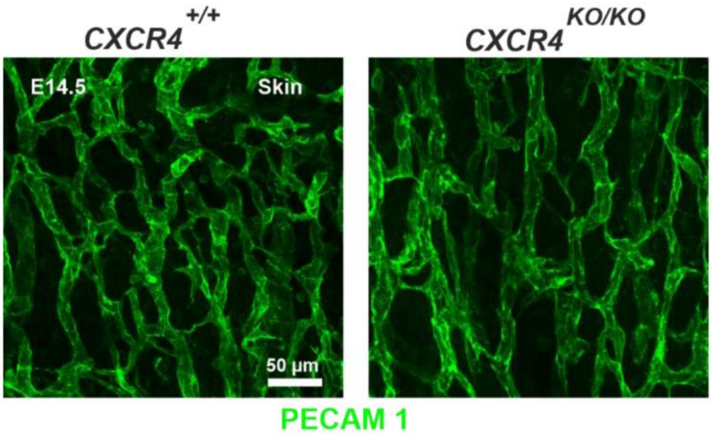

Reviewer 2 Advance Summary and Potential Significance to Field...

The coordination of lymphatic endothelial cell (LEC) migration in the 3D space of an embryo remains incompletely understood. Vallecillo-Garcia et. al. has characterised the role of Osr1+ mesenchymal cells in regulating LECs behaviours. They determined that Osr1+ cells appear on the LECs migratory path prior to the initiation of LEC migration. Furthermore, mice deficient in Osr1+ show defects in lymphatic vessel sprouting and morphology. Transcriptional analysis of LECs and Osr1+ mesenchymal cells identified ligand and receptor pairs that could explain the cellular interaction. As Osr1+ cells express Vegfc, the authors investigated VEGFC/VEGFR3 signalling in LECs from Osr1 KO embryos where they observed an increase in the expression of the target genes, suggesting that the interaction of Osr1+ cells with LECs is not a positive regulator of this signalling cascade. In addition, the authors showed that CXCR4 has similar phenotypes in migration but not the proliferation of LECs as the Osr1 KO embryo. They have shown that mesenchymal cells support LEC by maintaing the ECM scaffold. Together, the study concluded that Osr+ cells non-autonomously coordinate proliferation via ECM deposition and migration of LECs via CXCR4-CXCL12 signalling. This is an interesting study which provides significant insights into how LEC migration is coordinated by uncovering the interaction of Osr1+ mesenchymal cells with LECs; however, some points require further clarification before publication.

© 2024. Published by The Company of Biologists under the terms of the Creative Commons Attribution License (https://creativecommons.org/licenses/by/4.0/).

Reviewer 2 Comments for the Author...

-The main observation of the defects in lymphatic vessels is done at 14.5, when the embryos show profound oedema. Swelling of the tissues and changes in the interstitial pressure by itself can induce lymphatic phenotypes (Urner et al., 2018 EMBO J). It would be important to confirm which of the observed phenotypes are due to Osr1 KO and which due to oedema. A suggestion would be to assess the proliferation and migration phenotypes prior to oedema formation. In addition, quantification of pTD LECs would be informative.

We thank the reviewer for highlighting this important point and the useful recommended literature. In order to assess migration phenotypes prior oedema formation, we have now analyzed **a)** the amount of PROX1+ cells inside and outside of the cardinal vein in E11.5 *Osr1*$^{+/+}$ and *Osr1*$^{GCE/GCE}$ KO embryos and **b)** the amount of VEGFR3+ lymphatic vasculature formed distally from the pTD close to the back skin at E12.5 as a readout for early LEC migration. At E11.5, we did not observe a significant reduction of LECs migrating outside of the cardinal vein in Osr1-deficient embryos (Figure 2A). After the analysis of VEGFR3+ signals in 100 µm thick sagittal sections of E12.5 embryos, we identified a migration defect already at this stage in *Osr1*$^{GCE/GCE}$ embryos (Figure 2B). Of note, at E12.5, back oedema it is not visible in *Osr1*$^{GCE/GCE}$ embryos.

-The authors noted puzzling results wherein Vegfc is downregulated in Osr1 KO mesenchymal cells, yet the targets of VEGFC/VEGFR3 are upregulated at the transcriptional level in LECs. It would be beneficial to include a discussion of what these results indicate with their potential biological implications. In addition, to better understand these phenotypes, it would be beneficial to supplement these observations with an analysis of the levels of pERK or pAKT in LECs.

We agree with the reviewer that transcriptional downregulation of *Vegfc* observed in *Osr1*$^{GCE/GCE}$ mesenchymal cells and upregulation of *Flt4* and other genes involved in the VEGFC/VEGFR3 signaling pathway obtained after E13.5 LEC RNA-Seq are not going in line with the typical phenotype observed after genetic disruption of VEGFC/VEGFR3 signaling. However, it has been demonstrated that the VEGFR3 tyrosine kinase function is highly dependent on mechanical forces (Galvagni, Pennacchini et al. 2010, Planas-Paz, Strilic et al. 2012, Baeyens, Nicoli et al. 2015) resulting a crucial aspect in signaling pathway activation downstream of VEGFR3 even independently of VEGFC action. Thus, LEC interaction with the surrounding ECM via integrins is critical for VEGFR3 activation (Planas- Paz, Strilic et al. 2012, Baeyens, Nicoli et al. 2015, Urner, Planas-Paz et al. 2019). We have shown that Osr1+ cells are important producer of ECM proteins and that lack of *Osr1* leads to impaired ECM in muscle connective tissue (Vallecillo-Garcia, Orgeur et al. 2017) and in the skin of E14.5 embryos embedding lymphatic vasculature and capillaries (Figure 5 and S4). In addition, we have shown that in E13.5 LEC transcriptomic analyses ECM-receptor interaction terms are enriched within deregulated genes (Figure 3F) with a higher proportion of genes being downregulated in LECs from *Osr1*$^{GCE/GCE}$ embryos (Figure S3C). Thus, a possible scenario in *Osr1*-deficient embryos may be represented by a generalized impaired ECM produced by Osr1+ mesenchymal cells that influences LEC-behavior and final lymphatic vasculature formation via mechanical stabilization of ECM-LEC interactions even at expenses of transcriptional upregulation of *Flt4* in LECs. Alternatively, an impaired ECM may in turn influence the capacity of lymphatic vasculature to maintain fluid pressure and influencing downstream VEGFR3 pathway independently of VEGFC.
We have included this idea and adapted the discussion as follows:

"We found that Osr1+ cells are a source of *Vegfc* in embryonic tissues. Interestingly, *Vegfc* expression at early stages of development was higher in endothelial cells (Cao, Spielmann et al. 2019) while at E13.5 Osr1+ mesenchymal cells and BECs expressed similar amounts of *Vegfc*. Despite *Vegfc* downregulation observed only in E13.5 *Osr1*$^{GCE/GCE}$ mesenchymal cells, the LEC transcriptome data suggested that embryonic E13.5 LECs show even increased expression of genes involved in the VEGFC-VEGFR3 cascade, at least when assessed at the transcriptional level. This might represent a compensatory mechanism in line with the transcriptional upregulation of ECM components, ECM-interaction genes and other genes positively involved in vascular development, as observed in GO analysis of E13.5 LECs from *Osr1*$^{GCE/GCE}$ embryos. Alternatively, two important further aspects may separately or in combination play a role in this transcriptional upregulation. One aspect is the ECM and its mechanical properties sensed by LECs in *Osr1*$^{GCE/GCE}$

© 2024. Published by The Company of Biologists under the terms of the Creative Commons Attribution License (https://creativecommons.org/licenses/by/4.0/).

embryos. We have shown that Osr1+ cells are important producers of ECM proteins and that lack of *Osr1* leads to impaired ECM in muscle connective tissue (Vallecillo-Garcia, Orgeur et al. 2017), and in the skin of E14.5 embryos embedding lymphatic vasculature and capillaries (Figure 5 and S4). It has been demonstrated that the VEGFR3 tyrosine kinase function is highly dependent on mechanical forces (Galvagni, Pennacchini et al. 2010, Planas-Paz, Strilic et al. 2012, Baeyens, Nicoli et al. 2015) representing a crucial aspect in signaling pathway activation downstream of VEGFR3, even independent of VEGFC action. It was shown that reduced tissue stiffness leads to the upregulation of genes positively implicated in lymphatic vascular formation, e.g. *Flt4*, *Itga9* or *Hey1* (Frye, Taddei et al. 2018), which we found upregulated in LECs from E13.5 *Osr1*$^{GCE/GCE}$ embryos. Intriguingly, we have observed reduced tissue stiffness upon loss of *Osr1* in a model of skeletal muscle injury (Kotsaris, Qazi et al. 2023). Thus, the defective EMC we found in *Osr1*$^{GCE/GCE}$ embryos may lead to the transcriptional upregulation of genes involved in lymphangiogenesis in LECs. Another important aspect also coupled to the ECM component is fluid shear stress sensed by LECs. LECs are very sensitive to changes in shear stress and VEGFR3 builds a mechanosensory complex modulating different aspects of LEC behavior (Baeyens, Nicoli et al. 2015, Angeli and Lim 2023). *Osr1*$^{GCE/GCE}$ embryos show malformation in the septum primum (Wang, Lan et al. 2005) and we here found lymphatic vasculature defects; both aspects very likely affect the shear stress experienced by LECs in E13.5 *Osr1*^{GCEGCE} embryos. Thus, the combination of changes in fluid shear stress experienced by LECs on the luminal side and an impaired ECM and defective support of signaling molecules at the basal side could, in combination, explain upregulation of genes involved in lymphangiogenesis in LECs of E13.5 *Osr1*$^{GCE/GCE}$ embryos. Furthermore, *Vegfc*$^{+/-}$ heterozygous embryos showed only very subtle defects in embryonic lymphangiogenesis (Hagerling, Pollmann et al. 2013, Srinivasan, Escobedo et al. 2014). Altogether, this argues against reduced *Vegfc* expression in *Osr1*$^{GCE/GCE}$ cells as the primary explanation for the lymphatic vasculature defects observed in *Osr1* knockout embryos."

We thank the reviewer for the suggestion related the analysis of pERK or pAKT levels in LECs to support the idea that upregulation of *Flt4* observed only transcriptionally in LECs from E13.5 *Osr1*$^{GCE/GCE}$ embryos is leading to an activation downstream of target genes. Recent studies elegantly shown that ERK activation in zebrafish robustly promotes LEC proliferation and LEC migration (Peng, Ando et al. 2022). By investigating the influence of VEGFC-VEGFR3 and CXCL12-CXCR4 signaling separately or in conjunction in the context of LEC migration, their data indicated that ERK activation may occur mainly via VEGFC-VEGFR3 rather than CXCL12-CXCR4 signaling promoting LEC migration. Since our results showed defects in migration before the appearance of back edema at E12.5 and in the skin of E14.5 *Osr1*$^{GCE/GCE}$ embryos together with decreased proliferation of PROX1+ LECs at the front of the migrating zone, we were not expecting that *Flt4* upregulation in LECs from E13.5 *Osr1*$^{GCE/GCE}$ embryos will lead to an effective upregulation of pERK protein downstream of VEGFR3. In addition, it was previously shown that in mice at E12.5 only a small proportion of LECs activate ERK in the pTD and in the skin of E14.5 embryos (Fatima, Wang et al. 2016). If ERK is also activated in LEC during their migration in murine development remains to be clarified. Therefore, we have assessed the expression of pERK protein in of 100µm thick sagittal sections and in skin LECs at the migrating front of E14.5 *Osr1*^{Controls} and *Osr1*$^{GCE/GCE}$ embryos (figure below). We were not able to detect positive nuclear signals of pERK staining neither in dorsally migrating VEGFR3+ LECs at E12.5 (Figure below, A-D) nor in PROX1+ LECs at the migrating zone in E14.5 embryos (Figure below, E, F). Importantly, we did not detect an increase of pERK signals in LECs of *Osr1*$^{GCE/GCE}$ embryos. Of note, pERK can be detected prominently in non-endothelial cells close to the back skin at E12.5 (Figure below, C and D) or in the dermis at E14.5 (Figure below, E, F). A more comprehensive analysis of ERK activation in murine LECs has to be performed to address the role of ERK in migrating LECs. Based on our collective evidence, we contend that the reduced expression of *Vegfc* in Osr1+ cells does not contribute to the defects observed in Osr1 knockout embryos. Therefore, we believe that a deeper analysis of VEGFC/VEGFR3 signaling falls outside the scope of this manuscript.

© 2024. Published by The Company of Biologists under the terms of the Creative Commons Attribution License (https://creativecommons.org/licenses/by/4.0/).

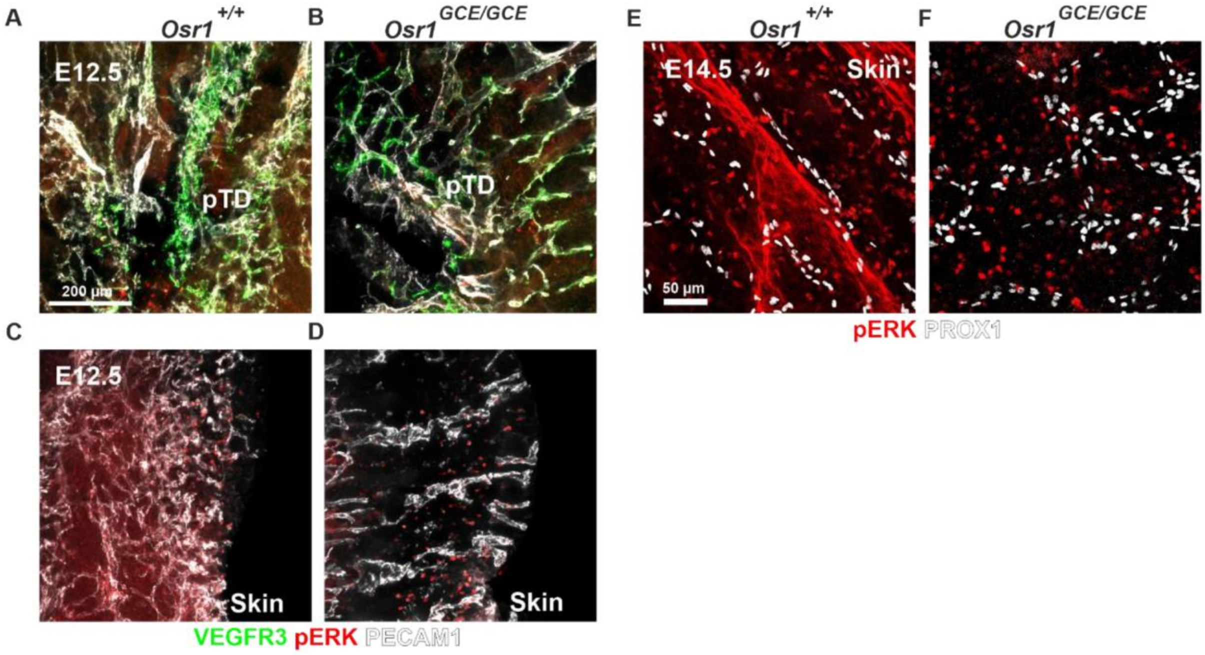

-The authors conducted elegant experiments using decellularised ECM or cell-conditioned media to assess how Osr1+ mesenchymal cells regulate LEC proliferation vs. migration. To further understand whether VEGFC is indeed secreted from Osr1+ cells and if this is inhibited in KO cells performing western blot analysis for VEGFC on the culture medium. Such an experiment would also elucidate whether Osr1 cells exclusively secrete VEGFC and if the ECM they produce is necessary for VEGFC activation/processing.

We thank the reviewer for acknowledging the challenging experiments conducted on decellularized ECM produced by E13.5 Osr1+ mesenchymal cells and the media produced by these cells. The production of VEGFC and their final active form has interested many scientist in the field of lymphangiogenesis. Despite the well-studied function of VEGR3 during development and adult lymphatic vessel formation, way less is known about the control of the production of VEGFC final active form. Our data shows that Osr1+ mesenchymal cells express *Vegfc* and the VEGFC-processing enzymes (*Cceb1*, *Adamts3* and *Adamts14*). We have also confirmed that in the E13.5 embryo, vein endothelial cells and Osr1+ mesenchymal cells produce significant amounts of *Vegfc (Figures 6A, S5A-C)*. In addition, transcriptional analysis performed from the E14.5 skin (Rezza, Wang et al. 2016) include dermal melanocytes (Figure S5D) as *Vegfc* producers. Therefore, Osr1+ mesenchymal cells do not exclusively express *Vegfc* in the embryo and several cell types may contribute to VEGFC expression. That may hold true also for the processing enzymes leading to the final active form of VEGFC. An additional level of regulation may be represented by the accessibility of VEGFC to LECs via the ECM. Changes in ECM organization and structure may sequester or may more accessible VEGFC and thus influencing LEC behavior via VEGFR3.
We think that the expression of *Vegfc* by Osr1+ cells does represent an important source of VEGFC. As the reviewer suggested assessing via western blot if pre-VEGFC or its intermediate are found in Osr1+ cells may represent an important experiment. We have tried previously to measure VEGFC in whole tissue lysates from E11.5-E13.5 Osr1 embryos with the antibody (AF752, bio-techne) and in immunofluorescence showing only unfruitful results with high background staining by the anti-VEGFC antibody in skeletal muscle tissue (figure below). To our knowledge, the measurement of murine VEGFC using an antibody has been elusive and either human VEGFC or a VEGFC recombinant form or a *Vegfc*- reporter had to be used for this purpose in mice (Karkkainen, Haiko et al. 2004, Nurmi, Saharinen et al. 2015, Bui, Enis et al. 2016, Wang, Muhl et al. 2020). We think that conducting such experiments are out of the scope of this manuscript.

© 2024. Published by The Company of Biologists under the terms of the Creative Commons Attribution License (https://creativecommons.org/licenses/by/4.0/).

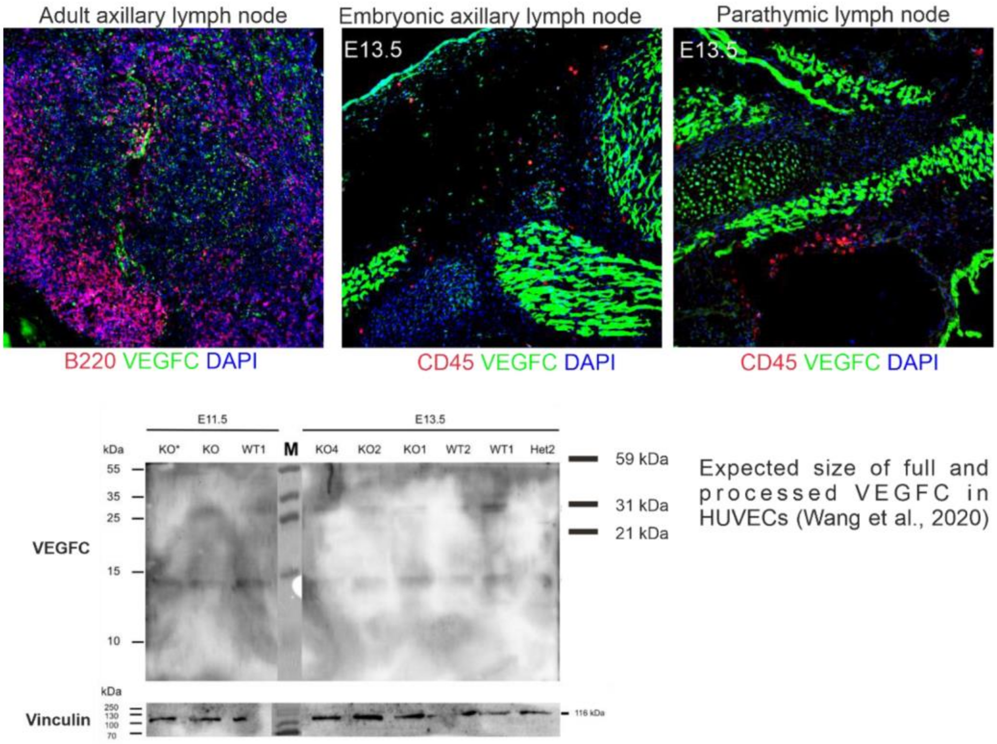

The authors state that there is a close association between the Osr1+ mesenchymal cells and LEC. Do these cells make direct contact or LECs only need to sense the ECM?
Providing a more detailed and higher-resolution analysis of the interaction could be beneficial.

This is an interesting question. We have shown that from E11.5 until E14.5 Osr1+ cells are in close contact with migrating LECs and established lymphatic vasculature (Figures 1 and S1). However, based on our previous knowledge such as transcriptomic data of Osr1+ cells, we assumed that during these stages Osr1+ cells are producing and remodeling a specific ECM that may influence LEC behavior. We hypothesized that Osr1+ cells arriving earlier than migrating LECs establish thus may control lymphatic vessel formation via the production of ECM and signaling molecules. We point out that the *ex vivo* assays used, however, do not contain Osr1+ cells, but only conditioned medium or ECM, still in essence recapitulating the observed defects in proliferation and migration. However, certainly, direct cell-cell contacts in addition might play a role *in vivo*. We understand that an in- depth analysis of Osr1+ mesenchymal and LEC cellular interactions with high resolution imaging analysis of subcellular components and an analysis of molecular players involved in those interactions are definitely of interest here but out of the scope of the manuscript.

It has been shown that LEC clusters form in the mid-line and ahead of LEC migrating front in the back skin (Martinez-Corral et al, 2015 Circ Res., Pichol-Thievend et al, 2018 Development). Do the Osr1+cell associate with these LEC clusters? Could they serve as an indication of where these clusters will form?

We thank the reviewer for raising this interesting point. In figures 1D and 1E, we did not find a preferential accumulation of lineage E11.5 Osr1+ cells at the tips of new forming PROX1+ clusters. We have also performed whole-mount immunofluorescence of E14.5 $Osr1^{GCE/+}$ skin using eGFP as a reporter for Osr1+ cells and PROX1 and CD31 to identify lymphatic structures and blood capillaries. In this image (below), *Osr1* expression is found distributed from the ventral part of established lymphatic vasculature to the avascular midline in the dorsal part. We did not observe a differential accumulation of Osr1+ cells close to the appearance of LEC clusters ahead of the migrating front compare to other regions in the avascular midline (Figure below, arrowhead).

© 2024. Published by The Company of Biologists under the terms of the Creative Commons Attribution License (https://creativecommons.org/licenses/by/4.0/).

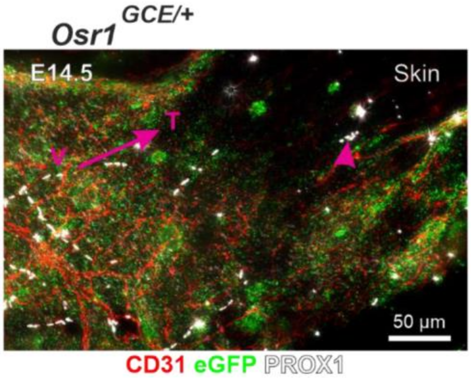

I would like to compliment the authors on the images in the paper, particularly the figure S2E, as well as the whole back skin overview in Figure S2C helps to appreciate the overall phenotype.
We thank the reviewer.

Minor

Figure 3H it would be informative to expand this map with additional markers for LEC vs BEC. This would further validate the correct cell type enrichment in LECs.

Does the reviewer rather refer to Figure 3B and expand thus the markers used to confirm LEC enrichment? In Figure 3H we compare the expression of several genes involved positively in LEC development in LECs FACS-isolated from E13.5 *Osr1*^{controls} and *Osr1*$^{GCE/GCE}$ embryos. Since Figure 3H shows normalized values to compare the expression within LECs, it cannot validate an enrichment of specific genes in LECs vs. other cell types.
In Figure 3B, we used the expression of *Prox1*, *Flt4* and *Ccl21a* to validate LEC enrichment. In the embryo, *Prox1* is also expressed in hepatocytes (which certainly do not play a role here) and *Flt4* is expressed at lower levels in BECs as shown in Figure 3B. We have observed previously that *Ccl21a* is a very specific marker for LECs in the embryo as shown in Vallecillo-Garcia et al., 2023 Figures 7A and S7A. Our RTqPCR results in Figure 3B confirmed these results at RNA level. In addition, we have now included a table (supplementary table 1) comparing TPM values after RNAseq analysis that includes more LEC, BEC and mesenchymal markers and confirms high expression of lymphatic endothelial markers in comparison to blood endothelial and mesenchymal markers.

Please clarify this sentence: "Contradictory to the notion that reduced Vegfc in mesenchymal cells may be responsible for the lymphatic vessel defects observed in Osr1 deficient embryos, we observed an increase in Flt4 expression in LECs of E13.5 Osr1GCE/GCE embryos (figure 3F)" for better comprehension.

Here, we address at the first glance contradictory results from downregulation *Vegfc* measured in E13.5 *Osr1*$^{GCE/GCE}$ cells and the upregulation of *Flt4* and other VEGFC/VEGFR3 related genes in LECs from *Osr1*$^{GCE/GCE}$ embryos (Figure 6C).
To clarify this section, we have adapted the text as follows:

"Contradictory to the notion that reduced *Vegfc* in mesenchymal cells may cause a downregulation of *Flt4* in LECs, we observed an increase in *Flt4* expression in LECs of E13.5 *Osr1*$^{GCE/GCE}$ embryos together with other genes involved in the VEGFR3-signaling axis such as *Hhex*, *Maf*, or *Egr1* (Shin, Huggenberger and Detmar 2008, Koltowska, Paterson et al. 2015, Dieterich, Ducoli et al. 2017, Gauvrit, Villasenor et al. 2018) (figure 6C)"

One could consider using "Zebrafish" instead of "zebra fish" to align with the common terminology.

We thank the reviewer for the recommendation. We have adapted the text accordingly.

<u>Literature:</u>

© 2024. Published by The Company of Biologists under the terms of the Creative Commons Attribution License (https://creativecommons.org/licenses/by/4.0/).

Aase, K., M. Ernkvist, L. Ebarasi, L. Jakobsson, A. Majumdar, C. Yi, O. Birot, Y. Ming, A. Kvanta, D. Edholm, P. Aspenstrom, J. Kissil, L. Claesson-Welsh, A. Shimono and L. Holmgren (2007). "Angiomotin regulates endothelial cell migration during embryonic angiogenesis." Genes Dev **21**(16): 2055-2068.
Angeli, V. and H. Y. Lim (2023). "Biomechanical control of lymphatic vessel physiology and functions." Cell Mol Immunol **20**(9): 1051-1062.
Ara, T., K. Tokoyoda, R. Okamoto, P. A. Koni and T. Nagasawa (2005). "The role of CXCL12 in the organ-specific process of artery formation." Blood **105**(8): 3155-3161.
Baeyens, N., S. Nicoli, B. G. Coon, T. D. Ross, K. Van den Dries, J. Han, H. M. Lauridsen, C. O. Mejean, A. Eichmann, J. L. Thomas, J. D. Humphrey and M. A. Schwartz (2015). "Vascular remodeling is governed by a VEGFR3-dependent fluid shear stress set point." Elife **4**.
Bui, H. M., D. Enis, M. R. Robciuc, H. J. Nurmi, J. Cohen, M. Chen, Y. Yang, V. Dhillon, K. Johnson, H. Zhang, R. Kirkpatrick, E. Traxler, A. Anisimov, K. Alitalo and M. L. Kahn (2016). "Proteolytic activation defines distinct lymphangiogenic mechanisms for VEGFC and VEGFD." J Clin Invest **126**(6): 2167- 2180.
Cao, J., M. Spielmann, X. Qiu, X. Huang, D. M. Ibrahim, A. J. Hill, F. Zhang, S. Mundlos, L. Christiansen, F. J. Steemers, C. Trapnell and J. Shendure (2019). "The single-cell transcriptional landscape of mammalian organogenesis." Nature **566**(7745): 496-502.
Cha, Y. R., M. Fujita, M. Butler, S. Isogai, E. Kochhan, A. F. Siekmann and B. M. Weinstein (2012). "Chemokine signaling directs trunk lymphatic network formation along the preexisting blood vasculature." Dev Cell **22**(4): 824-836.
Dieterich, L. C., L. Ducoli, J. W. Shin and M. Detmar (2017). "Distinct transcriptional responses of lymphatic endothelial cells to VEGFR-3 and VEGFR-2 stimulation." Sci Data **4**: 170106.
Fatima, A., Y. Wang, Y. Uchida, P. Norden, T. Liu, A. Culver, W. H. Dietz, F. Culver, M. Millay, Y. S. Mukouyama and T. Kume (2016). "Foxc1 and Foxc2 deletion causes abnormal lymphangiogenesis and correlates with ERK hyperactivation." J Clin Invest **126**(7): 2437-2451.
Frye, M., A. Taddei, C. Dierkes, I. Martinez-Corral, M. Fielden, H. Ortsater, J. Kazenwadel, D. P. Calado, P. Ostergaard, M. Salminen, L. He, N. L. Harvey, F. Kiefer and T. Makinen (2018). "Matrix stiffness controls lymphatic vessel formation through regulation of a GATA2-dependent transcriptional program." Nat Commun **9**(1): 1511.
Galvagni, F., S. Pennacchini, A. Salameh, M. Rocchigiani, F. Neri, M. Orlandini, F. Petraglia, S. Gotta, G. L. Sardone, G. Matteucci, G. C. Terstappen and S. Oliviero (2010). "Endothelial cell adhesion to the extracellular matrix induces c-Src-dependent VEGFR-3 phosphorylation without the activation of the receptor intrinsic kinase activity." Circ Res **106**(12): 1839-1848.
Gauvrit, S., A. Villasenor, B. Strilic, P. Kitchen, M. M. Collins, R. Marin-Juez, S. Guenther, H. M. Maischein, N. Fukuda, M. A. Canham, J. M. Brickman, C. W. Bogue, P. S. Jayaraman and D. Y. R. Stainier (2018). "HHEX is a transcriptional regulator of the VEGFC/FLT4/PROX1 signaling axis during vascular development." Nat Commun **9**(1): 2704.
Hagerling, R., C. Pollmann, M. Andreas, C. Schmidt, H. Nurmi, R. H. Adams, K. Alitalo, V. Andresen, S. Schulte-Merker and F. Kiefer (2013). "A novel multistep mechanism for initial lymphangiogenesis in mouse embryos based on ultramicroscopy." EMBO J **32**(5): 629-644.
Karkkainen, M. J., P. Haiko, K. Sainio, J. Partanen, J. Taipale, T. V. Petrova, M. Jeltsch, D. G. Jackson, M. Talikka, H. Rauvala, C. Betsholtz and K. Alitalo (2004). "Vascular endothelial growth factor C is required for sprouting of the first lymphatic vessels from embryonic veins." Nat Immunol **5**(1): 74-80.
Koltowska, K., S. Paterson, N. I. Bower, G. J. Baillie, A. K. Lagendijk, J. W. Astin, H. Chen, M. Francois, P. S. Crosier, R. J. Taft, C. Simons, K. A. Smith and B. M. Hogan (2015). "mafba is a downstream transcriptional effector of Vegfc signaling essential for embryonic lymphangiogenesis in zebrafish." Genes Dev **29**(15): 1618-1630.
Kotsaris, G., T. H. Qazi, C. H. Bucher, H. Zahid, S. Pohle-Kronawitter, V. Ugorets, W. Jarassier, S. Borno, B. Timmermann, C. Giesecke-Thiel, A. N. Economides, F. Le Grand, P. Vallecillo-Garcia, P. Knaus, S. Geissler and S. Stricker (2023). "Odd skipped-related 1 controls the pro-regenerative response of fibro-adipogenic progenitors." NPJ Regen Med **8**(1): 19.
Levchenko, T., K. Aase, B. Troyanovsky, A. Bratt and L. Holmgren (2003). "Loss of responsiveness to chemotactic factors by deletion of the C-terminal protein interaction site of angiomotin." J Cell Sci **116**(Pt 18): 3803-3810.
Li, W., H. Kohara, Y. Uchida, J. M. James, K. Soneji, D. G. Cronshaw, Y. R. Zou, T. Nagasawa and Y. S. Mukouyama (2013). "Peripheral nerve-derived CXCL12 and VEGF-A regulate the patterning of arterial vessel branching in developing limb skin." Dev Cell **24**(4): 359-371.

© 2024. Published by The Company of Biologists under the terms of the Creative Commons Attribution License (https://creativecommons.org/licenses/by/4.0/).

Li, W., C. Liu, N. Burns, J. Hayashi, A. Yoshida, A. Sajja, S. Gonzalez-Hernandez, J. L. Gao, P. M. Murphy, Y. Kubota, Y. R. Zou, T. Nagasawa and Y. S. Mukouyama (2021). "Alterations in the spatiotemporal expression of the chemokine receptor CXCR4 in endothelial cells cause failure of hierarchical vascular branching." Dev Biol **477**: 70-84.
Nurmi, H., P. Saharinen, G. Zarkada, W. Zheng, M. R. Robciuc and K. Alitalo (2015). "VEGF-C is required for intestinal lymphatic vessel maintenance and lipid absorption." EMBO Mol Med **7**(11): 1418-1425.
Peng, D., K. Ando, M. Hussmann, M. Gloger, R. Skoczylas, N. Mochizuki, C. Betsholtz, S. Fukuhara, S. Schulte-Merker, N. D. Lawson and K. Koltowska (2022). "Proper migration of lymphatic endothelial cells requires survival and guidance cues from arterial mural cells." Elife **11**.
Planas-Paz, L., B. Strilic, A. Goedecke, G. Breier, R. Fassler and E. Lammert (2012). "Mechanoinduction of lymph vessel expansion." EMBO J **31**(4): 788-804.
Rezza, A., Z. Wang, R. Sennett, W. Qiao, D. Wang, N. Heitman, K. W. Mok, C. Clavel, R. Yi, P. Zandstra, A. Ma'ayan and M. Rendl (2016). "Signaling Networks among Stem Cell Precursors, Transit-Amplifying Progenitors, and their Niche in Developing Hair Follicles." Cell Rep **14**(12): 3001-3018.
Shin, J. W., R. Huggenberger and M. Detmar (2008). "Transcriptional profiling of VEGF-A and VEGF-C target genes in lymphatic endothelium reveals endothelial-specific molecule-1 as a novel mediator of lymphangiogenesis." Blood **112**(6): 2318-2326.
Srinivasan, R. S., N. Escobedo, Y. Yang, A. Interiano, M. E. Dillard, D. Finkelstein, S. Mukatira, H. J. Gil, H. Nurmi, K. Alitalo and G. Oliver (2014). "The Prox1-Vegfr3 feedback loop maintains the identity and the number of lymphatic endothelial cell progenitors." Genes Dev **28**(19): 2175-2187.
Takabatake, Y., T. Sugiyama, H. Kohara, T. Matsusaka, H. Kurihara, P. A. Koni, Y. Nagasawa, T. Hamano, I. Matsui, N. Kawada, E. Imai, T. Nagasawa, H. Rakugi and Y. Isaka (2009). "The CXCL12 (SDF- 1)/CXCR4 axis is essential for the development of renal vasculature." J Am Soc Nephrol **20**(8): 1714- 1723.
Urner, S., L. Planas-Paz, L. S. Hilger, C. Henning, A. Branopolski, M. Kelly-Goss, L. Stanczuk, B. Pitter, E. Montanez, S. M. Peirce, T. Makinen and E. Lammert (2019). "Identification of ILK as a critical regulator of VEGFR3 signalling and lymphatic vascular growth." EMBO J **38**(2).
Vaahtomeri, K., S. Karaman, T. Makinen and K. Alitalo (2017). "Lymphangiogenesis guidance by paracrine and pericellular factors." Genes Dev **31**(16): 1615-1634.
Vallecillo-Garcia, P., M. Orgeur, G. Comai, S. Poehle-Kronnawitter, C. Fischer, M. Gloger, C. E. Dumas, C. Giesecke-Thiel, S. Sauer, S. Tajbakhsh, U. E. Hopken and S. Stricker (2023). "A local subset of mesenchymal cells expressing the transcription factor Osr1 orchestrates lymph node initiation." Immunity **56**(6): 1204-1219 e1208.
Vallecillo-Garcia, P., M. Orgeur, S. Vom Hofe-Schneider, J. Stumm, V. Kappert, D. M. Ibrahim, S. T. Borno, S. Hayashi, F. Relaix, K. Hildebrandt, G. Sengle, M. Koch, B. Timmermann, G. Marazzi, D. A. Sassoon, D. Duprez and S. Stricker (2017). "Odd skipped-related 1 identifies a population of embryonic fibro-adipogenic progenitors regulating myogenesis during limb development." Nat Commun **8**(1): 1218.
Wang, G., L. Muhl, Y. Padberg, L. Dupont, J. Peterson-Maduro, M. Stehling, F. le Noble, A. Colige, C. Betsholtz, S. Schulte-Merker and A. van Impel (2020). "Specific fibroblast subpopulations and neuronal structures provide local sources of Vegfc-processing components during zebrafish lymphangiogenesis." Nat Commun **11**(1): 2724.
Wang, Q., Y. Lan, E. S. Cho, K. M. Maltby and R. Jiang (2005). "Odd-skipped related 1 (Odd 1) is an essential regulator of heart and urogenital development." Dev Biol **288**(2): 582-594.
Zhang, Y., Y. Zhang, S. Kameishi, G. Barutello, Y. Zheng, N. P. Tobin, J. Nicosia, K. Hennig, D. K. Chiu,
M. Balland, T. H. Barker, F. Cavallo and L. Holmgren (2021). "The Amot/integrin protein complex transmits mechanical forces required for vascular expansion." Cell Rep **36**(8): 109616.

Second decision letter

MS ID#: DEVELOP/2024/202747

MS TITLE: Mesenchymal Osr1+ cells regulate embryonic lymphatic vessel formation

© 2024. Published by The Company of Biologists under the terms of the Creative Commons Attribution License (https://creativecommons.org/licenses/by/4.0/).

AUTHORS: Pedro Vallecillo Garcia, Mira Nicola Kuehnlein, Mickael Orgeur, Nils Rouven Hansmeier, Georgios Kotsaris, Bernd Timmermann, Claudia Giesecke-Thiel, Rene Haegerling, Sigmar Stricker, and Zarah Meisen

I have now received all the referees reports on the above manuscript, and have reached a decision. The referees' comments are appended below, or you can access them online: please go to BenchPress and click on the 'Manuscripts with Decisions' queue in the Author Area.

The overall evaluation is positive and we would like to publish a revised manuscript in Development, provided that the referees' comments can be satisfactorily addressed. Please attend to all of the reviewers' comments in your revised manuscript and detail them in your point-by-point response. If you do not agree with any of their criticisms or suggestions explain clearly why this is so. If it would be helpful, you are welcome to contact us to discuss your revision in greater detail. Please send us a point-by-point response indicating your plans for addressing the referees' comments, and we will look over this and provide further guidance.

Reviewer 1

Advance summary and potential significance to field

The authors have addressed the majority of comments/concerns of the reviewers particularly with regard to providing additional quantification of lymphatic parameters, and the revised manuscript is improved as a result. I agree that some of the experiments that would provide greater insight to the mechanisms by which Osr1-positive mesenchymal cells regulate lymphatic vascular development are currently hampered by technical limitations or the availability of reliable reagents. While addressing some of these mechanisms would provide further depth to this study, the revised manuscript contributes important new knowledge regarding a novel role for mesenchymal cells in the regulation of extracellular matrix deposition/remodelling as is important for lymphatic vessel growth and migration.

Comments for the author

A few minor comments to be addressed:
This reviewer remains convinced that the PECAM1 immunostaining in Figure 1A is not convincing, even if it appears in the same channel as mT. By this stage of development, there is an extensive blood vascular network throughout embryonic dorsal skin which is not obvious in the figure.

Figure 2B: Further information should be provided in the Methods/Figure Legend to explain how "Normalised VEGFR3 expression" was calculated, one might presume area or pixels of VEGFR3 immunostaining per unit of total tissue area?

Figure Legend 5: The sentence beginning "In A-C", "show the direction" should read "arrows show the direction".

Second revision

Author response to reviewers' comments

Author Responses

We would like to thank the reviewer for their insightful suggestions and comments, which have significantly improved our paper.

Reviewer 1 Comments for the Author:
A few minor comments to be addressed:

© 2024. Published by The Company of Biologists under the terms of the Creative Commons Attribution License (https://creativecommons.org/licenses/by/4.0/).

This reviewer remains convinced that the PECAM1 immunostaining in Figure 1A is not convincing, even if it appears in the same channel as mT. By this stage of development, there is an extensive blood vascular network throughout embryonic dorsal skin which is not obvious in the figure.

We appreciate the reviewer's concern regarding the PECAM1 staining and agree that the display of blood vessels in this image is suboptimal. However, we think that, despite the background caused by the mT signal, blood vessels are clearly recognizable. Please see higher magnification images of the PECAM1/mT channel below. We would like to point out that at present we, unfortunately, cannot repeat this experiment. Repeating the antibody labeling would, since the image shows a whole-mount skin specimen, involve the generation of new tamoxifen-induced Rosa26$^{mTmG/+}$ Osr1$^{GCE/+}$ fetuses. This, however, is prevented as the licence issued by the respective authority (LaGeSo Berlin) to perform tamoxifen injections in this genotype has expired in the meantime. In this light, we hope the present image can be accepted.

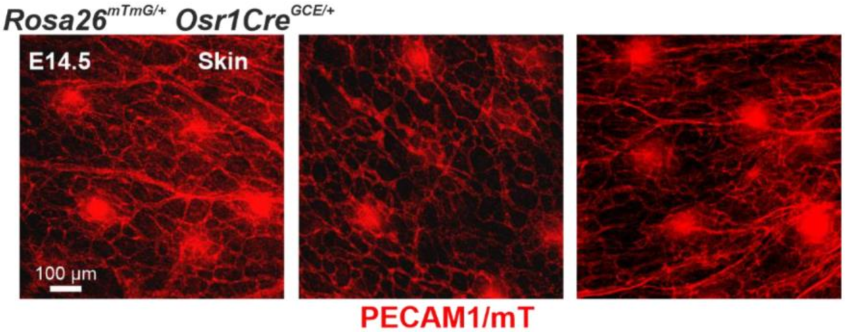

Figure 2B: Further information should be provided in the Methods/Figure Legend to explain how "Normalised VEGFR3 expression" was calculated, one might presume area, or pixels of VEGFR3 immunostaining per unit of total tissue area?

We thank the reviewer for the comment. We have used the Image J algorithm "integrated density" to quantify the sum of pixels per region of VEGFR3 staining. Each value was normalized to the average obtained from wild type sections. We have included this information in the Method part.

"In figure 2B, 4-5 regions along the rostro-caudal axis of the back from 4-5 consecutive 100µm sagittal sections were quantified per embryo. VEGFR3 expression was quantified using the Image J algorithm "integrated density" as the sum of pixels per region of VEGFR3 staining. For normalization, each value was normalized to the average value obtained from wild type sections."

Figure Legend 5: The sentence beginning "In A-C", "show the direction" should read "arrows show the direction".
This sentence in the figure legend has been adapted.

Third decision letter

MS ID#: DEVELOP/2024/202747

MS TITLE: Mesenchymal Osr1+ cells regulate embryonic lymphatic vessel formation

AUTHORS: Pedro Vallecillo Garcia, Mira Nicola Kuehnlein, Mickael Orgeur, Nils Rouven Hansmeier, Georgios Kotsaris, Bernd Timmermann, Claudia Giesecke-Thiel, Rene Haegerling, Sigmar Stricker, and Zarah Meisen
ARTICLE TYPE: Research Article

© 2024. Published by The Company of Biologists under the terms of the Creative Commons Attribution License (https://creativecommons.org/licenses/by/4.0/).

I am happy to tell you that your manuscript has been accepted for publication in Development, pending our standard publication integrity checks.

© 2024. Published by The Company of Biologists under the terms of the Creative Commons Attribution License (https://creativecommons.org/licenses/by/4.0/).
